# BCL2 expression is enriched in advanced prostate cancer with features of lineage plasticity

**DOI:** 10.1172/JCI179998

**Published:** 2024-09-17

**Authors:** Daniel Westaby, Juan M. Jiménez-Vacas, Ines Figueiredo, Jan Rekowski, Claire Pettinger, Bora Gurel, Arian Lundberg, Denisa Bogdan, Lorenzo Buroni, Antje Neeb, Ana Padilha, Joe Taylor, Wanting Zeng, Souvik Das, Emily Hobern, Ruth Riisnaes, Mateus Crespo, Susana Miranda, Ana Ferreira, Brian P. Hanratty, Daniel Nava Rodrigues, Claudia Bertan, George Seed, Maria de Los Dolores Fenor de La Maza, Christina Guo, Juliet Carmichael, Rafael Grochot, Khobe Chandran, Anastasia Stavridi, Andreas Varkaris, Nataly Stylianou, Brett G. Hollier, Nina Tunariu, Steven P. Balk, Suzanne Carreira, Wei Yuan, Peter S. Nelson, Eva Corey, Michael Haffner, Johann de Bono, Adam Sharp

**Affiliations:** 1 The Institute of Cancer Research, London, United Kingdom.; 2 The Royal Marsden NHS Foundation Trust, Sutton, United Kingdom.; 3 Fred Hutchinson Cancer Center, Seattle, Washington, USA.; 4 Beth Israel Deaconess Medical Center, Boston, Massachusetts, USA.; 5 Australian Prostate Cancer Research Centre–Queensland, Centre for Genomics and Personalised Health, School of Biomedical Sciences, Faculty of Health, Queensland University of Technology, Brisbane, Queensland, Australia.; 6 University of Washington, Seattle, Washington, USA.

**Keywords:** Oncology, Apoptosis, Prostate cancer

## Abstract

The widespread use of potent androgen receptor signaling inhibitors (ARSIs) has led to an increasing emergence of AR-independent castration-resistant prostate cancer (CRPC), typically driven by loss of AR expression, lineage plasticity, and transformation to prostate cancers (PCs) that exhibit phenotypes of neuroendocrine or basal-like cells. The anti-apoptotic protein BCL2 is upregulated in neuroendocrine cancers and may be a therapeutic target for this aggressive PC disease subset. There is an unmet clinical need, therefore, to clinically characterize BCL2 expression in metastatic CRPC (mCRPC), determine its association with AR expression, uncover its mechanisms of regulation, and evaluate BCL2 as a therapeutic target and/or biomarker with clinical utility. Here, using multiple PC biopsy cohorts and models, we demonstrate that BCL2 expression is enriched in AR-negative mCRPC, associating with shorter overall survival and resistance to ARSIs. Moreover, high BCL2 expression associates with lineage plasticity features and neuroendocrine marker positivity. We provide evidence that BCL2 expression is regulated by DNA methylation, associated with epithelial-mesenchymal transition, and increased by the neuronal transcription factor ASCL1. Finally, BCL2 inhibition had antitumor activity in some, but not all, BCL2-positive PC models, highlighting the need for combination strategies to enhance tumor cell apoptosis and enrich response.

## Abstract 2

In this study we identified BCL2 as a marker that classifies a subset of aggressive prostate cancers supporting the development of treatment approaches targeting apoptotic resistance pathways.

## Introduction

Prostate cancer (PC) is the most frequently diagnosed male-related cancer in the Western world (1). In Europe, 1% of all men die from this disease (1, 2). PC is largely driven by the androgen receptor (AR), and androgen deprivation therapy (ADT) is the cornerstone of treatment for advanced disease (3, 4). However, ADT resistance invariably occurs, and tumors progress from castration-sensitive PC (CSPC) to castration-resistant PC (CRPC) (5). Metastatic CRPC (mCRPC) is a lethal malignancy with an overall survival (OS) of 2–3 years (6, 7). Although next-generation AR signaling inhibitors (ARSIs) improve OS in metastatic CSPC and CRPC, the tumors inevitably develop resistance (5).

Most commonly, treatment resistance is driven by reactivation of AR signaling (5). However, intensive selective pressure from the use of ARSIs has resulted in an increasing emergence of PCs that adapt to survive without AR signaling, often with loss of AR expression, lineage plasticity, and transformation to PCs that exhibit phenotypes of neuroendocrine or basal-like cells (5, 8–10). Neuroendocrine-like/basal tumors respond poorly to endocrine therapies and are associated with poor prognosis, highlighting an urgent clinical need to develop effective therapeutic strategies for this type of CRPC (11, 12).

The anti-apoptotic protein BCL2 has been reported to be upregulated in various neuroendocrine cancers, including small-cell neuroendocrine CRPC (13). BCL2 promotes cell survival by sequestering pro-apoptotic proteins, including the pore-forming effector BAX, preventing mitochondrial outer membrane permeabilization (14). The BCL2 inhibitor venetoclax (ABT-199) has received FDA approval for chronic lymphocytic leukemia and acute myeloid leukemia, with clinical trials in solid tumors under way (15–17). AR activity suppresses BCL2 expression, with AR inhibition (enzalutamide) reported to upregulate BCL2 (18, 19). In androgen-dependent LNCaP cells, BCL2 upregulation is required for progression to an androgen-independent state (20). Furthermore, BCL2 is upregulated in several CRPC models with primary or acquired resistance to enzalutamide (18). Thus, BCL2 targeting has been proposed as a therapeutic strategy in neuroendocrine PC, and additionally as an approach to prevent or delay resistance to ARSIs in AR-dependent disease (13, 18, 21).

The objectives of this study were to characterize AR expression in advanced PC, to determine its association with BCL2, to elucidate their clinical significance, and to uncover mechanisms of regulation, with the aim of exploring whether BCL2 could represent a valid therapeutic target and/or biomarker (prognostic and/or predictive).

## Results

### Loss of AR protein emerges with castration resistance in a subset of mCRPC and associates with shorter OS.

**Figure 1:**
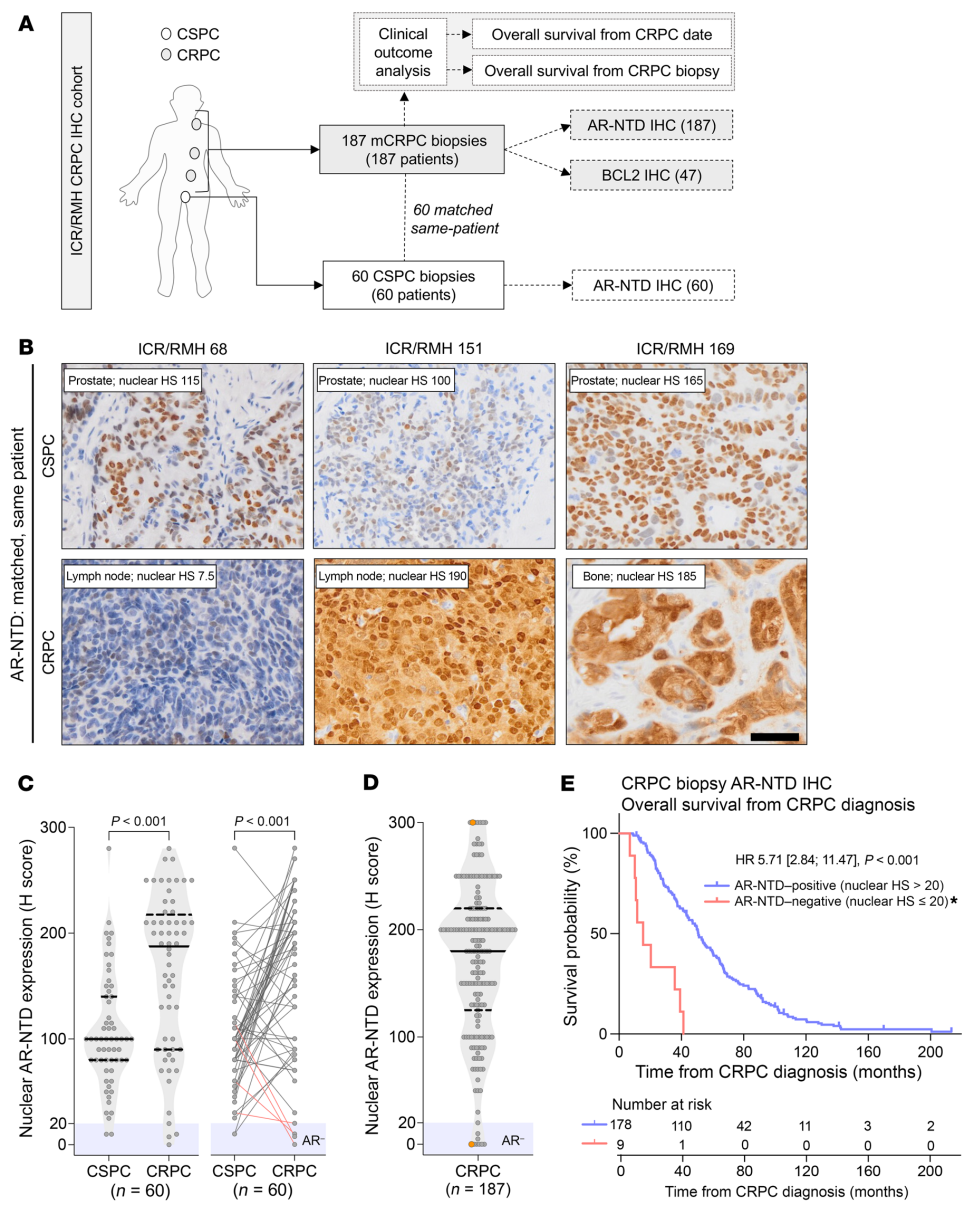
Loss of AR protein expression occurs in a small subset of CRPC and associates with poor prognosis. (**A**) Summary of clinical samples analyzed for the ICR/RMH CRPC IHC patient cohort. A total of 187 mCRPC biopsies and 60 matched same-patient CSPC biopsies were analyzed. Clinical outcome data, including OS from CRPC diagnosis and CRPC biopsy date, were collected for all 187 patients. (**A**–**D**) IHC for nuclear AR-NTD was performed in 187 CRPC biopsies and 60 matched, same-patient CSPC tumors. (**B**) Representative micrographs for matched, same-patient samples are shown with examples of emergent loss (patient 68) and increased (patient 151) and stable (patient 169) AR-NTD protein expression. Scale bar: 50 μm. HS, H score. (**C** and **D**) Nuclear AR-NTD protein expression (H score) in 60 matched, same-patient CSPC and CRPC tumors (**C**) and 187 CRPC tumors (**D**). Medians and interquartile ranges (IQRs) are shown. Wilcoxon’s matched-pairs signed-rank test was used to determine statistical significance. Red lines highlight cases of AR-NTD–negative CRPC and their matched CSPC sample. The threshold for AR negativity (H score ≤ 20) is highlighted in blue. A heterogeneous case with 2 tumor cell populations (AR positive and AR negative) is highlighted in orange and included twice. (**E**) Kaplan-Meier OS curves from time of CRPC diagnosis, split by AR-positive (H score > 20) and AR-negative (H score ≤ 20) tumors. HR with 95% CIs and *P* value for log-rank test are shown. *The heterogeneous case is included in the AR-NTD–negative group.

To elucidate the expression of the AR in mCRPC and investigate changes with the development of castration resistance, we performed immunohistochemistry (IHC) for the AR N-terminal domain (AR-NTD) on 187 mCRPC biopsies, as well as 60 matched, same-patient CSPC samples (Figure 1, A and B). Consistent with previous studies, AR expression increased with progression from CSPC to CRPC (Wilcoxon’s matched-pairs signed-rank test, *P* < 0.001) (Figure 1C) (22, 23). In contrast, in a small subset of PCs, AR expression was lost (nuclear AR-NTD H score ≤ 20) with the emergence of castration resistance (7%, 4/60) (Figure 1, B and C, red lines). Overall, 4.8% (9/187) of all mCRPC biopsies studied had loss of AR expression (Figure 1D). Notably, 72% (134/187) of patients had received a second-generation ARSI prior to their mCRPC biopsy (Supplemental Table 1; supplemental material available online with this article; https://doi.org/10.1172/JCI179998DS1). AR expression was lower in mCRPC liver biopsies than in mCRPC lymph node and prostate biopsies (*P* = 0.007 and *P* = 0.017, respectively, Tukey’s test) (Supplemental Figure 3A). Next, we evaluated the prognostic value of AR status; patients with AR-negative CRPC had significantly shorter OS, both from the time of CRPC diagnosis (median OS: 15.4 vs. 50.9 months; hazard ratio [HR]: 5.71, 95% confidence intervals [CI] 2.84–11.47, *P* < 0.001) (Figure 1E) and from the time of CRPC biopsy (median OS: 2.6 vs. 11.2 months; HR: 2.62, 95% CI 1.33–5.17, *P* = 0.004) (Supplemental Figure 3B).

### BCL2 protein expression is enriched in AR-negative mCRPC and associates with shorter OS.

**Figure 2:**
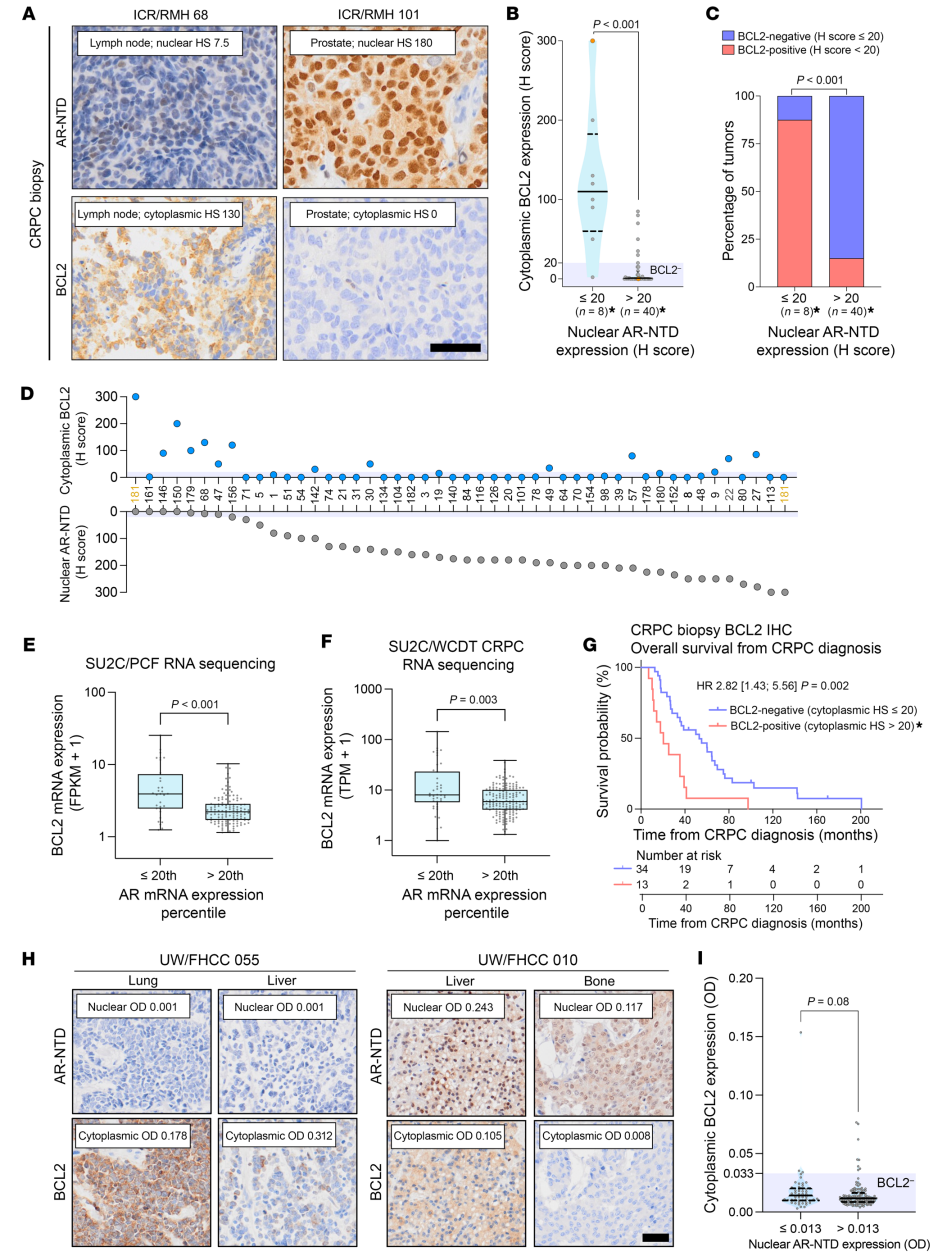
BCL2 protein expression is enriched in AR-negative disease and associates with poor prognosis. (**A**–**D**) IHC for BCL2 was performed on 47 CRPC biopsies, with preexisting AR-NTD (ICR/RMH CRPC IHC cohort). (**A**) Representative micrographs are shown. Scale bar: 50 μm. HS, H score. (**B**) Cytoplasmic BCL2 expression split by AR expression status: AR negative (AR-NTD H score ≤ 20, *n* = 8) and AR positive (AR-NTD H score > 20, *n* = 40). Median and IQRs are shown. Mann-Whitney *U* test was used. (**C**) Percentage of BCL2-positive (H score > 20) and BCL2-negative (H score ≤ 20) tumors split by AR expression status as above. Fisher’s exact test was used. *The heterogeneous case with 2 tumor cell populations (AR-positive/BCL2-negative and AR-negative/BCL2-positive) is included twice (highlighted orange). (**D**) H scores for nuclear AR-NTD and cytoplasmic BCL2 expression. (**E** and **F**) *BCL2* mRNA expression in AR-low (*AR* mRNA ≤20th percentile) and AR-high (*AR* mRNA >20th percentile) mCRPC in the SU2C/PCF (*n* = 210) (**E**) and SU2C/WCDT (*n* = 159) (**F**) cohorts. Medians and IQRs are shown. Mann-Whitney *U* test was used. (**G**) Kaplan-Meier survival curves from time of CRPC diagnosis, split by BCL2-positive and BCL2-negative tumors. HR with 95% CIs and *P* value for log-rank test are shown. *The heterogeneous case is included in the BCL2-positive group. (**H** and **I**) AR-NTD and BCL2 IHC was performed on 485 spatially separated samples from 177 mCRPC sites taken at rapid autopsy (58 patients). (**H**) Representative micrographs with examples of intrapatient inter-metastatic site heterogeneity. Scale bar: 50 μm. (**I**) Cytoplasmic BCL2 expression (OD) split by AR-negative (OD ≤ 0.013, *n* = 50) and AR-positive (OD > 0.013, *n* = 127) tumors. Average OD scores for all samples from each mCRPC tissue were used for this analysis. The threshold for BCL2 negativity (OD ≤ 0.033) is highlighted in blue. Median and IQRs are shown. Mann-Whitney *U* test was used to determine statistical significance.

BCL2 IHC was performed on 47 of 187 mCRPC biopsies with preexisting AR-NTD IHC status to investigate its association with AR-negative disease (Figure 1A and Figure 2A). These 47 biopsies were enriched for AR-negative tumors (8/47, 17%). BCL2 positivity, defined as a cytoplasmic BCL2 H score greater than 20, was observed in 28% (13/47) of the biopsies. AR-negative tumors had significantly higher cytoplasmic BCL2 immunoreactivity (Mann-Whitney *U* test, *P* < 0.001) (Figure 2B). BCL2 positivity was observed in 88% (7/8) of AR-negative tumors compared with 15% (6/40) of AR-positive tumors (Fisher’s exact test, *P* < 0.001) (Figure 2, C and D). However, although the 6 highest BCL2-expressing tumors were AR negative, 46% (6/13) of BCL2-positive tumors were AR positive (Figure 2, B and D). There were no significant differences in BCL2 expression between biopsy sites, though the analysis was limited by small sample size (Supplemental Figure 4A). BCL2 expression was also enriched in AR-negative tumors (optical density [OD] score ≤ 0.013) in the LuCaP patient-derived xenograft (PDX) series (Mann-Whitney *U* test, *P* < 0.001) (Supplemental Figure 5, A–D). BCL2 positivity (OD > 0.033) was observed in 71% (22/31) of AR-negative tumors and 22% (18/83) of AR-positive tumors (Fisher’s exact test, *P* < 0.001) (Supplemental Figure 5D). This finding was further validated with transcriptomic data, revealing significantly higher *BCL2* mRNA expression in AR-low mCRPC (≤ 20th percentile expression) in the Stand Up To Cancer (SU2C)/Prostate Cancer Foundation (PCF) and SU2C/West Coast Prostate Cancer Dream Team (WCDT) cohorts (Mann-Whitney *U* test, *P* < 0.001 and *P* = 0.003, respectively) (Figure 2, E and F). Importantly, patients with BCL2-positive CRPC had significantly shorter OS, both from the time of CRPC diagnosis (median OS: 20.4 vs. 53.0 months; HR: 2.82, 95% CI 1.43–5.56, *P* = 0.002) (Figure 2G) and from the time of CRPC biopsy (median OS: 8.8 vs. 13.4 months; HR: 2.03, 95% CI 1.03–4.01, *P* = 0.038) (Institute of Cancer Research [ICR]/Royal Marsden Hospital [RMH] CRPC IHC cohort) (Supplemental Figure 4B).

### Heterogeneity of AR and BCL2 protein expression in lethal CRPC.

Next, we used a rapid-autopsy CRPC cohort to investigate the degree of interpatient and intrapatient heterogeneity of AR and BCL2 expression (24). We analyzed 485 spatially separated samples from 177 mCRPC sites (58 patients). Heterogeneous expression was observed at 3 levels: interpatient, intrapatient inter-metastatic (between metastatic sites within the same patient), and intra-metastasis (within a metastatic site) (Figure 2H, Supplemental Figure 6A, and Supplemental Tables 7 and 8). The majority of the heterogeneity/variability was seen between patients (interpatient) and between metastatic sites (intrapatient inter-metastatic), but there was also a degree of intra-metastasis heterogeneity (Supplemental Tables 7 and 8). In terms of metastatic site/tissue type, there was higher AR expression in bone compared with liver metastases (Supplemental Figure 7, A and B, and Supplemental Table 9). Lung metastases had higher expression of BCL2 compared with all other sites of disease, though it should be noted that the sample size was small (Supplemental Figure 7, C and D, and Supplemental Table 10). When the average expression for each metastatic site was calculated, BCL2 expression was higher in AR-negative tumors, although this did not reach statistical significance (Mann-Whitney *U* test, *P* = 0.08) (Figure 2I). However, when binary AR-NTD expression (OD > 0.013) was added as a population-level effect to the Bayesian generalized linear multilevel model, this association was not observed, since, although BCL2 expression was higher, the threshold for BCL2 positivity was not reached in most AR-negative tumors (Supplemental Figure 6B).

### BCL2 positivity associates with resistance to ARSIs but not docetaxel, and with poor prognosis, in mCRPC.

**Figure 3:**
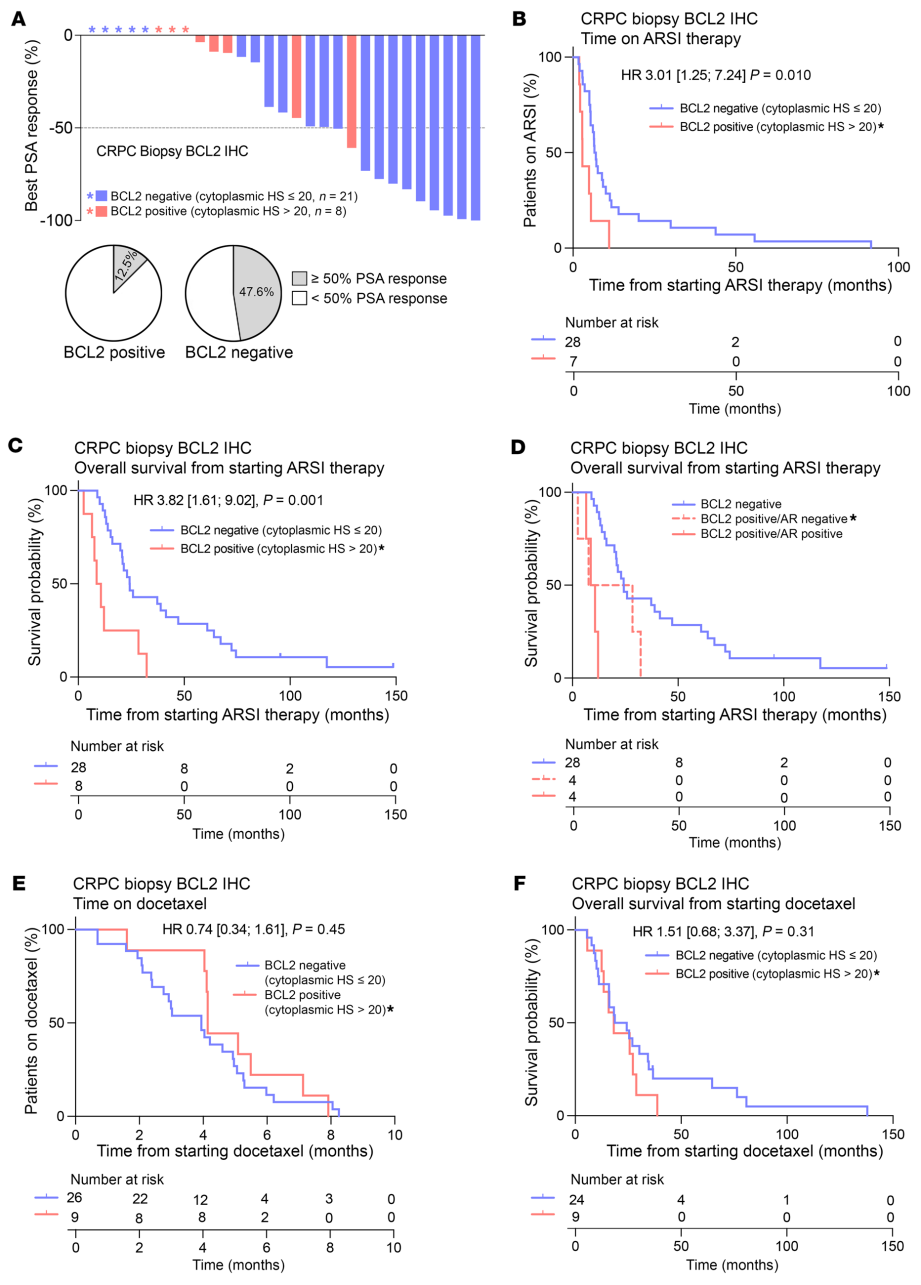
BCL2 positivity associates with resistance to ARSI and poor prognosis in mCRPC irrespective of AR expression status, but there is no difference in response to docetaxel. (**A**–**D**) Clinical outcome data, including best PSA response, time on ARSI, and OS from starting ARSI, were collected for 36 patients. (**A**) Top: Waterfall plot of greatest percentage fall in PSA from baseline for 29 patients treated with either abiraterone or enzalutamide, split by BCL2 negative (blue, *n* = 21) and BCL2 positive (red, *n* = 8). *No response. Bottom: Pie charts showing percentage of patients with BCL2-negative and BCL2-positive CRPC that had a ≥50% fall in PSA from baseline. (**B** and **C**) Kaplan-Meier curves, split by BCL2-positive (H score > 20) and BCL2-negative (H score ≤ 20) tumors, showing time on ARSI (**B**) and OS from initiation of ARSI (**C**). (**D**) Kaplan-Meier curves, split by BCL2-positive/AR-negative, BCL2-positive/AR-positive, and BCL2-negative, for OS from initiation of ARSI. *The heterogeneous case is included in the BCL2-positive (and BCL2-positive/AR-negative) group. (**E** and **F**) Clinical outcome data, including best PSA response, time on docetaxel, number of docetaxel cycles, and OS from starting docetaxel, were collected for 36 patients. Kaplan-Meier curves, split by BCL2-positive (H score > 20) and BCL2-negative (H score ≤ 20) tumors, showing time on docetaxel (**E**) and OS from initiation of docetaxel (**F**). HR with 95% CIs and *P* values for the log-rank test are shown for Kaplan-Meier curves.

Next, we analyzed clinical data to elucidate whether BCL2 positivity associates with resistance to ARSI in CRPC. ARSI response data were available for 36 patients with BCL2 status on their CRPC biopsies (Supplemental Figure 8A). All patients received either abiraterone or enzalutamide. A ≥50% fall in prostate-specific antigen (PSA) was observed in 47.6% (10/21) of patients with BCL2-negative disease, compared with 12.5% (1/8) with BCL2 positivity (Figure 3A). Furthermore, the duration of ARSI therapy and OS from initiation of therapy were significantly shorter in patients with BCL2-positive disease (median time on therapy: 6.8 vs. 2.9 months; HR: 3.01, 95% CI 1.25–7.24, *P* = 0.010; median OS: 24.3 vs. 9.7 months; HR: 3.82, 95% CI 1.61–9.02, *P* = 0.001) (Figure 3, B and C). Interestingly, the differences in response rate, time on therapy, and OS in BCL2-positive disease were observed irrespective of AR status (Figure 3D and Supplemental Figure 8, B and C), suggesting that BCL2 positivity may denote ARSI resistance regardless of AR expression status.

Docetaxel outcome data were available for 36 patients with BCL2 status on their mCRPC biopsies (Supplemental Figure 9A). There was no significant difference in time on treatment, OS from initiation of therapy, PSA response, or number of docetaxel cycles between BCL2-positive and BCL2-negative disease (Figure 3, E and F, and Supplemental Figure 9, B–G), supporting that patients with BCL2-positive mCRPC may derive more clinical benefit from docetaxel compared with ARSIs.

### The molecular landscape of BCL2-positive mCRPC.

**Figure 4:**
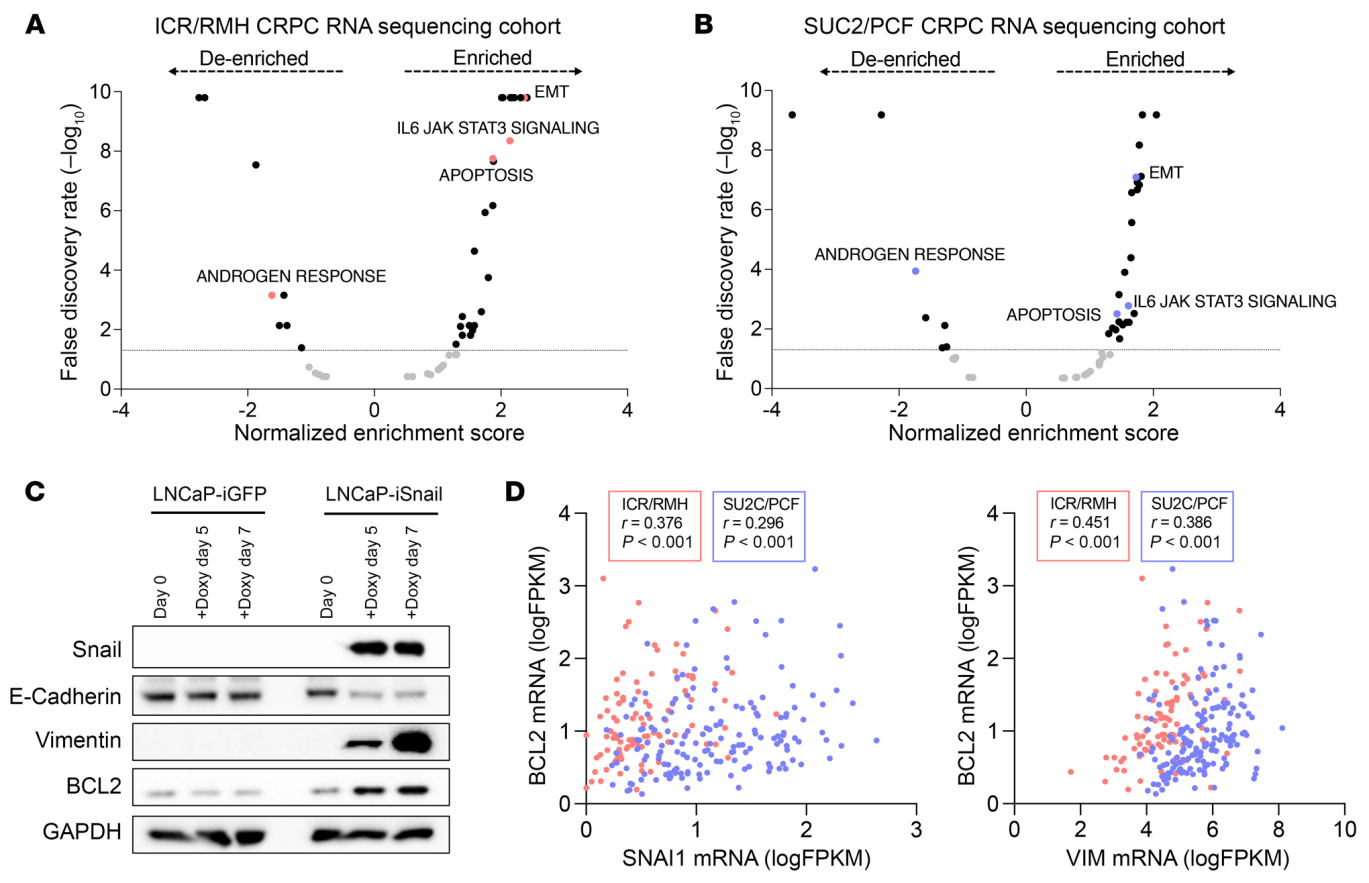
BCL2 expression associates with pathways implicated in lineage plasticity and is upregulated by Snail overexpression. (**A** and **B**) Gene set enrichment analysis (GSEA) using the “hallmark molecular signatures” was performed for *BCL2* mRNA expression in the ICR/RMH (*n* = 95) (**A**) and SU2C/PCF (*n* = 159) (**B**) CRPC RNA sequencing cohorts. Volcano plots show the false discovery rate–corrected *P* values (–log_10_) against the normalized enrichment score. (**C**) Western blot showing protein expression of Snail, E-cadherin, BCL2, and vimentin in untreated (day 0) LNCaP-iGFP and LNCaP-iSnail, and cells treated for 5 and 7 days with doxycycline. GAPDH was used as a loading control. The experiment was performed in 2 biological replicates (see Supplemental Figure 10D). (**D**) Correlation between *BCL2* and *SNAI1* mRNA expression (left), and between *BCL2* and *VIM* mRNA expression (right), in the ICR/RMH and SU2C/PCF data sets. Spearman’s correlation was used for statistical analysis.

To investigate the molecular landscape of BCL2-positive mCRPC, we performed gene set enrichment analysis (GSEA) using the “hallmark molecular signatures” from the Molecular Signatures Database (MSigDB, Broad Institute) for *BCL2* expression in the ICR/RMH and SU2C/PCF RNA sequencing data sets (Figure 4, A and B, Supplemental Figure 10, A and B, and Supplemental Tables 11 and 12) and mCRPC biopsy RNA sequencing data sets (23, 25). In accordance with our previous findings, the “androgen response” signature was de-enriched, while the “apoptosis” signature was enriched with increasing *BCL2* mRNA expression (Figure 4, A and B). In addition, *BCL2* mRNA expression was associated with “epithelial-mesenchymal transition” (EMT) and “IL-6/JAK/STAT3 signaling” (Figure 4, A and B).

To further investigate the link between EMT and BCL2, we used an LNCaP cell model with doxycycline-inducible expression of the master EMT transcription factor Snail (LNCaP-iSnail) (26). Robust Snail protein induction was observed with doxycycline treatment, which was accompanied by an upregulation of vimentin, downregulation of E-cadherin, and a shift to a mesenchymal morphology, as expected with EMT (Figure 4C and Supplemental Figure 10, C and D) (27). Induction of Snail resulted in an increase in BCL2 protein expression, while no changes were observed with the inducible GFP control (LNCaP-iGFP) (Figure 4C and Supplemental Figure 10D). In keeping with these findings, *SNAI1* and *VIM* mRNA expression were significantly positively correlated with BCL2 expression in both mCRPC data sets (ICR/RMH *r* = 0.376, *P* <0.001, and *r* = 0.451, *P* < 0.001, respectively; SU2C/PCF *r* = 0.296, *P* < 0.001, and *r* = 0.386, *P* < 0.001, respectively) (Figure 4D).

**Figure 5:**
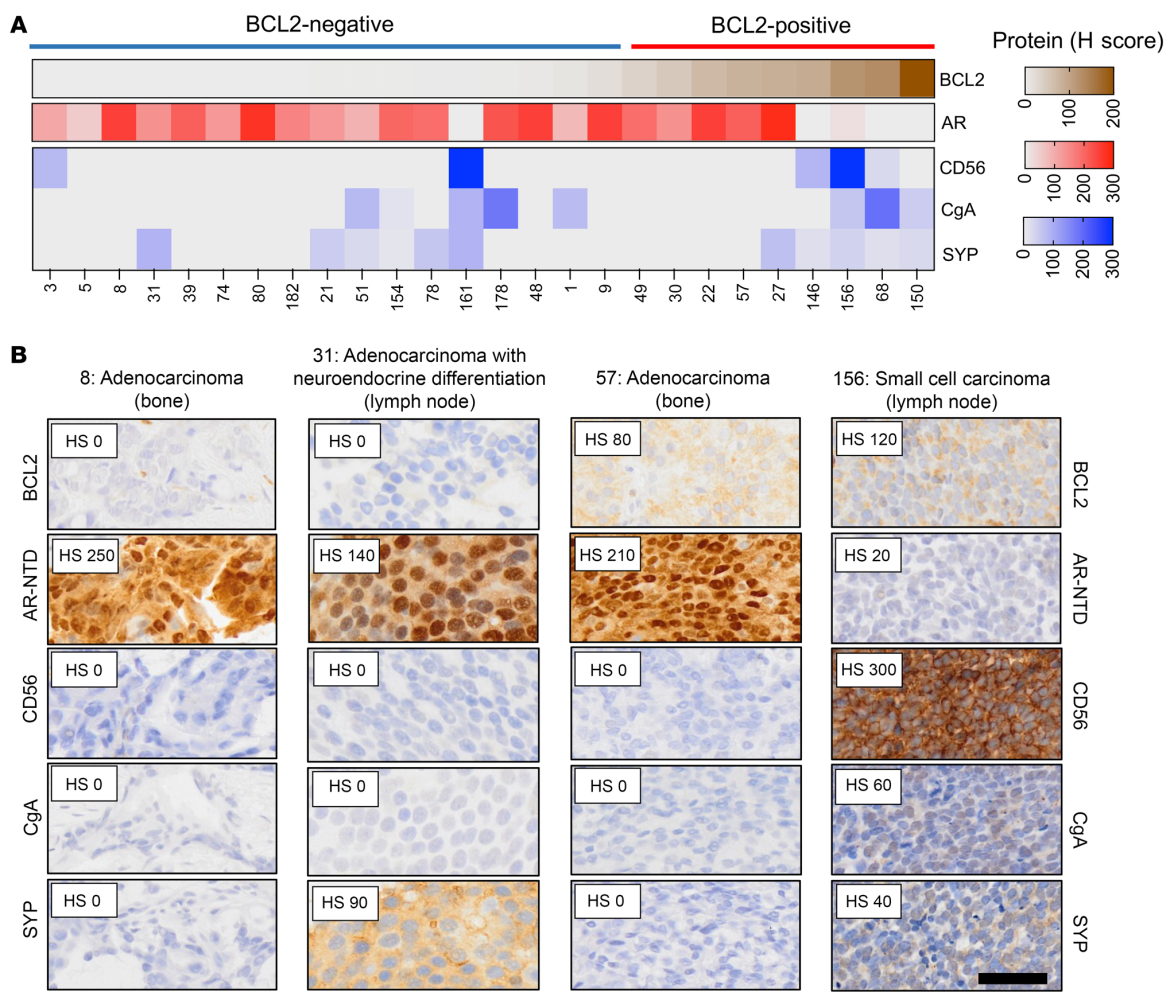
High BCL2 expression associates with neuroendocrine marker positivity. (**A** and **B**) IHC for CD56, chromogranin A (CgA), and synaptophysin (SYP) was undertaken in 26 mCRPC biopsies with preexisting BCL2 and AR IHC. (**A**) Heatmap depicting protein expression (H score) for AR, BCL2, CD56, CgA, and SYP. (**B**) Representative IHC micrographs are shown. Scale bar: 50 μm. HS, H score.

Having shown that BCL2 expression associates with AR loss and with pathways implicated in stemness and lineage plasticity, we performed IHC for putative neuroendocrine markers (CD56, chromogranin A, and synaptophysin) on a subset of mCRPC biopsies (*n* = 26) (28). The 5 tumors with the highest BCL2 protein expression had concurrent expression of at least 1 neuroendocrine marker; 3 were identified as small-cell carcinomas and 2 as large-cell neuroendocrine carcinomas (Figure 5, A and B, and Supplemental Table 13). Importantly, there were 4 other BCL2-positive tumors, without neuroendocrine marker expression, which were identified as adenocarcinomas.

### BCL2 expression is regulated by DNA methylation and driven by the neuronal transcription factor ASCL1.

**Figure 6:**
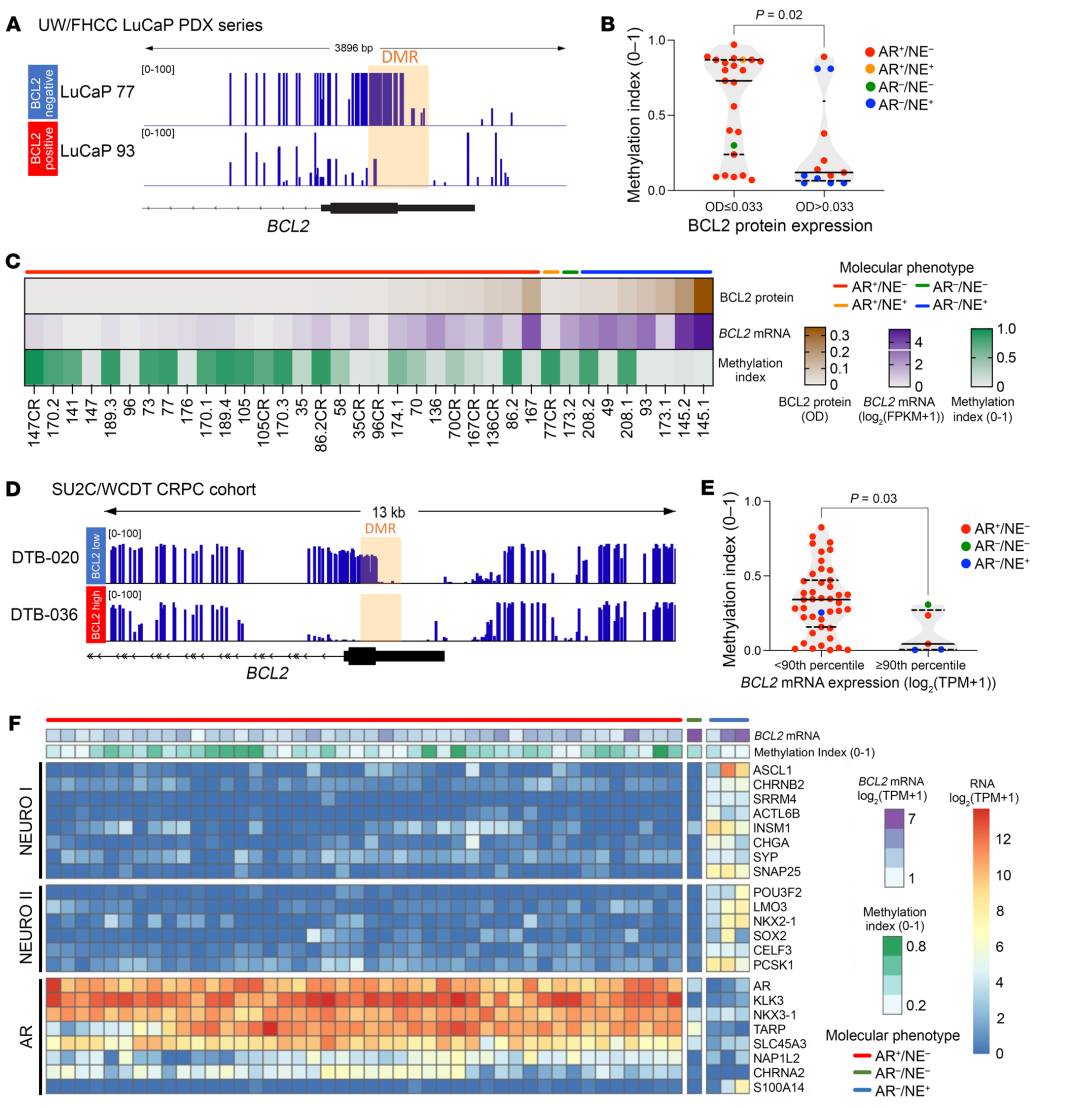
BCL2 expression is regulated by DNA methylation in mCRPC. (**A**–**C**) Whole-genome bisulfite sequencing (WGBS) in 36 LuCaP PDXs. (**A**) WGBS tracks from a BCL2-negative (LuCaP 77, blue) and a BCL2-positive (LuCaP 93, red) tumor. The differentially methylated region (DMR) on the BCL2 promoter is highlighted in orange. (**B**) Methylation index (0–1) split by BCL2-negative (OD ≤ 0.033, *n* = 23) and BCL2-positive (OD > 0.033, *n* = 13) tumors. Colors denote different molecular phenotypes. Mann-Whitney *U* test was used to determine statistical significance. (**C**) Heatmap depicting BCL2 protein expression, *BCL2* mRNA expression, and methylation index for the LuCaP PDXs. Tumors are organized by molecular phenotype (color bars) and then ordered by BCL2 protein expression. (**D**–**F**) WGBS in the SU2C/WCDT mCRPC patient cohort (*n* = 48). (**D**) WGBS tracks from a *BCL2*-low (DTB-020, blue) and a *BCL2*-high (DTB-036, red) tumor. (**E**) Methylation index (0–1) split by *BCL2*-low (<90th percentile mRNA expression, *n* = 43) and *BCL2*-high (≥90th percentile mRNA expression, *n* = 5) tumors. Colors denote different molecular phenotypes. Mann-Whitney *U* test was used to determine statistical significance. (**F**) Heatmap depicting *BCL2* mRNA expression and methylation index in SU2C/WCDT mCRPC cohort. Tumors are grouped by molecular phenotype as determined using the AR, NEURO I, and NEURO II gene expression sets.

To uncover further mechanisms of BCL2 expression regulation, we used whole-genome bisulfite sequencing of LuCaP PDX lines and identified a differentially methylated region (DMR) encompassing a CpG island on the BCL2 promoter. In the majority of BCL2-positive PDX models (protein OD > 0.033), the DMR was hypomethylated, and the average methylation index was lower in comparison with BCL2-negative models (*P* = 0.02) (Figure 6, A–C). This finding was validated in the publicly available SU2C/WCDT mCRPC patient data set, where tumors were split by *BCL2* mRNA expression (< 90th percentile vs. ≥ 90th percentile) (*P* = 0.03) (Figure 6, D–F).

The molecular subtype of SU2C/WCDT tumors was determined using the AR, NEURO I, and NEURO II gene expression sets, as previously described (Figure 6F) (29). The transcription factor *ASCL1*, known to promote lineage plasticity and neuroendocrine differentiation, was highly expressed in two AR-negative/neuroendocrine-positive (AR^–^NE^+^) tumors with concurrent high expression of *BCL2* (Figure 6F) (30, 31). In addition, transcriptome analysis of a cohort enriched in neuroendocrine PC showed *ASCL1* exclusively expressed in tumors with low or absent *AR* expression, all of which also expressed *BCL2* mRNA (Supplemental Figure 11) (32).

**Figure 7:**
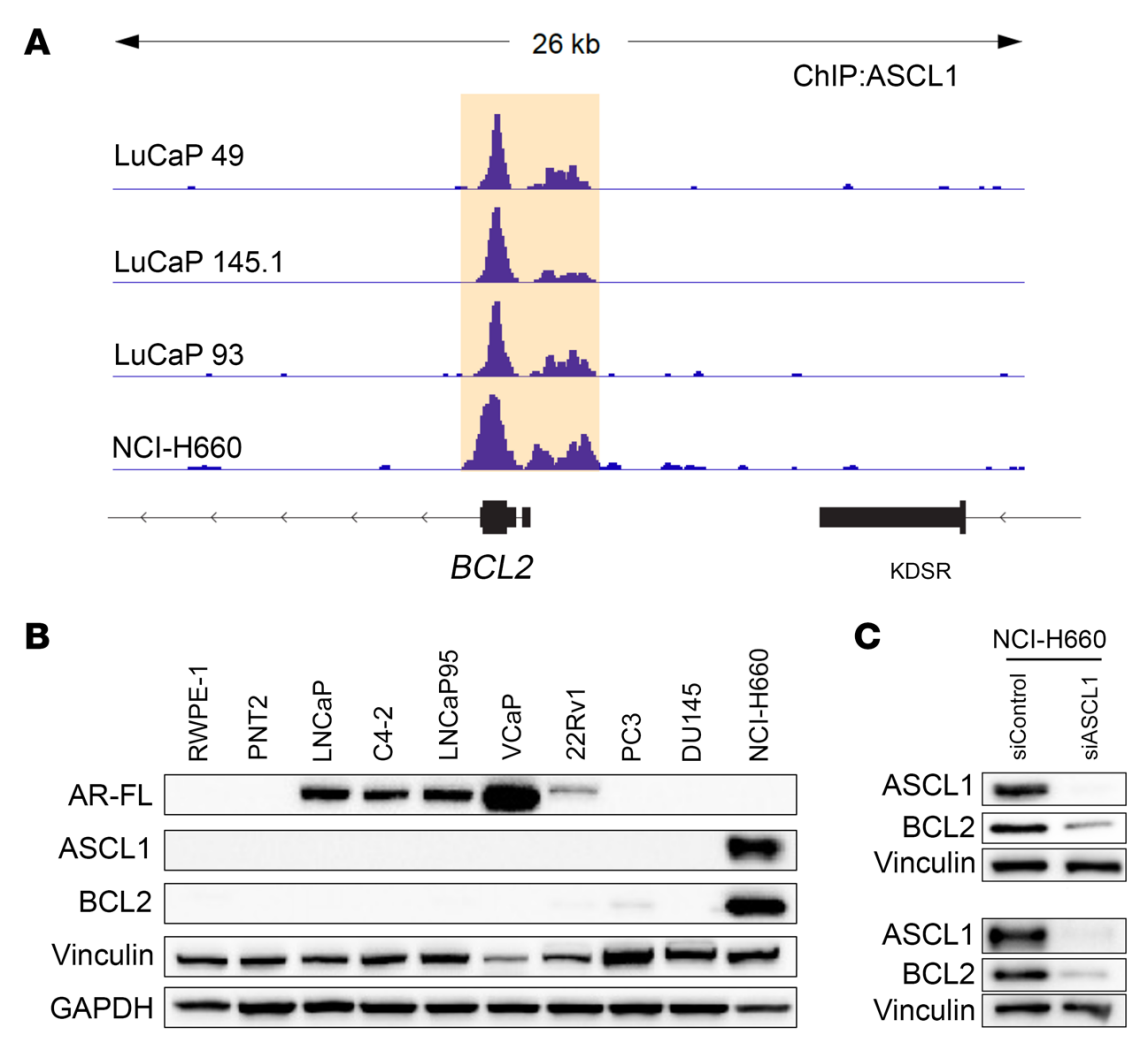
BCL2 expression is a transcriptional target of the neuronal lineage-guiding transcription factor ASCL1. (**A**) ASCL1 ChIP-Seq tracks in 4 AR-negative/BCL2-positive/NE-positive PC models: 3 LuCaP PDXs and the NCI-H660 cell line. (**B**) Western blot showing protein expression of full-length AR (AR-FL), ASCL1, and BCL2 in benign prostate (RWPE-1 and PNT2) and PC cell lines. Vinculin and GAPDH were used as a loading control. (**C**) Western blot showing the impact of ASCL1 knockdown (siRNA, 72 hours, 50 nM) on BCL2 protein expression in NCI-H660 cells. Two biological replicates are shown. Vinculin was used as a loading control.

Chromatin immunoprecipitation (ChIP) studies in four AR^–^NE^+^ BCL2-positive models (three LuCaP PDXs and the cell line NCI-H660) showed ASCL1 recruitment at the *BCL2* locus (Figure 7A) (31). Western blot analysis of benign and PC cell lines showed that BCL2 and ASCL1 were both highly expressed in NCI-H660 (Figure 7B). Furthermore, siRNA knockdown of ASCL1 led to downregulation of BCL2 in NCI-H660 cells (Figure 7C). Taken together, these data reveal *BCL2* as a transcriptional target of ASCL1 in neuroendocrine PC.

As discussed, although BCL2 expression is enriched in AR-negative mCRPC, there is a small subset of AR-positive tumors with concurrent expression of BCL2 (Figure 2, C, D, H, and I; Figure 6F; Supplemental Figure 5, A–D; and Supplemental Figure 6A). To investigate the phenotype of this subset, we interrogated transcriptomic data in an expanded SU2C/WCDT mCRPC cohort (*n* = 210), undertaking GSEA comparing BCL2-high/AR-high (*n* = 6) with BCL2-low/AR-high tumors (*n* = 99) (Supplemental Figure 12, A and B). Interestingly, there was negative enrichment of the “androgen response,” in keeping with the observation that BCL2 positivity associates with inferior response to ARSI and shorter OS (Supplemental Figure 12, A and B, and Supplemental Table 14). Taken together, these data support the hypothesis that BCL2 expression may denote AR independence irrespective of AR expression status.

### AR and BCL2 expression in a heterogeneous mCRPC PDX model.

**Figure 8:**
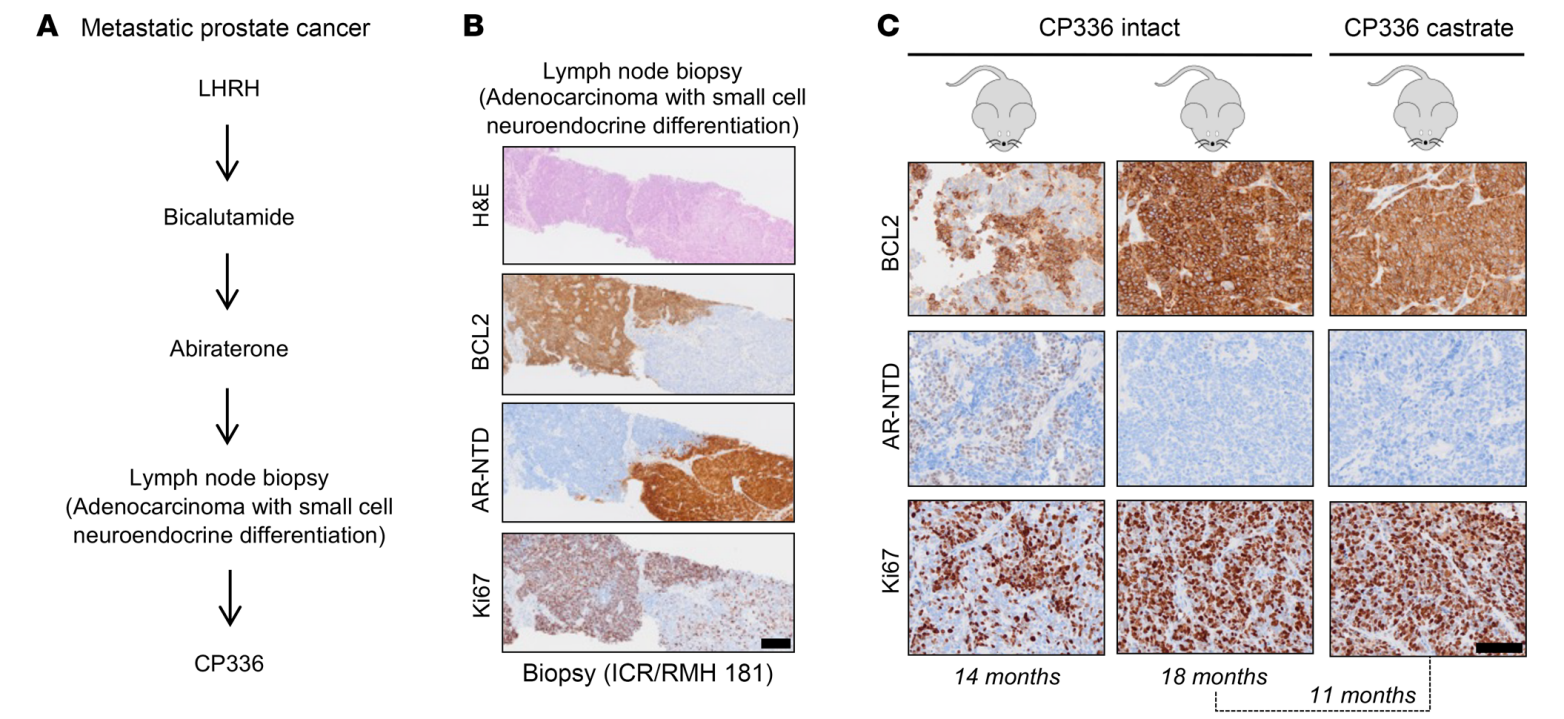
AR and BCL2 expression in a heterogeneous mCRPC PDX model. (**A**) Clinical history for patient 181, whose biopsy was used to generate CP336 PDX. CP336 PDX was developed from a lymph node biopsy containing adenocarcinoma with small-cell neuroendocrine differentiation, from a patient previously treated with agents targeting the AR signaling axis. (**B**) IHC for BCL2, AR-NTD, and Ki67 was performed on the lymph node biopsy used to develop CP336 PDX, revealing heterogeneous expression with 2 tumor cell populations: AR-negative/BCL2-positive and AR-positive/BCL2-negative. Representative micrographs are shown. Scale bar: 200 μm. (**C**) CP336-intact was passaged into castrated mice to develop CP336-castrate (CP336c). IHC was performed at different time points. Representative micrographs are shown. Scale bar: 100 μm.

To further elucidate the relationship of AR and BCL2 expression, we studied CP336, a PDX with 2 tumor cell populations: AR-positive/BCL2-negative and AR-negative/BCL2-positive (Figure 8, A–C). The CP336 PDX model was established from a lymph node biopsy containing adenocarcinoma with small-cell differentiation (ICR/RMH 181) and was taken from a patient with mCRPC previously exposed to luteinizing hormone–releasing hormone (LHRH) analog, bicalutamide, and abiraterone (Figure 8, A and B). CP336 was subsequently passaged from intact into castrated mice to generate a CP336-castrate PDX (CP336c) (Figure 8C). IHC performed on the patient biopsy and CP336/CP336c revealed that Ki67 was higher in the AR-negative/BCL2-positive tumor cell population (Figure 8, B and C). We observed predominance of the AR-negative/BCL2-positive cellular subpopulation in the CP336 model over serial passages (Figure 8C).

### Targeting BCL2 in BCL2-positive PC.

**Figure 9:**
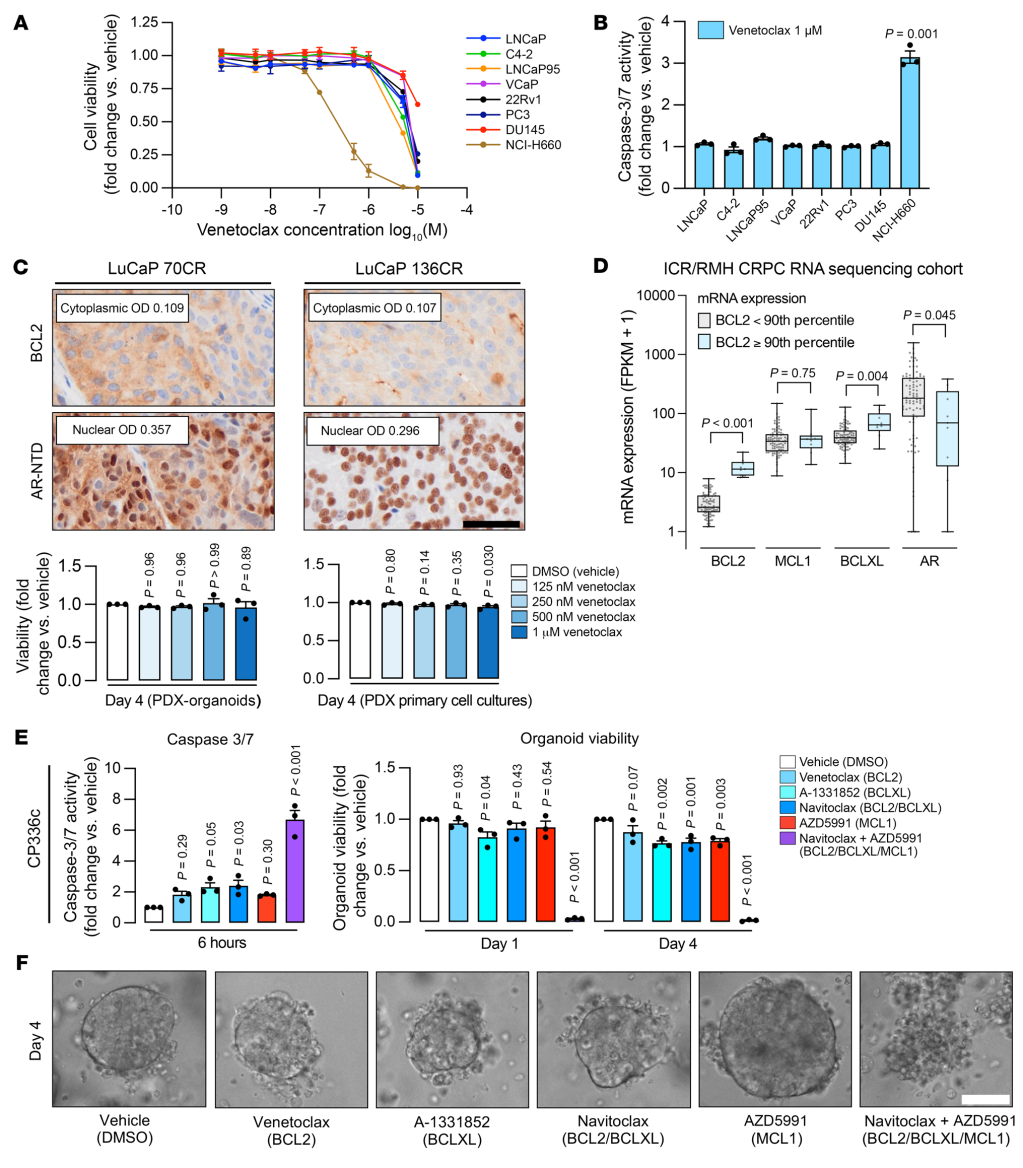
Targeting BCL2 in BCL2-positive PC. (**A**) Dose-response curves for PC cell lines treated with venetoclax. Cell viability was compared with vehicle (DMSO) in each cell line and evaluated at 72 hours using the CellTiter-Glo assay (Promega). (**B**) Caspase-3/7 activity (Caspase Glo-3/7 assay) was measured at 6 hours after treatment with venetoclax (1 μM) and compared with vehicle (DMSO). Experiments were performed in 3 biological replicates, each with 3 technical replicates. SEM is shown. The impact of venetoclax was compared against vehicle (DMSO) for each cell line by unpaired, 2-tailed *t* test. (**C**) IHC for cytoplasmic BCL2 and nuclear AR-NTD was performed on LuCaP 70CR and 136CR PDXs. Scale bar: 50 μm. LuCaP 70CR (PDX-O) and 136CR (primary cell culture) were treated with venetoclax (125, 250, and 500 nM and 1 μM) for 96 hours. Viability was determined by CellTiter-Glo 3D assay. Dunnett’s multiple-comparison test was used to determine statistical significance of each concentration versus the vehicle. (**D**) Transcriptome analyses associating *BCL2* mRNA expression (<90th percentile vs. ≥90th percentile) with *MCL1*, *BCLXL*, and *AR* mRNA expression (ICR/RMH CRPC RNA sequencing cohort, *n* = 95). Medians and IQRs are shown. Mann-Whitney *U* test was used to determine statistical significance. (**E** and **F**) CP336c PDX-organoids (PDX-O) were treated with vehicle (DMSO), venetoclax (1 μM), A-1331852 (100 nM), navitoclax (1 μM), AZD5991 (1 μM), and a combination of AZD5991 (1 μM) and navitoclax (1 μM). (**E**) The impact on caspase-3/7 activity (6 hours) and organoid viability (24 and 96 hours) was determined by Caspase Glo-3/7 3D and CellTiter-Glo 3D assays, respectively. The experiment was performed in biological triplicate with 5 technical replicates. SEM is shown. Dunnett’s multiple-comparison test was used to determine statistical significance of each drug versus the vehicle. (**F**) Representative microscopy images of CP336c PDX-O on day 4 after treatment. Scale bar: 50 μm.

To investigate whether BCL2 expression associates with response to BCL2 inhibition, eight PC cell lines (one BCL2 positive and seven BCL2 negative) were treated with the BCL2 inhibitor venetoclax at various concentrations (Figure 9, A and B). Cell viability analysis revealed a substantial downward shift in the dose-response curve for the BCL2-positive NCI-H660 cell line after 72 hours of treatment compared with the BCL2-negative lines (Figure 9A). Furthermore, there was a significant increase in caspase-3/7 activity at 6 hours after 1 μM of venetoclax (unpaired, 2-tailed *t* test, *P* = 0.001) (Figure 9B). Next, three BCL2-positive PDX-derived mCRPC models — CP336c (PDX-organoid [PDX-O]), LuCaP 70CR (PDX-O), and LuCaP 136CR (primary cell culture) — were treated in vitro with venetoclax; however, there was minimal antitumor activity (Figure 9, C, E, and F).

We hypothesized that redundancy between anti-apoptotic BCL2 family proteins contributes to venetoclax resistance; we performed IHC for BCLXL and MCL1 in the CP336/CP336c PDX lines and in two BCL2-positive mCRPC patient biopsies (Supplemental Figure 13, A and B). BCLXL and MCL1 were both highly expressed in CP336/CP336c (Supplemental Figure 13A). MCL1 was also expressed in both mCRPC biopsies, with BCLXL highly expressed in one (ICR/RMH 156) (Supplemental Figure 13B). RNA sequencing data from 3 independent mCRPC cohorts (ICR/RMH, SU2C/PCF, and SU2C/WCDT) revealed that tumors with high *BCL2* (mRNA expression ≥90th percentile) also expressed *MCL1* and *BCLXL* (Figure 9D and Supplemental Figure 13, C and D) (23, 25). In high-*BCL2*-expressing tumors, *BCLXL* expression was increased in the ICR/RMH cohort, and *MCL1* was increased in the SU2C/PCF and SU2C/WCDT cohorts (Mann-Whitney *U* test, *P* = 0.004, *P* = 0.017, and *P* = 0.022, respectively) (Figure 9D and Supplemental Figure 13, C and D). In support of our findings, in all 3 cohorts, *AR* mRNA expression was lower in tumors with high *BCL2* expression (Mann-Whitney *U* test, *P* = 0.045, *P* < 0.001, and *P* < 0.001, respectively) (Figure 9D and Supplemental Figure 13, C and D).

### Targeting BCL2, BCLXL, and MCL1 together triggers rapid apoptotic PC cell death.

To explore functional redundancy between the 3 main anti-apoptotic BCL2 proteins (BCL2, MCL1, and BCLXL), and to enrich response, we treated CP336c PDX-Os with A-1331852 (BCLXL inhibitor; 100 nM), navitoclax (BCL2/BCLXL inhibitor; 1 μM), AZD5991 (MCL1 inhibitor; 1 μM), and navitoclax/AZD5991 combined treatment (both at 1 μM). There was marked induction of apoptosis and significant reduction in CP336c organoid viability when all 3 main anti-apoptotic BCL2 proteins were targeted (Dunnett’s test, both *P* < 0.001) (Figure 9, E and F), suggesting that this may be an effective strategy to eliminate PC cells. This was recapitulated in 9 prostate cell lines (including benign models), though DU145 cells did not respond, as they are known to be resistant to apoptosis because of loss of the pore-forming effector protein BAX (Supplemental Figure 14, A and B) (33). Thus, since such a combined approach is likely to be toxic to normal cells, targeted drug delivery technologies (e.g., antibody-drug conjugates) may be required to abrogate toxicity and induce selective cancer cell lethality (34, 35).

## Discussion

Targeting of the AR signaling axis is the cornerstone of treatment for advanced PC, and AR signaling remains critically important in most CRPC progression. We show that a subset of mCRPC is AR negative, associating with markedly shorter OS, highlighting the urgent clinical need to develop new effective therapies for this PC subtype. This is arguably the most extensive clinically characterized IHC study of AR in mCRPC, including 424 biopsies from 245 patients in 2 independent cohorts, as well as multiple PDX models. Given its relative rarity, there are limited clinical series reporting outcomes for aggressive PC subtypes, and none of these stratify patients by AR expression status (11, 12). Although prior studies have described AR-negative mCRPC, they have not included longitudinal analyses. All the patients in our matched study had adenocarcinoma at the time of diagnosis, suggesting that loss of AR expression emerges over time due to treatment pressure. Potential mechanisms include lineage plasticity or selection of a preexisting AR-negative clone, a phenomenon demonstrated in our CP336 PDX model.

The anti-apoptotic protein BCL2 has been proposed as an attractive therapeutic target both to prevent resistance to AR inhibition in AR-dependent PC, and to target AR-independent neuroendocrine mCRPC (13, 18). The combination of enzalutamide with venetoclax has been evaluated in an early-phase clinical trial for mCRPC (ClinicalTrials.gov NCT03751436), but limited antitumor activity was observed; this may be explained by the lack of molecular characterization for patient selection, including the absence of BCL2 expression analysis (36). We show that BCL2 expression is enriched in AR-negative mCRPC, in keeping with previous data from primary PC samples (37). For what we believe is the first time, we demonstrate that BCL2 positivity associates with shorter OS and with resistance to ARSI therapy in mCRPC. This finding appeared to be independent of AR expression status but was based on a small number of cases and would need to be confirmed in further studies. Importantly, there was no significant difference in outcome with docetaxel treatment, suggesting that patients with BCL2-positive mCRPC may derive more clinical benefit from docetaxel compared with ARSI.

We have also demonstrated that *BCL2* mRNA expression in mCRPC associates with signaling pathways implicated in resistance to ARSI, including EMT and IL-6/JAK/STAT3 signaling (38–42). Recent data suggest that treatment resistance may involve increased JAK/STAT signaling and that targeting this pathway may overcome ARSI resistance (38, 42). We show that *BCL2* mRNA expression and *SNAI1* mRNA expression are positively correlated in mCRPC, and that BCL2 protein expression is upregulated by Snail in an LNCaP cell model with doxycycline-inducible expression of Snail. Snail is a key transcription factor that drives EMT, a process that may lead to therapy resistance (43–45). In addition, in accordance with findings in neuroendocrine lung cancer, we provide evidence that *BCL2* is a transcriptional target of ASCL1. ASCL1 is a transcription factor known to activate neuronal stem cell–like lineage programming and has been implicated in neuroendocrine PC (30, 31, 46). In keeping with this and previous studies, we show that high-BCL2-expressing tumors have concurrent expression of putative neuroendocrine markers (13, 28). Taken together, these data suggest that high-BCL2-expressing tumors have developed an EMT or neuroendocrine/basal phenotype. It remains to be seen whether EMT states lead to neuroendocrine/basal PC phenotypes.

It is important to note that a subset of BCL2-positive mCRPCs, ones with lower expression of BCL2, remained AR-positive/neuroendocrine marker–negative, suggesting that this process is not simply an on/off state. Despite this, these BCL2-positive/AR-positive tumors are associated with worse clinical outcomes and resistance to ARSIs. Furthermore, transcriptomic analysis revealed negative enrichment of the “androgen response” in this subset, suggesting that BCL2 positivity may denote AR independence irrespective of AR expression status. This suggests that BCL2 positivity can guide therapy decisions. However, given the small sample size, these findings should be interpreted with caution; further validation and functional studies are required.

Importantly, studies have shown BCL2-positive small-cell neuroendocrine cell lines to be more sensitive to BCL2 inhibition than AR-positive cell lines, with 2 of 5 (40%) CRPC small-cell neuroendocrine PDX models sensitive to navitoclax, which targets both BCLXL and BCL2 (13). Consistent with this, our studies supported that the BCL2-positive neuroendocrine PC cell line NCI-H660 was sensitive to venetoclax with induction of apoptosis; however, there was limited antitumor activity when BCL2-expressing PDX CRPC models were treated in vitro. However, the CP336c PDX-O, and numerous prostate cell lines, underwent rapid apoptosis when BCL2, MCL1, and BCLXL were cotargeted together, suggesting that resistance may be driven by functional redundancy between these proteins. Because of anticipated toxicity, targeting of these proteins together is unlikely to be tolerated without the use of cancer cell–specific drug delivery technologies (e.g., antibody-drug conjugates) (34, 35). Another approach is to target hyperactivated oncogenic pathways that regulate the expression or activity of anti-apoptotic BCL2 proteins (e.g., MAPK or PI3K pathways), thereby specifically targeting cancer cells (47–50). A further challenge is the observed AR and BCL2 heterogeneity, suggesting that combination strategies will be needed to eradicate coexisting clones to achieve clinical impact.

Limitations of this study include the retrospective nature of the clinical data collection. CRPC biopsies were taken at varied intervals from CRPC diagnosis, and exposure to different treatments was not controlled for. Although the clinical cohorts are large, given the rarity of the AR-negative and/or BCL2-positive subtype, the number of patients in this group is limited, especially when the BCL2-positive tumors are subdivided by AR status. Similarly, although we characterized a multitude of cell lines and PDXs, our BH3-mimetic experiments were limited to four BCL2-positive models. Finally, further studies are required to dissect the interaction between AR and BCL2, and to probe the functional role of BCL2 in this subset of mCRPC.

### Conclusions.

In summary, BCL2 expression is enriched in AR-negative mCRPC with features of lineage plasticity. BCL2-positive mCRPC is associated with worse clinical outcomes, appearing more sensitive to docetaxel than ARSI. Mechanistically, BCL2 expression is regulated by DNA methylation and increased by Snail and ASCL1. BCL2 inhibition has antitumor activity in some BCL2-positive PC models with a requirement for combination strategies to enhance response to therapy.

## Methods

### Sex as a biological variable

Given the disease etiology, sex was not considered as a variable.

### Patients and tissue samples

In the ICR/RMH and University of Washington (UW)/Fred Hutchinson Cancer Center (FHCC) cohorts, all patients had mCRPC. Clinical data and demographics were collected retrospectively from the RMH electronic patient record system (Supplemental Tables 1 and 2). For the treatment response cohorts, prior exposure to ARSI or docetaxel is shown in Supplemental Tables 3 and 4. Further methods are described in Supplemental Methods.

### Immunohistochemistry

IHC for AR-NTD and BCL2 was performed on sectioned tissue with at least 100 tumor cells, (reviewed by a histopathologist with expertise in PC) using M3562 (Dako) and M0887 (Dako) antibodies, respectively, as previously reported (21, 51). MCL1 and BCLXL IHC validation is described in Supplemental Figure 1. All IHC was performed at the ICR. AR negativity was defined as a nuclear AR-NTD H score of ≤20. BCL2 positivity was defined as a cytoplasmic BCL2 H score greater than 20. OD scores, generated by a pathologist-supervised machine learning algorithm (HALO AI, Indica Labs), were used for the UW/FHCC cohort and LuCaP patient-derived xenograft (PDX) series, allowing automated batch analysis of tissue microarrays. The equivalent cutoffs for AR-NTD OD (≤0.013) and BCL2 OD (>0.033) were calculated using simple linear regression equations from the correlation between H score and OD scores determined on a subset of samples (AR NTD H score = [442.25 × OD] + 14.457; BCL2 H score = [872.7 × OD] + 8.614) (Supplemental Figure 2, A and B). Quantification and image acquisition are described in Supplemental Methods.

### Western blotting

Western blotting methods are described in Supplemental Methods.

### PDX, PDX-organoid, and cell line studies

#### ICR/RMH patient-derived models.

The CP336 (and CP336c) PDX was derived from a human CRPC lymph node biopsy using the same methods as for CP50 and CP89 (52–54).

#### UW/FHCC patient-derived models.

LuCaP PDXs were established as previously described (55).

PDX, PDX-O, primary cell culture, and cell line studies are described in Supplemental Methods.

### Methylation analysis

#### LuCaP PDXs.

Genome-scale methylation analyses of LuCaP PDX DNAs were carried out using Infinium MethylationEPIC BeadChip arrays (Illumina) as described previously (56).

#### SU2C/WCDT cohort.

Whole-genome bisulfite sequencing and RNA sequencing data for the WCDT cohort were previously reported, and data were processed as described previously (57, 58). Further details are described in Supplemental Methods.

### siRNA transfection

NCI-H660 cells were transfected with ON-TARGETplus siRNA (SMARTpool, mixture of 4 siRNAs) (Dharmacon, Horizon) at 50 nM using 0.4% Lipofectamine RNAiMAX Reagent (Thermo Fisher Scientific) per the manufacturer’s guidelines. Nontargeting control (D-001810-10) and ASCL1 (L-0008307-00) siRNAs were used.

### Statistics

All statistical tests used are discussed in the results. Statistical tests included Wilcoxon’s matched-pairs signed-rank test, Kaplan-Meier, logrank, Mann-Whitney U test, Spearman’s correlation, Fisher’s exact test, Dunnett’s multiple-comparison test, and unpaired, 2-tailed *t* test and were performed using GraphPad Prism v9 or R v4.2.1. Specific details, including bioinformatic methods, can be found in Supplemental Methods.

### Study approval

#### ICR/RMH CRPC cohort.

All patients provided written informed consent and were enrolled in protocols approved by the RMH ethics review committee (reference 04/Q0801/60).

#### UW/FHCC CRPC IHC cohort.

Samples were obtained from patients who died of mCRPC and had signed written consent for a rapid autopsy as part of the Prostate Cancer Donor Program. Tissue collection was approved by the Institutional Review Board at UW.

#### ICR/RMH patient-derived models.

All mouse studies received approval from the ICR Animal Welfare and Ethical Review Body and were performed in accordance with the UK Animals (Scientific Procedures) Act of 1986.

#### UW/FHCC patient-derived models.

Studies were undertaken in accordance with an approved UW Institutional Animal Care and Use Committee protocol.

### Data availability

All data have been made available in the Supporting Data Values file. Further data access requests can be made to the corresponding author.

## Author contributions

DW, JMJV, IF, JDB, and A Sharp conceived and designed the study. DW, JMJV, IF, CP, BG, LB, AN, AP, RR, MC, SM, AF, DNR, CB, MDLDFDLM, CG, JC, RG, KC, JT, WZ, SD, EH, A Stavridi, AV, BPH, NT, EC, and MH acquired data. DW, JMJV, IF, BPH, JT, JR, DB, GS, SC, AL, and WY analyzed and interpreted data. DW, JMJV, IF, JDB, and A Sharp drafted the manuscript. DW, JMJV, IF, SPB, PSN, BGH, NS, MH, EC, JDB, and A Sharp critically revised the manuscript. DW, JMJV, DB, WY, BPH, AL, and JR performed statistical analysis. DW, JMJV, SPB, PSN, MH, EC, BGH, NS, JDB, and A Sharp acquired funding. SPB, PSN, MH, EC, JDB, and A Sharp supervised the study.

## Supplementary Material

Supplemental data

Unedited blot and gel images

Supporting data values

## Version 1 09/17/2024

Electronic publication

## References

[1] https://gco.iarc.fr/.

[2] Dyba T (2021). The European cancer burden in 2020: incidence and mortality estimates for 40 countries and 25 major cancers. Eur J Cancer.

[3] Wilding G (1992). The importance of steroid hormones in prostate cancer. Cancer Surv.

[4] Culig Z, Santer FR (2014). Androgen receptor signaling in prostate cancer. Cancer Metastasis Rev.

[5] Westaby D (2021). A new old target: androgen receptor signaling and advanced prostate cancer. Annu Rev Pharmacol Toxicol.

[6] Mehtala J (2020). Overall survival and second primary malignancies in men with metastatic prostate cancer. PLoS One.

[7] Moreira DM (2017). Predicting time from metastasis to overall survival in castration-resistant prostate cancer: results from SEARCH. Clin Genitourin Cancer.

[8] Bluemn EG (2017). Androgen receptor pathway-independent prostate cancer is sustained through FGF signaling. Cancer Cell.

[9] Mu P (2017). SOX2 promotes lineage plasticity and antiandrogen resistance in TP53- and RB1-deficient prostate cancer. Science.

[10] Beltran H (2019). The role of lineage plasticity in prostate cancer therapy resistance. Clin Cancer Res.

[11] Vlachostergios PJ (2017). Emerging variants of castration-resistant prostate cancer. Curr Oncol Rep.

[12] Wang HT (2014). Neuroendocrine Prostate Cancer (NEPC) progressing from conventional prostatic adenocarcinoma: factors associated with time to development of NEPC and survival from NEPC diagnosis—a systematic review and pooled analysis. J Clin Oncol.

[13] Corella AN (2020). Identification of therapeutic vulnerabilities in small-cell neuroendocrine prostate cancer. Clin Cancer Res.

[14] Westaby D (2021). Targeting the intrinsic apoptosis pathway: a window of opportunity for prostate cancer. Cancers (Basel).

[15] Souers AJ (2013). ABT-199, a potent and selective BCL-2 inhibitor, achieves antitumor activity while sparing platelets. Nat Med.

[16] Wei AH (2020). Venetoclax plus LDAC for newly diagnosed AML ineligible for intensive chemotherapy: a phase 3 randomized placebo-controlled trial. Blood.

[17] Roberts AW (2016). Targeting BCL2 with venetoclax in relapsed chronic lymphocytic leukemia. N Engl J Med.

[18] Li Q (2018). Linking prostate cancer cell AR heterogeneity to distinct castration and enzalutamide responses. Nat Commun.

[19] Huang H (2004). Androgens repress Bcl-2 expression via activation of the retinoblastoma (RB) protein in prostate cancer cells. Oncogene.

[20] Lin Y (2007). Up-regulation of Bcl-2 is required for the progression of prostate cancer cells from an androgen-dependent to an androgen-independent growth stage. Cell Res.

[21] Liang Y (2021). Emergence of enzalutamide resistance in prostate cancer is associated with BCL-2 and IKKB dependencies. Clin Cancer Res.

[22] Chen CD (2004). Molecular determinants of resistance to antiandrogen therapy. Nat Med.

[23] Abida W (2019). Genomic correlates of clinical outcome in advanced prostate cancer. Proc Natl Acad Sci U S A.

[24] Kumar A (2016). Substantial interindividual and limited intraindividual genomic diversity among tumors from men with metastatic prostate cancer. Nat Med.

[25] Fenor de la Maza MD (2022). Immune biomarkers in metastatic castration-resistant prostate cancer. Eur Urol Oncol.

[26] Stylianou N (2019). A molecular portrait of epithelial-mesenchymal plasticity in prostate cancer associated with clinical outcome. Oncogene.

[27] Kalluri R, Weinberg RA (2009). The basics of epithelial-mesenchymal transition. J Clin Invest.

[28] Epstein JI (2014). Proposed morphologic classification of prostate cancer with neuroendocrine differentiation. Am J Surg Pathol.

[29] Labrecque MP (2019). Molecular profiling stratifies diverse phenotypes of treatment-refractory metastatic castration-resistant prostate cancer. J Clin Invest.

[30] Nouruzi S (2022). ASCL1 activates neuronal stem cell-like lineage programming through remodeling of the chromatin landscape in prostate cancer. Nat Commun.

[31] Cejas P (2021). Subtype heterogeneity and epigenetic convergence in neuroendocrine prostate cancer. Nat Commun.

[32] Beltran H (2011). Molecular characterization of neuroendocrine prostate cancer and identification of new drug targets. Cancer Discov.

[33] Chandra D (2005). Bax-dependent regulation of Bak by voltage-dependent anion channel 2. J Biol Chem.

[34] Weeden CE (2018). Dual inhibition of BCL-XL and MCL-1 is required to induce tumour regression in lung squamous cell carcinomas sensitive to FGFR inhibition. Oncogene.

[35] Mason KD (2007). Programmed anuclear cell death delimits platelet life span. Cell.

[36] Perimbeti S (2023). Phase Ib trial of enzalutamide (Enza) with venetoclax (Ven) in metastatic castration-resistant prostate cancer (mCRPC). J Clin Oncol.

[37] Kolar Z (2000). Relation of Bcl-2 expression to androgen receptor, p21WAF1/CIP1, and cyclin D1 status in prostate cancer. Mol Pathol.

[38] Deng S (2022). Ectopic JAK-STAT activation enables the transition to a stem-like and multilineage state conferring AR-targeted therapy resistance. Nat Cancer.

[39] Pal SK (2018). Identification of mechanisms of resistance to treatment with abiraterone acetate or enzalutamide in patients with castration-resistant prostate cancer (CRPC). Cancer.

[40] He MX (2021). Transcriptional mediators of treatment resistance in lethal prostate cancer. Nat Med.

[41] Alumkal JJ (2020). Transcriptional profiling identifies an androgen receptor activity-low, stemness program associated with enzalutamide resistance. Proc Natl Acad Sci U S A.

[42] Chan JM (2022). Lineage plasticity in prostate cancer depends on JAK/STAT inflammatory signaling. Science.

[43] Soundararajan R (2018). EMT, stemness and tumor plasticity in aggressive variant neuroendocrine prostate cancers. Biochim Biophys Acta Rev Cancer.

[44] Paranjape AN (2016). Inhibition of FOXC2 restores epithelial phenotype and drug sensitivity in prostate cancer cells with stem-cell properties. Oncogene.

[45] Battula VL (2010). Epithelial-mesenchymal transition-derived cells exhibit multilineage differentiation potential similar to mesenchymal stem cells. Stem Cells.

[46] Augustyn A (2014). ASCL1 is a lineage oncogene providing therapeutic targets for high-grade neuroendocrine lung cancers. Proc Natl Acad Sci U S A.

[47] Pilling AB, Hwang C (2019). Targeting prosurvival BCL2 signaling through Akt blockade sensitizes castration-resistant prostate cancer cells to enzalutamide. Prostate.

[48] Sastry KS (2006). Epidermal growth factor protects prostate cancer cells from apoptosis by inducing BAD phosphorylation via redundant signaling pathways. J Biol Chem.

[49] Ren W (2016). Synthetic lethality in PTEN-mutant prostate cancer is induced by combinatorial PI3K/Akt and BCL-XL inhibition. Mol Cancer Res.

[50] Weston CR (2003). Activation of ERK1/2 by deltaRaf-1:ER* represses Bim expression independently of the JNK or PI3K pathways. Oncogene.

[51] Welti J (2016). Analytical validation and clinical qualification of a new immunohistochemical assay for androgen receptor splice variant-7 protein expression in metastatic castration-resistant prostate cancer. Eur Urol.

[52] Welti J (2021). Targeting the p300/CBP axis in lethal prostate cancer. Cancer Discov.

[53] Welti J (2018). Targeting bromodomain and extra-terminal (BET) family proteins in castration-resistant prostate cancer (CRPC). Clin Cancer Res.

[54] Gil V (2021). HER3 is an actionable target in advanced prostate cancer. Cancer Res.

[55] Nguyen HM (2017). LuCaP prostate cancer patient-derived xenografts reflect the molecular heterogeneity of advanced disease and serve as models for evaluating cancer therapeutics. Prostate.

[56] Pidsley R (2016). Critical evaluation of the Illumina MethylationEPIC BeadChip microarray for whole-genome DNA methylation profiling. Genome Biol.

[57] Quigley DA (2018). Genomic hallmarks and structural variation in metastatic prostate cancer. Cell.

[58] Zhao SG (2020). The DNA methylation landscape of advanced prostate cancer. Nat Genet.

