# Supplementary material for: BCL2 expression is enriched in advanced prostate cancer with features of lineage plasticity
Source: J Clin Invest. 2024 Sep 17;134(18):e179998. doi: 10.1172/JCI179998 (PMC11405043; doi:10.1172/JCI179998)
Supplement: Supplemental data [file jci-134-179998-s171.pdf]

Supplementary figure 1

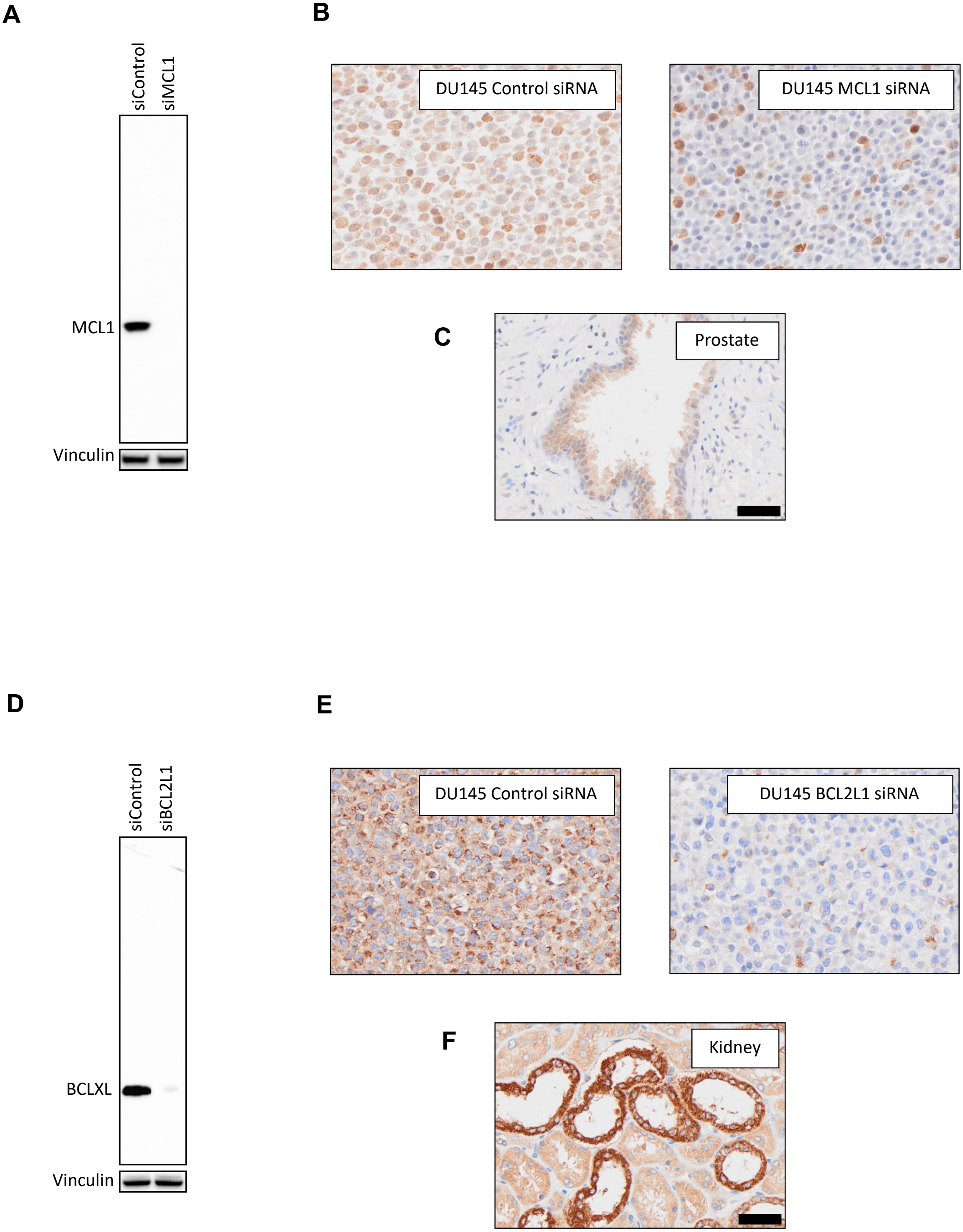

Supplementary Figure 1: MCL1 and BCLXL immunohistochemistry validation. **(A-B)** Western blot **(A)** and immunohistochemistry **(B)** of MCL1 expression in DU145 cells transfected with either non-targeting control or MCL1 siRNA (50 nM for 72 hours). **(C)** Human prostate tissue was used as a control for MCL1 expression. Scale bar, 50 µm. **(D-F)** Western blot **(D)** and immunohistochemistry **(E)** of BCLXL expression in DU145 cells transfected with either non-targeting control or BCL2L1 siRNA (50 nM for 72 hours). **(F)** Human kidney tissue was used as a control for BCLXL expression. Scale bar, 50 µm.

Supplementary figure 2

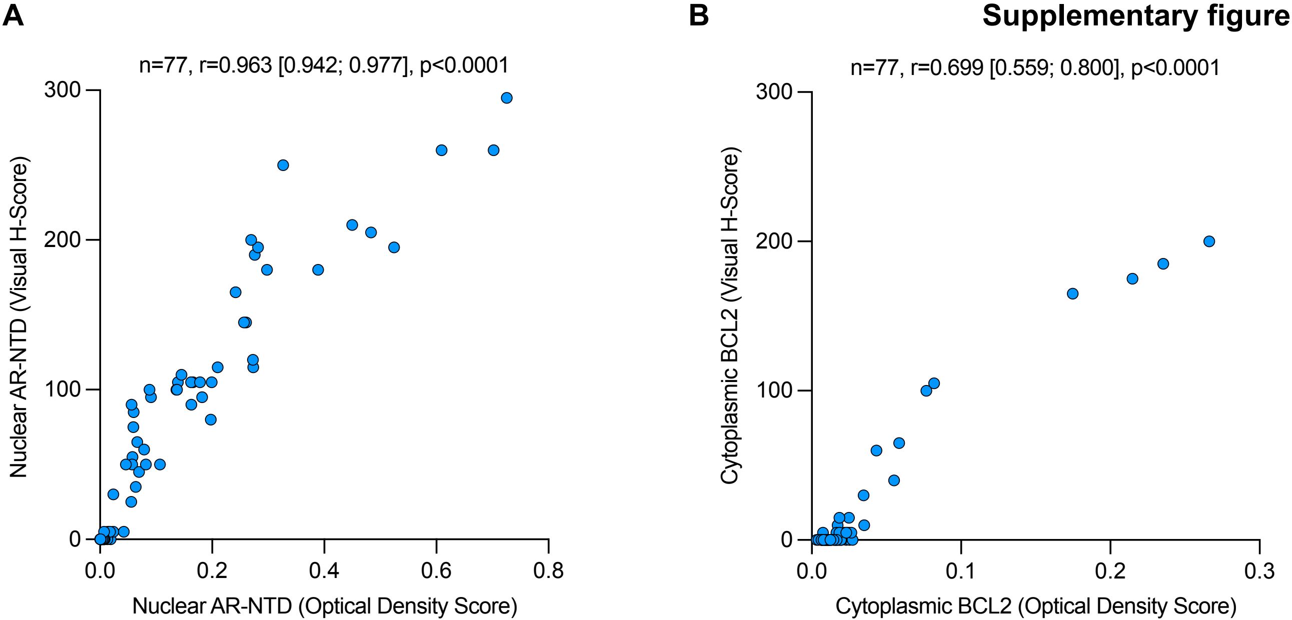

Supplementary Figure 2: Association between different IHC quantification methods. (A-B) Immunohistochemistry (IHC) quantification for **(A)** nuclear AR-NTD and **(B)** cytoplasmic BCL2 was determined using both the automated optical density (OD) score and the visual modified H-score in a subset of UW/FHCC patient and LuCaP PDX tissue microarray samples (n=77). Spearman's correlation was used for statistical analysis.

Supplementary figure 3

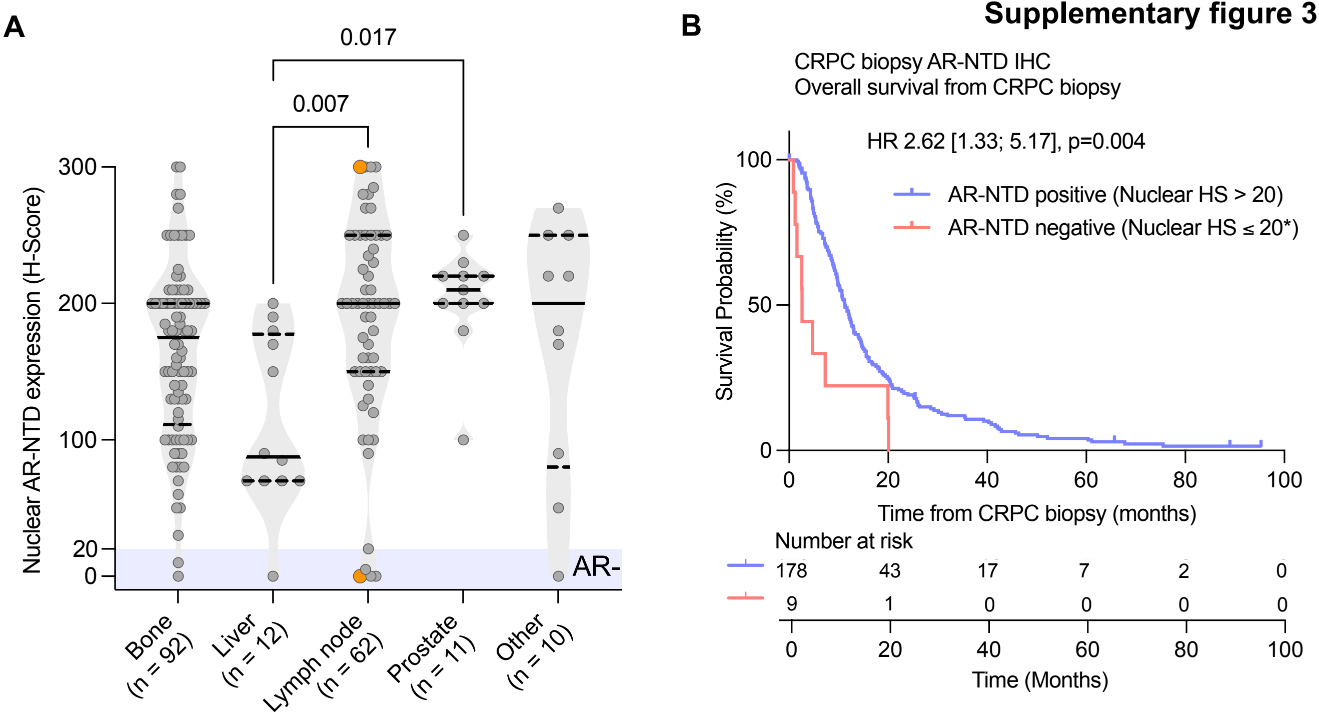

Supplementary Figure 3: Nuclear AR-NTD expression by site of biopsy and overall survival from CRPC biopsy split by AR-NTD status. (A) Nuclear AR-NTD expression (H-score) by site of CRPC biopsy for the ICR/RMH cohort (n=187). Medians and interquartile ranges (IQRs) are shown. Tukey's multiple comparison test was used to determine statistical significance. The heterogenous case with two tumour cell populations (AR positive and AR negative) is highlighted in orange and included twice. **(B)** Kaplan-Meier overall survival curves from time of CRPC biopsy, split by AR positive (H-score > 20) and AR negative (H-score ≤ 20) tumours. Hazard ratio (HR) with 95% confidence intervals and p-value for log-rank test are shown. *The heterogenous case is included in the AR-NTD negative group. HS = H-score.

Supplementary figure 4

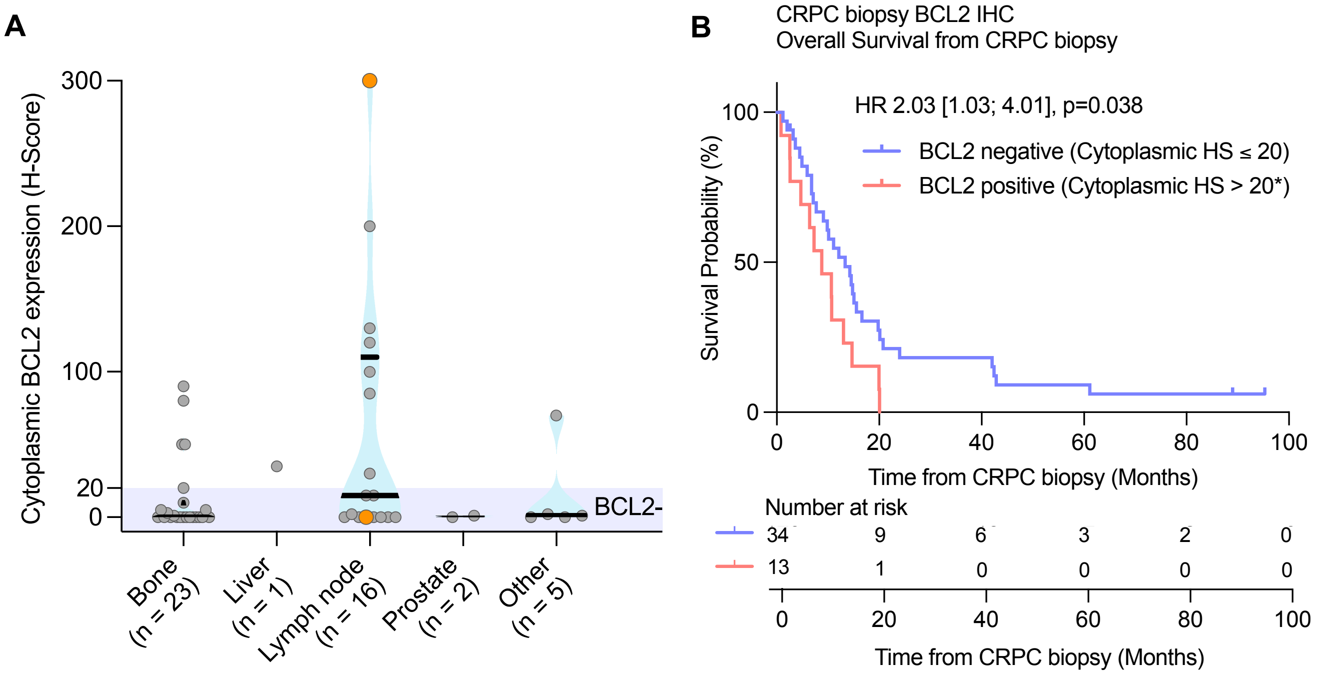

Supplementary Figure 4: Cytoplasmic BCL2 expression by site of biopsy and overall survival from CRPC biopsy split by BCL2 status. (A) Cytoplasmic BCL2 expression (H-score) by site of CRPC biopsy for the ICR/RMH cohort (n=47). Medians and IQRs are shown. Tukey's multiple comparison test was used to determine statistical significance. The heterogenous case with two tumour cell populations (BCL2 positive and BCL2 negative) is highlighted in orange and included twice. **(B)** Kaplan-Meier overall survival curves from time of CRPC biopsy, split by BCL2 positive (H-score > 20) and BCL2 negative (H-score ≤ 20) tumours. Hazard ratio (HR) with 95% confidence intervals and p-value for log-rank test are shown. *The heterogenous case is included in the BCL2 positive group. HS = H-score.

Supplementary figure 5

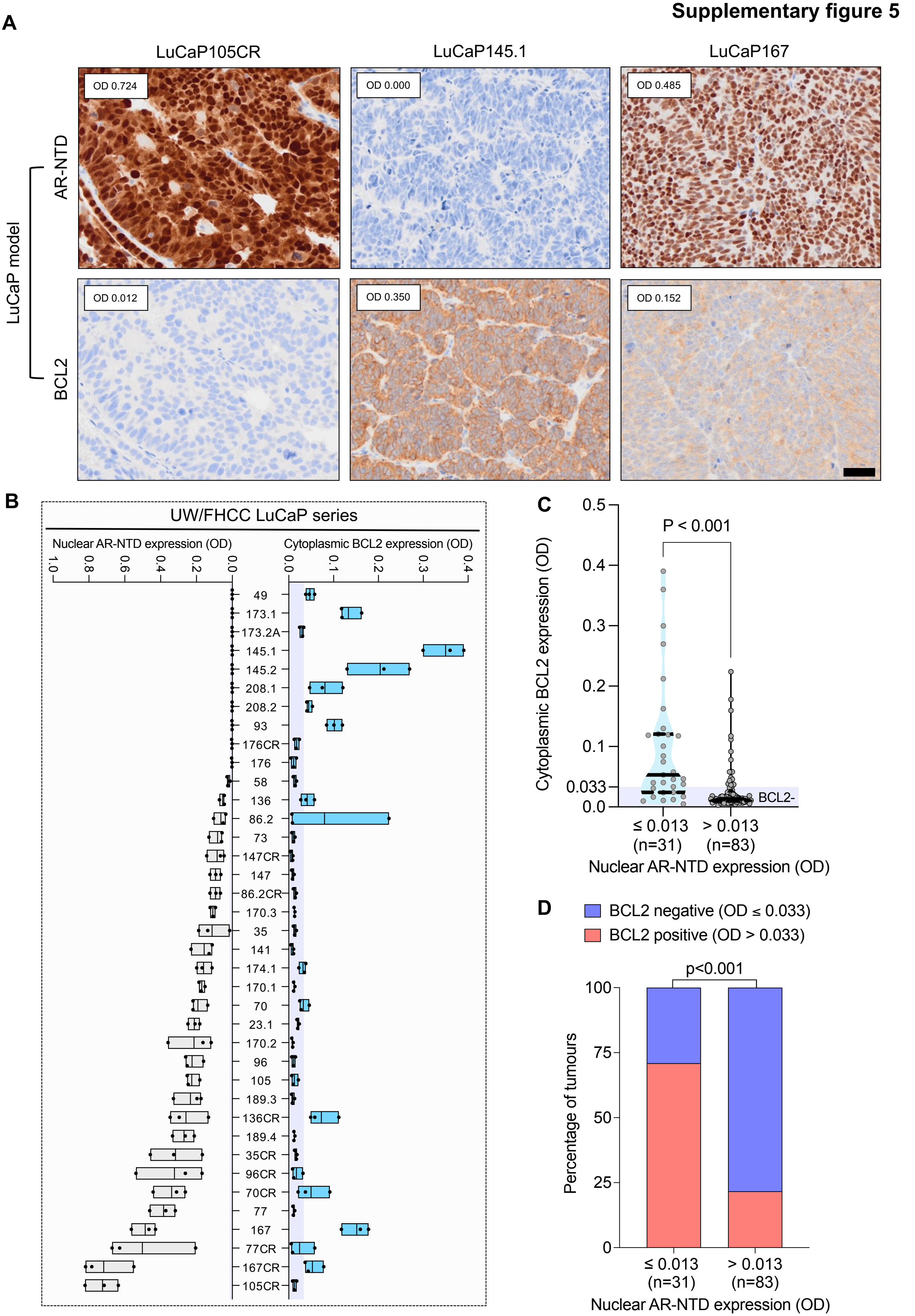

Supplementary Figure 5: BCL2 protein expression is enriched in LuCaP patient-derived xenograft CRPC models with AR protein loss. (A-D) IHC for nuclear AR-NTD and cytoplasmic BCL2 was performed on 38 PDX CRPC models (3 samples from 3 different passages were analysed for each model). The castrated CRPC models are denoted 'CR'. **(A)** Representative micrographs for AR-NTD and BCL2 are shown, with examples of AR-NTD positive and BCL2 negative (LuCaP105CR), AR-NTD negative and BCL2 positive (LuCaP145.1), and AR-NTD and BCL2 positive (LuCaP167). Scale bar, 50 µm. OD = Optical density. **(B)** Optical density scores (OD) for AR-NTD and BCL2. Bars show the mean and range. The OD thresholds for AR (≤ 0.013) and BCL2 (≤ 0.033) negativity are highlighted blue. **(C)** Cytoplasmic BCL2 expression (OD) is shown for CRPC biopsies with AR negative (AR-NTD OD ≤ 0.013, n=31) and AR positive (AR-NTD OD > 0.013, n=83) staining. Medians and IQRs are shown. The Mann-Whitney U test was used to determine statistical significance. The threshold for BCL2 negativity (OD ≤ 0.033) is highlighted blue. **(D)** The percentage of BCL2 positive (OD > 0.033) and BCL2 negative (OD ≤ 0.033) tumours split by AR expression status as above. The Fisher's exact test was used to determine the statistical significance.

Supplementary figure 6

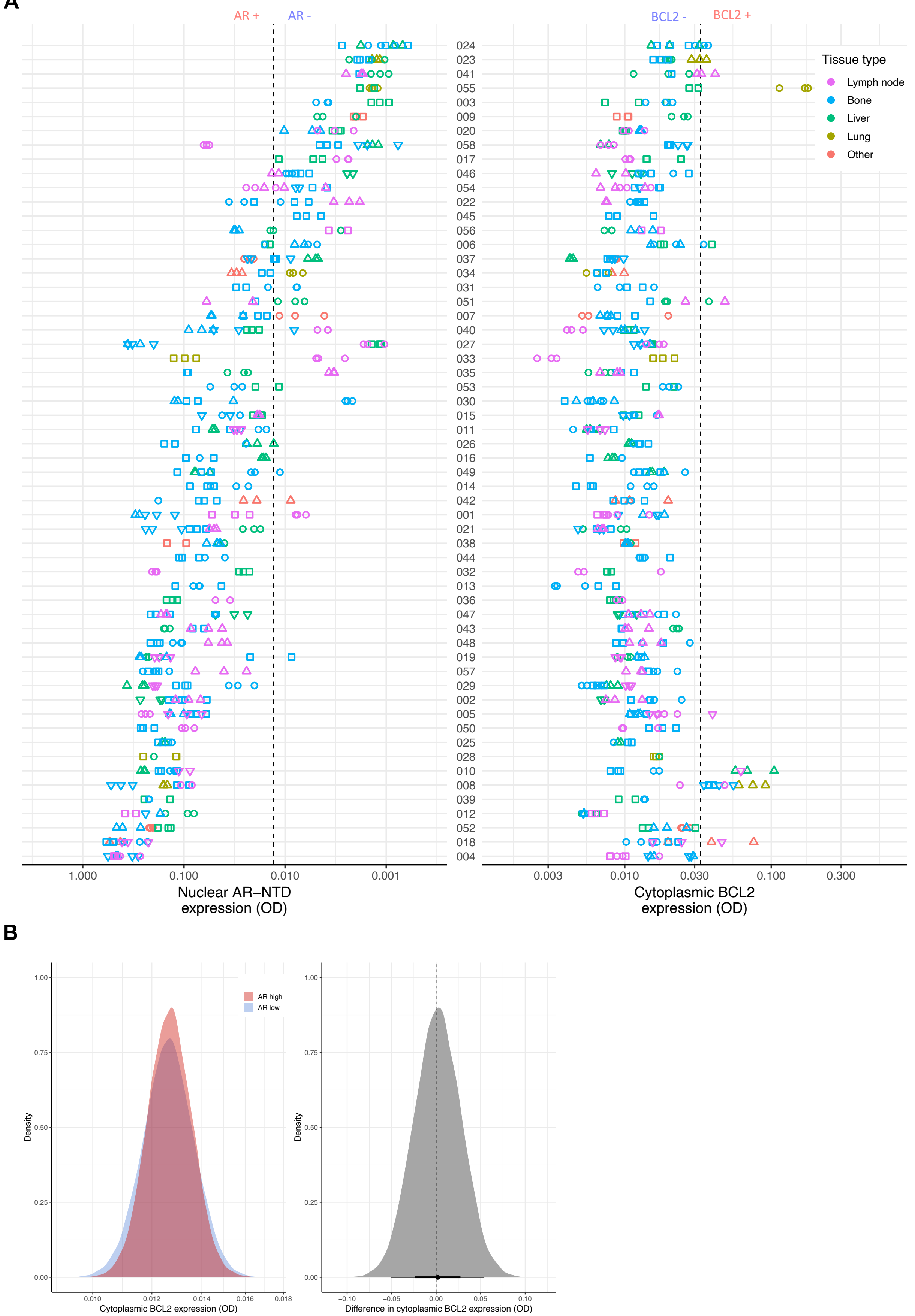

Supplementary Figure 6: AR-NTD and BCL2 expression in the UW/FHCC CRPC IHC cohort. AR-NTD and BCL2 IHC was performed on 485 spatially separated samples in 177 CRPC biopsies taken from 58 patients at rapid autopsy. **(A)** Nuclear AR-NTD and cytoplasmic BCL2 optical density (OD) scores for all samples are shown by patient. Colours indicate tissue type, and each distinct shape within a patient represents a different metastatic site. Dotted lines show the OD thresholds for AR (≤ 0.013) and BCL2 (≤ 0.033) negativity. **(B)** Binary AR-NTD expression (OD >0.013) was added as a population-level effect to the BCL2 intercept-only model to assess the association between AR-NTD and BCL2 expression. Posterior distributions of BCL2 expression by AR-NTD expression (left plot) and of pairwise difference in BCL2 expression (right plot) between AR negative (OD ≤ 0.013) and AR positive (OD > 0.013) expressing groups are shown.

Supplementary figure 7

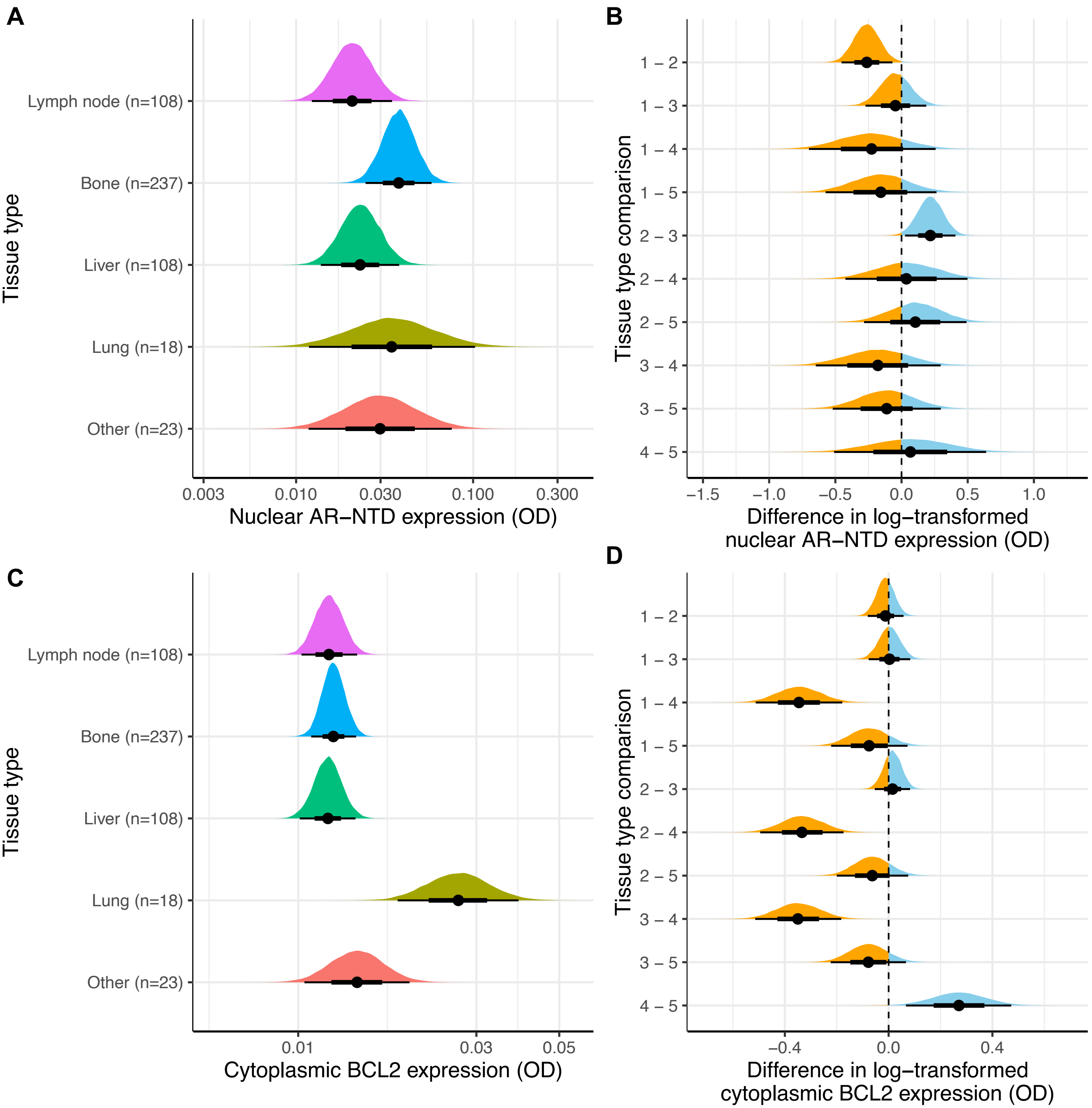

Supplementary Figure 7: AR-NTD and BCL2 expression in the UW/FHCC CRPC IHC cohort by site of metastatic disease. AR-NTD and BCL2 IHC was performed on 485 spatially separated samples in 177 CRPC biopsies taken from 58 patients at rapid autopsy. **(A-D)** Posterior distributions as estimated using the Bayesian hierarchical model with the posterior mean (dot), 95% credible intervals (thick line) and 66% credible intervals (thin line) being shown in each plot. **(A)** Posterior distribution of nuclear AR-NTD expression (OD) by metastatic tissue type. **(B)** Posterior distribution of the difference in log-transformed nuclear AR-NTD expression (OD) between tissue types. **(C)** Posterior distribution of cytoplasmic BCL2 expression (OD) by metastatic tissue type. **(D)** Posterior distribution of the difference in log-transformed cytoplasmic BCL2 expression (OD) between tissue types. 1=Lymph Node, 2=Bone, 3=Liver, 4=Lung, 5=Other.

Supplementary figure 8

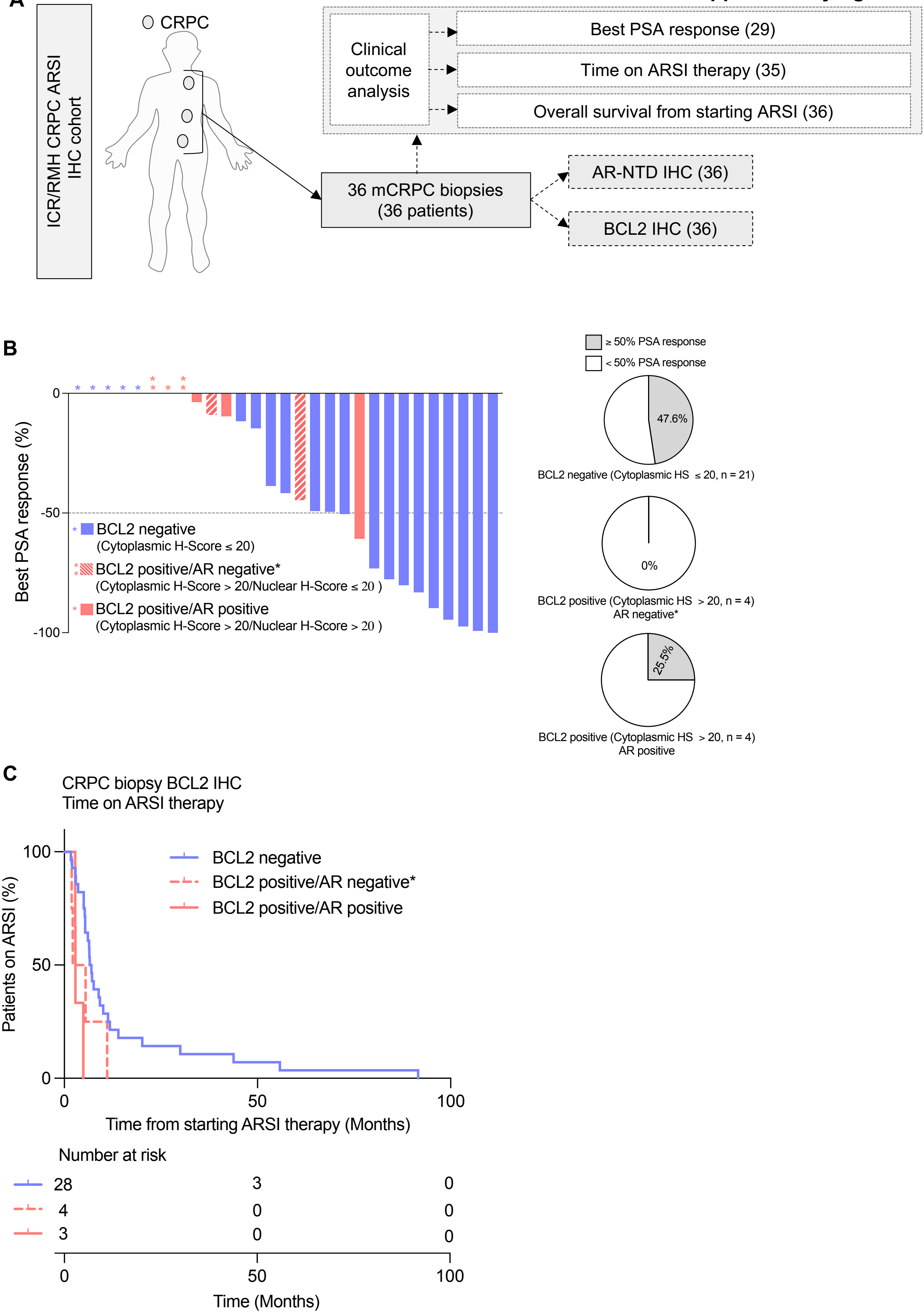

Supplementary Figure 8: BCL2 positive CRPC associates with resistance to therapies targeting the AR signalling axis, irrespective of AR expression status. (A) Summary of clinical samples analysed from the ICR/RMH ARSI IHC cohort. A total of 36 CRPC metastases from 36 patients were included, all of which had IHC for nuclear AR-NTD and cytoplasmic BCL2 expression. Clinical outcome data, including best PSA response (n=29), time on ARSI (n=35) and overall survival (n=36) from starting AR targeting therapy, were collected. **(B)** (Left panel) Waterfall plot of greatest percentage fall in PSA from baseline for 29 patients treated with either abiraterone or enzalutamide, split by BCL2 negative (blue), BCL2 positive/AR negative (red + white stripes) and BCL2 positive/AR positive (red). (Right panel) Pie charts showing percentage of patients from each group that had a ≥50% fall in PSA from baseline. * Indicates no response. **(C)** Kaplan-Meier curves, for each group described above, showing time on ARSI therapy. *The heterogenous case is included in the BCL2 positive/AR negative group. ARSI = Androgen receptor signalling inhibitor.

Supplementary figure 9

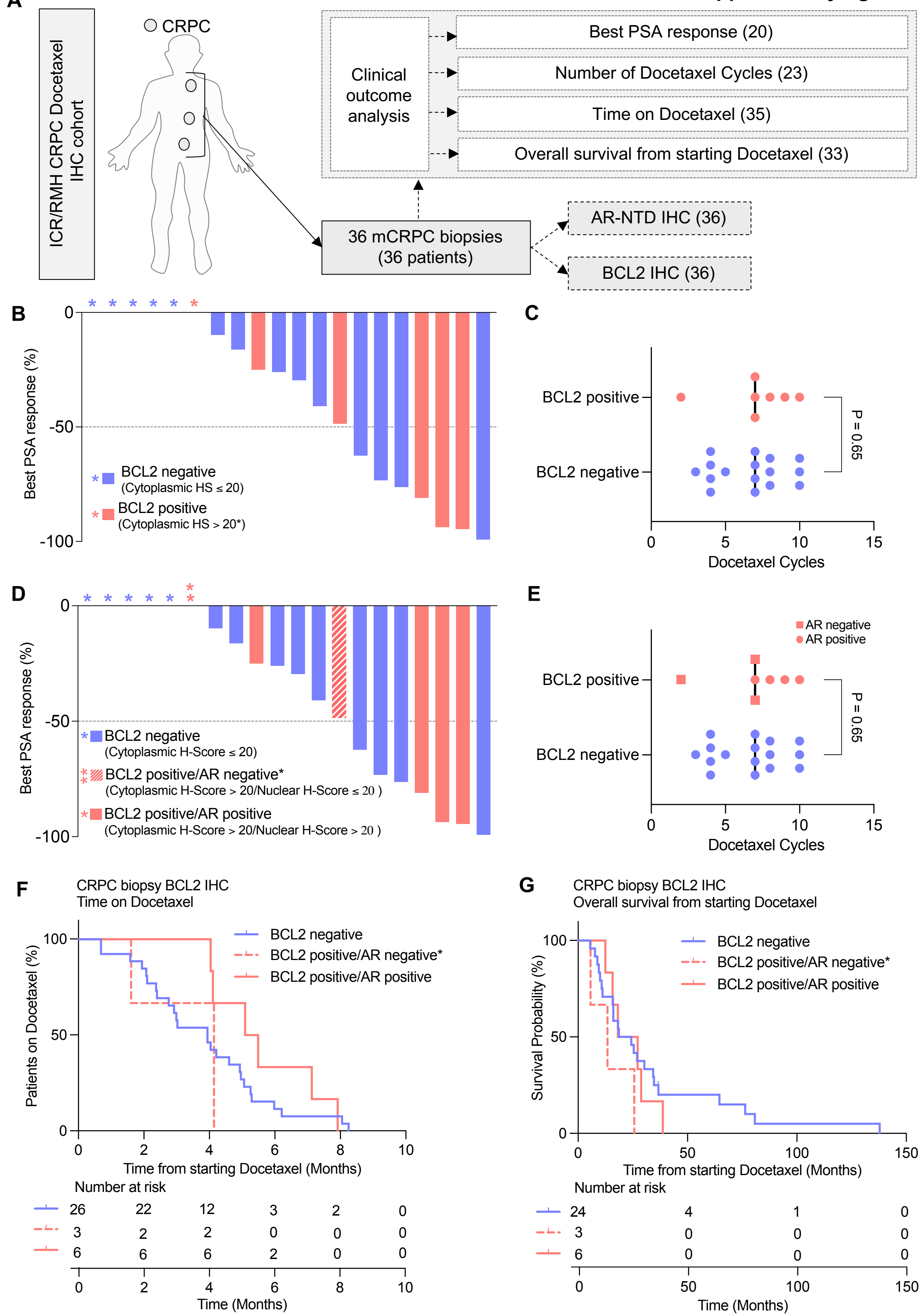

Supplementary Figure 9: BCL2 positive CRPC and docetaxel treatment. (A) Summary of clinical samples analysed from the ICR/RMH Docetaxel IHC cohort. A total of 36 CRPC metastases from 36 patients were included, all of which had IHC for nuclear AR-NTD and cytoplasmic BCL2 expression. Clinical outcome data, including best PSA response (n=20), number of docetaxel cycles (n=23), time on docetaxel (n=35) and overall survival (n=33) from starting docetaxel, were collected. **(B)** Waterfall plot of greatest percentage fall in PSA from baseline for 20 patients treated with docetaxel, split by BCL2 negative (blue) and BCL2 positive (red). * Indicates no response. **(C)** The number of docetaxel cycles received, split by patients (n=23) with BCL2 negative (blue) and BCL2 positive (red) tumours. The Mann-Whitney U test was used to determine statistical significance. **(D)** Waterfall plot of greatest percentage fall in PSA from baseline for patients treated with docetaxel, split by BCL2 negative (blue), BCL2 positive/AR negative (red + white stripes) and BCL2 positive/AR positive (red). * Indicates no response. **(E)** The number of docetaxel cycles received, split by patients with BCL2 negative (blue) and BCL2 positive (red) tumours. BCL2 positive tumours are identified as AR negative (red square) and AR positive (red circle). The Mann-Whitney U test was used to determine statistical significance. **(F-G)** Kaplan-Meier curves, split by BCL2 positive/AR negative, BCL2 positive/AR positive and BCL2 negative, for time on docetaxel **(F)** and overall survival from initiation of docetaxel **(G)**.

Supplementary figure 10

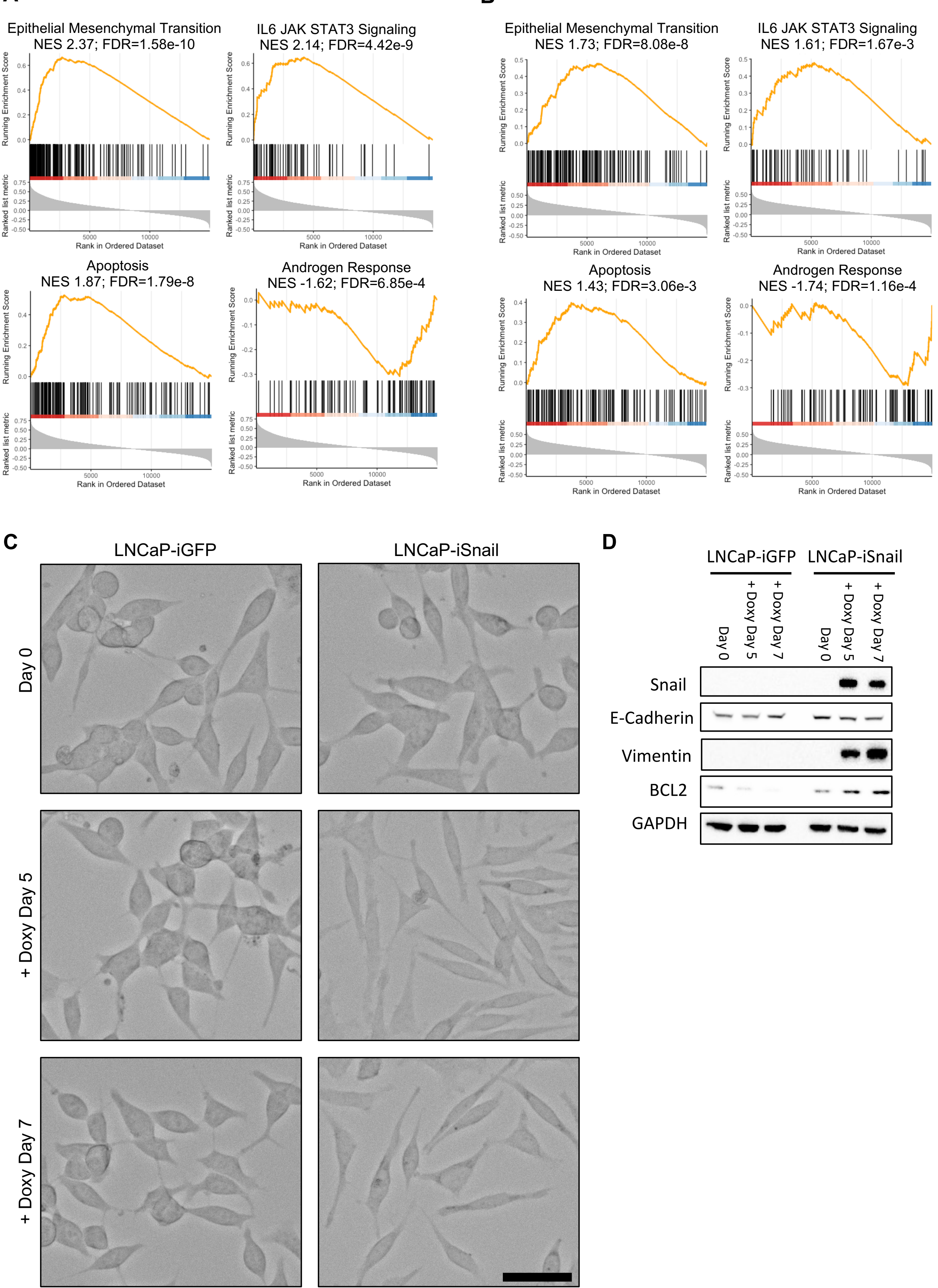

Supplementary Figure 10: *BCL2* expression associates with pathways implicated in lineage plasticity, including EMT, and driven by Snail overexpression. (A-B) Gene set enrichment analyses (GSEA) using the 'hallmark molecular signatures' was performed for *BCL2* mRNA expression in the **(A)** ICR/RMH (n=95) and **(B)** SU2C/PCF (n=159) CRPC RNA sequencing cohorts. Enrichment plots for selected signatures are shown. **(C)** Representative microscopy images of LNCaP-iGFP and LNCaP-iSnail cells at baseline (day 0) and after 5 and 7 days of doxycycline treatment. Scale bar 50 µm. **(D)** Western blot (2nd biological replicate) showing protein expression of Snail, E-cadherin and Vimentin in untreated (day 0) LNCaP-iGFP and LNCaP-iSnail, and cells treated for 5 and 7 days with doxycycline. GAPDH was used as a loading control.

Supplementary figure 11

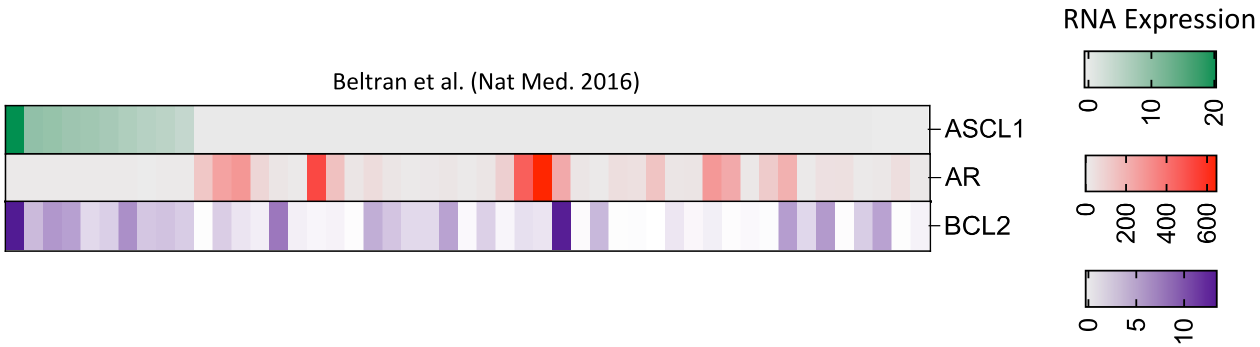

Supplementary Figure 11: *ASCL1* mRNA is exclusively expressed in AR negative castration-resistant prostate cancer, all of which concurrently express *BCL2* mRNA. Publicly available RNA sequencing data from a CRPC patient cohort enriched with neuroendocrine prostate cancer (Beltran et al., Nat Med. 2016) was downloaded from cBioPortal. Heatmap showing *ASCL1*, *AR* and *BCL2* mRNA expression in 49 patient biopsies. The tumours are ordered by *ASCL1* expression.

Supplementary figure 12

A

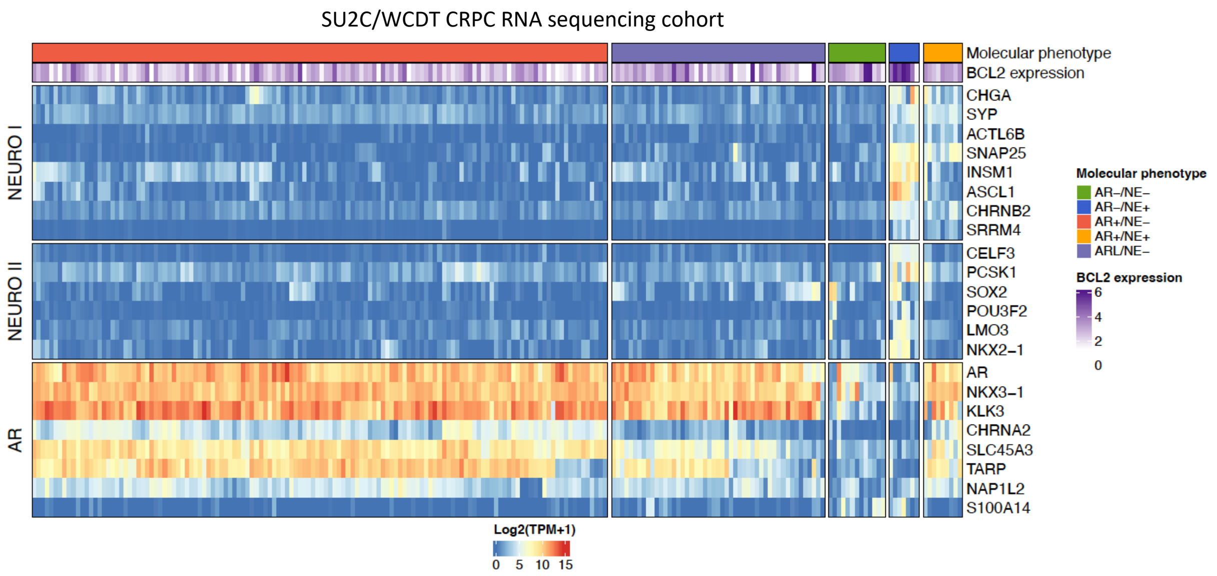

B

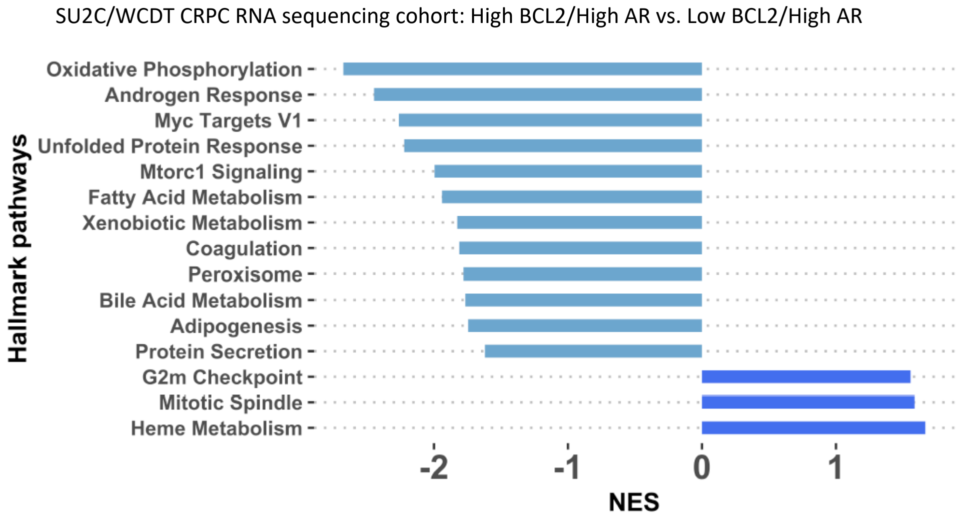

Supplementary Figure 12: Gene set enrichment analyses (GSEA) comparing tumours with high BCL2/high AR vs. low BCL2/high AR. (A) Heatmap depicting *BCL2* mRNA expression and in the expanded SU2C/WCDT mCRPC cohort (n=210). Tumours are grouped by molecular phenotype as determined using the AR, NEURO I and NEURO II gene expression sets. ARL = AR low. **(B)** GSEA using the ‘hallmark molecular signatures’ was undertaken for samples with high BCL2/high AR (n=6) vs. low BCL2/high AR (n=99) from the SU2C/WCDT CRPC RNA sequencing cohort. Pathways enriched with FDR < 0.05 are shown. BCL2 cut off: ≥ 90th percentile expression, AR cut off: median expression. NES = normalized enrichment score; FDR = false discovery rate.

Supplementary figure 13

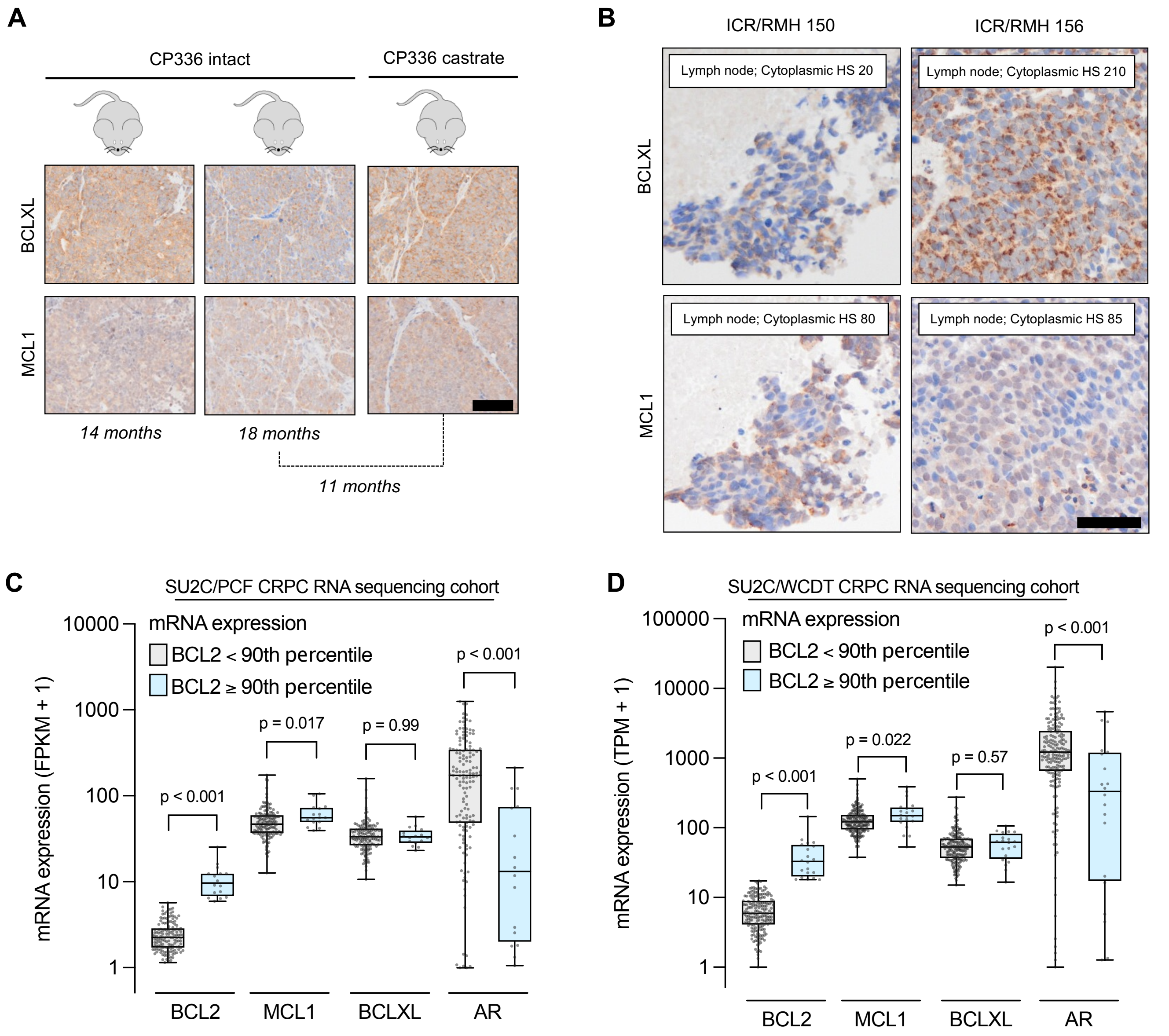

Supplementary Figure 13: Expression of anti-apoptotic proteins, BCLXL and MCL1, in BCL2 positive CRPC. (A) CP336 intact was passaged into castrate mice to develop CP336 castrate (CP336c). IHC for BCLXL and MCL1 was performed at different timepoints as shown. Scale bar, 100 µm **(B)** IHC for BCLXL and MCL1 was performed on two BCL2 positive CRPC biopsies. Scale bar, 50 µm. HS = H-score. **(C-D)** Transcriptome analyses associating *BCL2* mRNA expression (<90th percentile vs ≥90th percentile) with *MCL1*, *BCLXL* and *AR* mRNA expression in the SU2C/PCF (n=159) **(C)** and SU2C/WCDT (n=210) **(D)** CRPC RNA sequencing cohorts. Medians and IQRs are shown. The Mann Whitney-U test was used to determine the statistical significance.

Supplementary figure 14

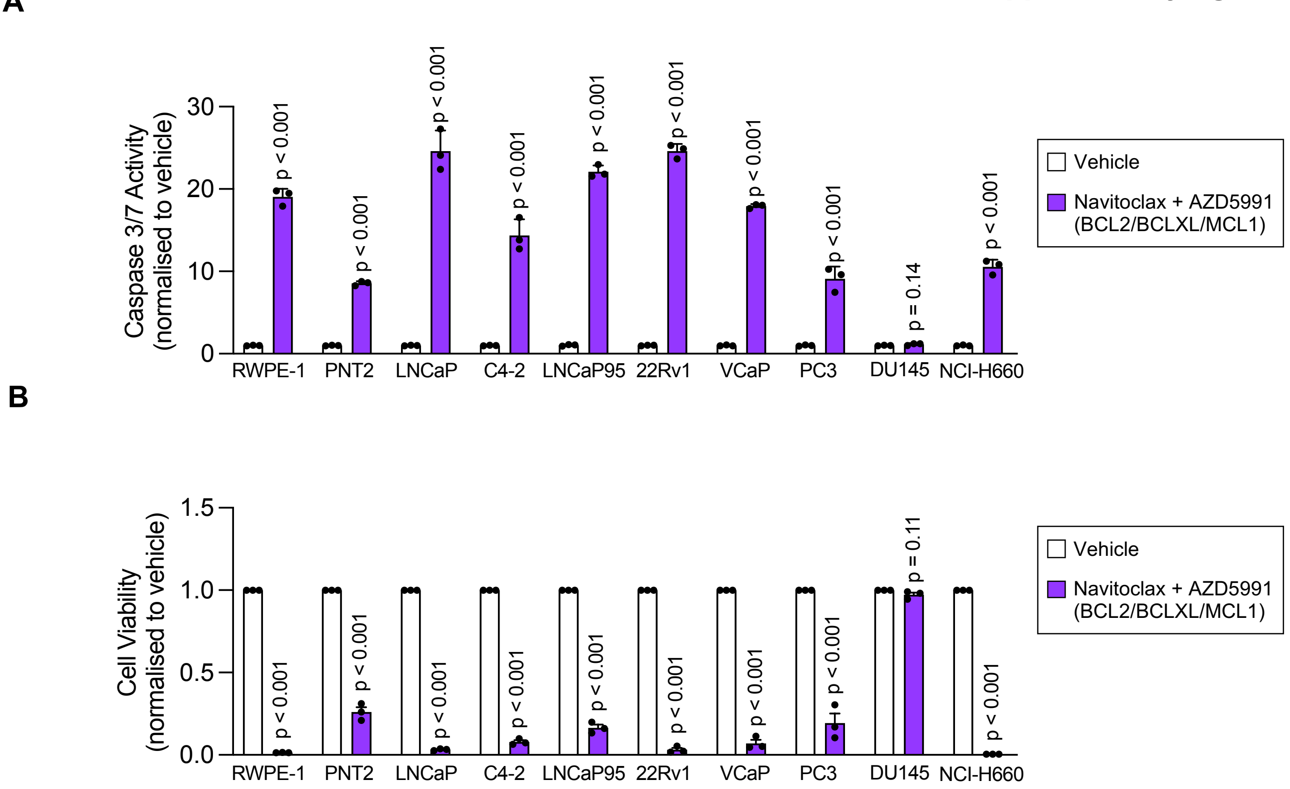

Supplementary Figure 14: Contemporaneously targeting BCL2, BCLXL and MCL1 in prostate cell lines. (A-B) The impact of navitoclax (1 µM, BCL2/BCLXL inhibitor) and AZD5991 (1 µM, MCL1 inhibitor) on caspase 3/7 activity (6 hours, Caspase 3/7-Glo) **(A)** and cell viability (24 hours, CellTiter-Glo) **(B)** was determined in 10 prostate cell lines (RWPE1 and PNT2 = benign, LNCaP, C4-2, LNCaP95, 22Rv1, VCaP, PC3, DU145, NCI-H660). Caspase 3/7 analysis was undertaken in a single experiment with 3 technical replicates (mean and standard deviation are shown). Cell viability analysis was performed in biological triplicate with 3 technical replicates (mean and standard error of mean are shown). The unpaired t test was used to determine the statistical significance of the impact of navitoclax and AZD5991 compared to the vehicle in each cell line.

Clinical Characteristics (AR-NTD IHC; ICR/RMH Cohort)	
**Archival (castration-sensitive) biopsy (n=60)**	
**Histology (n, %)**	
Adenocarcinoma	60, 100%
**Site and collection technique**	
Prostate needle biopsy	48, 80%
Prostatectomy	6, 10%
Transurethral resection of the prostate	5, 8%
Bone biopsy	1, 2%
**Gleason score (n, %)**	
<7	4, 7%
7	16, 27%
8	14, 23%
9	16, 27%
10	8, 13%
NR	2, 3%
**Treatment intent at diagnosis (n, %)**	
Radical	26, 43%
Palliative	34, 57%
**Age at diagnosis (median, range)**	63, 39-75
**Time (months) to CRPC (median, range)**	25, 4-163
**Metastatic (castration-resistant) biopsy (n=187; 60 paired)**	
**Biopsy site (n, %)**	
Bone	92, 49%
Lymph node	62, 33%
Liver	12, 6%
Prostate	11, 6%
Other	10, 5%
**Treatments prior to biopsy**	
Taxane	143, 76%
AR-signalling inhibitor (2nd generation)	134, 72%
Radium-223	13, 7%
Carboplatin	4, 2%
**Age at CRPC biopsy (median, range)**	68, 39-89

Supplementary Table 1: Clinical characteristics (ICR/RMH IHC cohort) for cases analysed with AR-NTD IHC.IHC = immunohistochemistry; CRPC = castration-resistant prostate cancer.

Clinical Characteristics (AR-NTD + BCL2 IHC; ICR/RMH cohort)	
**Metastatic (castration-resistant) biopsy (n=47)**	
**Biopsy site (n, %)**	
Bone	23, 49%
Lymph node	16, 34%
Liver	1, 2%
Prostate	2, 4%
Other	5, 11%
**Treatments prior to biopsy**	
Taxane	28, 60%
AR-signalling inhibitor (2nd generation)	31, 66%
Radium-223	2, 4%
Carboplatin	2, 4%
**Age at CRPC biopsy (median, range)**	68, 39-82

Supplementary Table 2: Clinical characteristics (ICR/RMH IHC cohort) for cases analysed with BCL2 IHC. IHC = immunohistochemistry; CRPC = castration-resistant prostate cancer.

Exposure to taxane chemotherapy before ARSI in the ICR/RMH CRPC ARSI IHC cohort			
**Group**	**Outcome Measure**		
	**Time on treatment**	**OS**	**PSA response**
BCL2 negative	12/28, 43%	12/28, 43%	6/21, 29%
BCL2 positive	4/7, 57%	5/8, 63%	5/8, 63%
BCL2 positive/ AR negative	1/4, 25%	1/4, 25%	1/4, 25%
BCL2 positive/ AR positive	3/3, 100%	4/4, 100%	4/4%, 100%

Supplementary Table 3: Exposure to taxane chemotherapy (Docetaxel/Cabazitaxel) before ARSI in the ICR/RMH CRPC ARSI IHC cohort. ARSI = Androgen receptor signalling inhibitor; OS = overall survival; PSA = prostate-specific antigen.

Exposure to ARSI before Docetaxel in the ICR/RMH CRPC Docetaxel IHC cohort				
Group	Outcome Measure			
	Time on treatment	OS	PSA response	Number of cycles
BCL2 negative	13/26, 50%	12/24, 50%	9/14, 64%	9/16, 56%
BCL2 positive	3/9, 33%	3/9, 33%	1/6, 17%	2/7, 29%
BCL2 positive/ AR negative	2/3, 66%	2/3, 66%	1/2, 50%	2/3, 66%
BCL2 positive/ AR positive	1/6, 17%	1/6, 17%	0/4, 0%	0/4, 0%

Supplementary Table 4: Exposure to ARSI before docetaxel in the ICR/RMH CRPC Docetaxel IHC cohort. ARSI = ARSI = Androgen receptor signalling inhibitor; OS = overall survival; PSA = prostate-specific antigen.

Protein Target	Supplier	Catalogue No.	Species
BCL2	Dako	M0887	Mouse monoclonal
Snail	Cell Signaling	3879	Rabbit monoclonal
E-cadherin	Cell Signaling	14472	Mouse monoclonal
Vimentin	Cell Signaling	5741	Rabbit monoclonal
AR-NTD	Dako	M3562	Mouse monoclonal
ASCL1	Abcam	Ab211327	Rabbit monoclonal
Vinculin	Santa Cruz Biotechnology	sc-73614	Mouse monoclonal
GAPDH	Santa Cruz Biotechnology	sc-32233	Mouse monoclonal
MCL1	Proteintech	16225-1-AP	Rabbit polyclonal
BCLXL	Cell Signaling	2764	Rabbit monoclonal

Supplementary Table 5: Primary antibodies used for Western Blotting

Cell Line	Supplier	Catalogue No.	Media	Serum	Supplements
PNT2	Sigma-Aldrich	95012613	RPMI	FBS	L-Glutamine
RWPE-1	ATCC	CRL-3607	Keratinocyte	NA	L-Glutamine BPE/EGF
LNCaP	ATCC	CRL-1740	RPMI	FBS	L-Glutamine
C4-2	ATCC	CRL-3314	DMEM	FBS	L-Glutamine
LNCaP95	Dr Meeker & Dr Luo*	NA	RPMI (phenol red free)	CSS	L-Glutamine
22Rv1	ATCC	CRL-2505	RPMI	FBS	L-Glutamine
VCaP	ATCC	CRL-2876	DMEM	FBS	L-Glutamine
PC3	ATCC	CRL-1345	RPMI	FBS	L-Glutamine
DU145	ATCC	HTB-81	RPMI	FBS	L-Glutamine
NCI-H660	Prof Bevan**	CRL-5813	RPMI	FBS	L-Glutamine, Insulin, Transferrin, Selenium, Hydrocortisone, Beta-estradiol
LNCaP-iGFP LNCaP-iSnail	Dr Hollier***	NA	RPMI	FBS Tet-system approved	L-Glutamine

Supplementary Table 6: Cell Lines

ATCC: American Type Culture Collection, FBS: fetal bovine serum, CSS: charcoal stripped serum, *: gifted from Drs. Alan K Meeker and Jun Luo (Johns Hopkins University, Baltimore, Maryland, USA), ** gifted from Prof. Charlotte Bevan (Imperial College, London, UK, SW7 2AZ). *** gifted from Dr Brett Hollier (Australian Prostate Cancer Research Centre, Queensland, Brisbane, Australia). BPE/EGF: bovine pituitary extract and human recombinant epidermal growth factor. NA: non-applicable

	Estimate	CI.Lower	CI.Upper
Intra-cluster correlation (between patients)	0.644	0.517	0.756
Intra-cluster correlation (between metastatic sites)	0.324	0.219	0.445
Proportion of unexplained residual variance (within metastatic sites)	0.032	0.043	0.022

Supplementary Table 7: Intra-cluster correlation for nuclear AR-NTD expression in UW/FHCC CRPC IHC cohort. Intra-cluster correlation (for the patient and metastatic site level) was calculated as the ratio of the between-cluster variance to the total variance. 95% credible interval (CI) limits are shown.

	Estimate	CI.Lower	CI.Upper
Intra-cluster correlation (between patients)	0.528	0.392	0.656
Intra-cluster correlation (between metastatic sites)	0.313	0.214	0.427
Proportion of unexplained residual variance (within metastatic sites)	0.159	0.208	0.116

Supplementary Table 8: Intra-cluster correlation for cytoplasmic BCL2 expression in UW/FHCC CRPC IHC cohort. AR-NTD and BCL2 IHC was performed on 485 spatially separated samples in 177 CRPC biopsies taken from 58 patients at rapid autopsy. Intra-cluster correlation (for the patient and metastatic site level) was calculated as the ratio of the between-cluster variance to the total variance. 95% credible interval (CI) limits are shown.

Difference in log-transformed nuclear AR-NTD expression (OD)	Estimate	CI Lower	CI Upper	Pr(A>B)
Lymph node (A) vs Bone (B)	-0.231	-0.378	-0.082	0.007
Lymph node (A) vs Liver (B)	0.019	-0.157	0.195	0.568
Lymph node (A) vs Lung (B)	-0.080	-0.451	0.289	0.360
Lymph node (A) vs Other (B)	-0.112	-0.431	0.212	0.282
Bone (A) vs Liver (B)	0.250	0.104	0.397	0.997
Bone (A) vs Lung (B)	0.151	-0.208	0.510	0.757
Bone (A) vs Other (B)	0.119	-0.180	0.421	0.741
Liver (A) vs Lung (B)	-0.099	-0.461	0.264	0.329
Liver (A) vs Other (B)	-0.131	-0.440	0.182	0.249
Lung (A) vs Other (B)	-0.032	-0.473	0.414	0.450

Supplementary Table 9: Pairwise differences for log-transformed nuclear AR-NTD expression between metastatic tissue types in UW/FHCC CRPC IHC cohort. AR-NTD and BCL2 IHC was performed on 485 spatially separated samples in 177 CRPC biopsies taken from 58 patients at rapid autopsy. 95% credible interval (CI) limits and posterior probabilities (Pr) for nuclear AR-NTD expression (OD) in tissue type (A) being greater than in tissue type (B) are shown. OD = optical density.

Difference in log-transformed cytoplasmic BCL2 expression (OD)	Estimate	CI Lower	CI Upper	Pr(A>B)
Lymph node (A) vs Bone (B)	-0.039	-0.092	0.014	0.111
Lymph node (A) vs Liver (B)	-0.021	-0.083	0.041	0.286
Lymph node (A) vs Lung (B)	-0.351	-0.481	-0.220	0.000
Lymph node (A) vs Other (B)	-0.101	-0.215	0.014	0.073
Bone (A) vs Liver (B)	0.018	-0.034	0.070	0.721
Bone (A) vs Lung (B)	-0.311	-0.438	-0.185	0.000
Bone (A) vs Other (B)	-0.062	-0.167	0.044	0.167
Liver (A) vs Lung (B)	-0.330	-0.459	-0.202	0.000
Liver (A) vs Other (B)	-0.080	-0.190	0.031	0.115
Lung (A) vs Other (B)	0.250	0.093	0.407	0.996

Supplementary Table 10: Pairwise differences for log-transformed cytoplasmic BCL2 expression between metastatic tissue types in UW/FHCC CRPC IHC cohort. AR-NTD and BCL2 IHC was performed on 485 spatially separated samples in 177 CRPC biopsies taken from 58 patients at rapid autopsy. 95% credible interval (CI) limits and posterior probabilities (Pr) for cytoplasmic BCL2 expression (OD) in tissue type (A) being greater than in tissue type (B) are shown. OD = optical density.

Gene set enrichment analysis for *BCL2* mRNA expression (ICR/RMH RNA sequencing cohort)		
**Pathway**	**NES**	**FDR**
ALLOGRAFT REJECTION	2.41	1.58E-10
INTERFERON GAMMA RESPONSE	2.41	1.58E-10
EPITHELIAL MESENCHYMAL TRANSITION	2.37	1.58E-10
INFLAMMATORY RESPONSE	2.31	1.58E-10
INTERFERON ALPHA RESPONSE	2.21	1.58E-10
MYOGENESIS	2.18	1.58E-10
COMPLEMENT	2.15	1.58E-10
TNFA SIGNALING VIA NFKB	2.15	1.58E-10
IL6 JAK STAT3 SIGNALING	2.14	4.42E-09
APICAL JUNCTION	2.03	1.58E-10
KRAS SIGNALING UP	2.01	1.58E-10
UV RESPONSE DN	1.88	2.21E-08
COAGULATION	1.87	6.79E-07
APOPTOSIS	1.87	1.79E-08
TGF BETA SIGNALING	1.8	1.78E-04
IL2 STAT5 SIGNALING	1.75	1.15E-06
APICAL SURFACE	1.69	2.54E-03
P53 PATHWAY	1.58	2.28E-05
WNT BETA CATENIN SIGNALING	1.58	7.24E-03
ANGIOGENESIS	1.55	1.05E-02
HEDGEHOG SIGNALING	1.52	1.54E-02
KRAS SIGNALING DN	1.5	7.30E-03
PI3K AKT MTOR SIGNALING	1.39	1.54E-02
MITOTIC SPINDLE	1.39	3.60E-03
HYPOXIA	1.36	7.78E-03
NOTCH SIGNALING	1.3	6.93E-02
UV RESPONSE UP	1.29	3.10E-02
REACTIVE OXYGEN SPECIES PATHWAY	1.28	6.93E-02
ESTROGEN RESPONSE EARLY	1.18	7.31E-02
ESTROGEN RESPONSE LATE	1.09	1.55E-01
HEME METABOLISM	1.05	1.88E-01
XENOBIOTIC METABOLISM	1.01	2.23E-01
ADIPOGENESIS	0.89	3.25E-01
PANCREAS BETA CELLS	0.84	3.02E-01
GLYCOLYSIS	0.6	3.78E-01
PROTEIN SECRETION	0.6	3.78E-01
UNFOLDED PROTEIN RESPONSE	0.52	3.78E-01
CHOLESTEROL HOMEOSTASIS	-0.76	3.78E-01
PEROXISOME	-0.81	3.78E-01
MYC TARGETS V2	-0.87	3.26E-01
BILE ACID METABOLISM	-0.93	2.91E-01
DNA REPAIR	-1.03	1.82E-01
MTORC1 SIGNALING	-1.15	4.03E-02
FATTY ACID METABOLISM	-1.38	7.24E-03

G2M CHECKPOINT	-1.43	6.85E-04
SPERMATOGENESIS	-1.5	7.24E-03
ANDROGEN RESPONSE	-1.62	6.85E-04
E2F TARGETS	-1.87	2.87E-08
MYC TARGETS V1	-2.68	1.58E-10
OXIDATIVE PHOSPHORYLATION	-2.77	1.58E-10

Supplementary Table 11: Gene set enrichment analyses (GSEA) using the 'hallmark molecular signatures' for *BCL2* mRNA expression in ICR/RMH CRPC RNA sequencing cohort (n=95) NES = normalized enrichment score; FDR = false discovery rate.

Gene set enrichment analysis for *BCL2* mRNA expression (SU2C/PCF RNA sequencing cohort)		
**Pathway**	**NES**	**FDR**
MITOTIC SPINDLE	2.05	6.61E-10
G2M CHECKPOINT	1.83	6.61E-10
ALLOGRAFT REJECTION	1.81	7.53E-08
UV RESPONSE DN	1.78	1.49E-07
INTERFERON GAMMA RESPONSE	1.78	6.79E-09
APICAL JUNCTION	1.75	1.19E-07
COMPLEMENT	1.75	2.12E-07
EPITHELIAL MESENCHYMAL TRANSITION	1.73	8.08E-08
HEDGEHOG SIGNALING	1.7	3.01E-03
KRAS SIGNALING UP	1.66	2.67E-06
E2F TARGETS	1.66	2.69E-07
INFLAMMATORY RESPONSE	1.65	4.03E-05
APICAL SURFACE	1.62	6.05E-03
IL6 JAK STAT3 SIGNALING	1.61	1.67E-03
WNT BETA CATENIN SIGNALING	1.58	6.05E-03
TNFA SIGNALING VIA NFKB	1.55	1.27E-04
TGF BETA SIGNALING	1.52	7.22E-03
NOTCH SIGNALING	1.47	2.13E-02
IL2 STAT5 SIGNALING	1.46	7.01E-04
COAGULATION	1.46	5.80E-03
APOPTOSIS	1.43	3.06E-03
INTERFERON ALPHA RESPONSE	1.41	1.08E-02
MYOGENESIS	1.36	9.36E-03
ANGIOGENESIS	1.32	7.20E-02
P53 PATHWAY	1.3	1.43E-02
PI3K AKT MTOR SIGNALING	1.21	8.67E-02
ESTROGEN RESPONSE EARLY	1.19	6.38E-02
KRAS SIGNALING DN	1.16	1.26E-01
SPERMATOGENESIS	1.15	1.32E-01
PANCREAS BETA CELLS	1.15	1.62E-01
HYPOXIA	1.01	2.62E-01
ESTROGEN RESPONSE LATE	0.96	3.13E-01
HEME METABOLISM	0.96	3.13E-01
UV RESPONSE UP	0.92	3.49E-01
PROTEIN SECRETION	0.8	4.17E-01
DNA REPAIR	0.78	4.20E-01
XENOBIOTIC METABOLISM	0.6	4.41E-01
MTORC1 SIGNALING	0.57	4.41E-01
UNFOLDED PROTEIN RESPONSE	-0.84	4.20E-01
GLYCOLYSIS	-0.9	4.20E-01
MYC TARGETS V1	-1.11	9.15E-02
BILE ACID METABOLISM	-1.13	1.04E-01
CHOLESTEROL HOMEOSTASIS	-1.15	9.94E-02
PEROXISOME	-1.25	3.98E-02
ADIPOGENESIS	-1.28	7.55E-03

REACTIVE OXYGEN SPECIES PATHWAY	-1.32	4.30E-02
MYC TARGETS V2	-1.58	4.15E-03
ANDROGEN RESPONSE	-1.74	1.16E-04
FATTY ACID METABOLISM	-2.28	6.61E-10
OXIDATIVE PHOSPHORYLATION	-3.68	6.61E-10

Supplementary Table 12: Gene set enrichment analyses (GSEA) using the 'hallmark molecular signatures' for *BCL2* mRNA expression in SU2C/PCF CRPC RNA sequencing cohort (n=159). NES = normalized enrichment score; FDR = false discovery rate.

No	Protein Expression (H-score)					Histopathology
	BCL2	AR-NTD	CD56	CgA	SYP	
3	0	106	80	0	0	Usual prostate adenocarcinoma with neuroendocrine differentiation
5	0	50	0	0	0	Adenocarcinoma
8	0	250	0	0	0	Adenocarcinoma
31	0	140	0	0	90	Usual prostate adenocarcinoma with neuroendocrine differentiation
39	0	210	0	0	0	Adenocarcinoma
74	0	130	0	0	0	Adenocarcinoma
80	0	270	0	0	0	Adenocarcinoma
182	0	160	0	0	0	Adenocarcinoma
21	1	130	0	0	50	Large cell neuroendocrine carcinoma
51	1	90	0	80	30	Usual prostate adenocarcinoma with neuroendocrine differentiation
154	1	200	0	15	10	Adenocarcinoma
78	2	190	0	0	60	Usual prostate adenocarcinoma with neuroendocrine differentiation
161	2	0	300	90	90	Small cell carcinoma
178	3	225	0	180	0	Mixed small cell neuroendocrine carcinoma-acinar carcinoma
48	5	250	0	0	0	Adenocarcinoma
1	10	80	0	75	0	Usual prostate adenocarcinoma with neuroendocrine differentiation
9	20	250	0	0	0	Adenocarcinoma
49	35	190	0	0	0	Adenocarcinoma
30	50	140	0	0	0	Adenocarcinoma
22	70	250	0	0	0	Adenocarcinoma
57	80	210	0	0	0	Adenocarcinoma
27	85	280	0	0	70	Large cell neuroendocrine carcinoma
146	90	0	85	0	20	Large cell neuroendocrine carcinoma
156	120	20	300	60	40	Small cell carcinoma
68	130	0	30	190	20	Small cell carcinoma
150	200	0	0	50	30	Small cell carcinoma

Supplementary Table 13: Histopathological classification and neuroendocrine marker protein expression (H-score). IHC for CD56, Chromogranin A (CgA) and Synaptophysin (SYP) was performed in a subset of mCRPC tumours with pre-existing AR-NTD and BCL2 staining (ICR/RMH CRPC IHC cohort, n=26).

Gene set enrichment analysis comparing high BCL2/high AR vs. low BCL2/high AR (SU2C/WCDT CRPC RNA sequencing cohort)		
**Pathway**	**FDR**	**NES**
HEME METABOLISM	1.67	6.1E-03
MITOTIC SPINDLE	1.59	2.1E-02
G2M CHECKPOINT	1.56	2.2E-02
INFLAMMATORY RESPONSE	1.47	1.1E-01
UV RESPONSE DN	1.46	1.6E-01
SPERMATOGENESIS	1.36	2.9E-01
KRAS SIGNALING DN	1.33	3.0E-01
E2F TARGETS	1.31	3.1E-01
INTERFERON GAMMA RESPONSE	1.22	4.5E-01
MYOGENESIS	1.16	5.5E-01
COMPLEMENT	1.15	5.6E-01
INTERFERON ALPHA RESPONSE	1.14	6.0E-01
ALLOGRAFT REJECTION	1.09	6.5E-01
WNT BETA CATENIN SIGNALING	1.09	6.9E-01
IL6 JAK STAT3 SIGNALING	1.07	7.0E-01
ESTROGEN RESPONSE LATE	1.05	7.2E-01
NOTCH SIGNALING	1.04	7.5E-01
EPITHELIAL MESENCHYMAL TRANSITION	1.02	7.5E-01
APICAL JUNCTION	1.01	7.6E-01
KRAS SIGNALING UP	1.01	7.6E-01
HYPOXIA	0.98	8.0E-01
APOPTOSIS	0.93	8.5E-01
ESTROGEN RESPONSE EARLY	0.88	9.1E-01
IL2 STAT5 SIGNALING	0.87	9.2E-01
APICAL SURFACE	0.83	9.0E-01
TGF BETA SIGNALING	0.75	9.5E-01
HEDGEHOG SIGNALING	0.62	9.8E-01
ANGIOGENESIS	-0.89	8.7E-01
TNFA SIGNALING VIA NFKB	-0.91	9.4E-01
PI3K AKT MTOR SIGNALING	-0.98	7.7E-01
PANCREAS BETA CELLS	-1.22	5.3E-01
MYC TARGETS V2	-1.23	4.6E-01
UV RESPONSE UP	-1.32	2.3E-01
P53 PATHWAY	-1.35	1.8E-01
DNA REPAIR	-1.36	1.5E-01
GLYCOLYSIS	-1.46	5.4E-02
REACTIVE OXYGEN SPECIES PATHWAY	-1.51	2.7E-01
PROTEIN SECRETION	-1.62	4.5E-02
CHOLESTEROL HOMEOSTASIS	-1.68	6.6E-02
ADIPOGENESIS	-1.74	4.8E-04
BILE ACID METABOLISM	-1.77	1.1E-02
PEROXISOME	-1.78	1.0E-02

COAGULATION	-1.81	1.3E-03
XENOBIOTIC METABOLISM	-1.83	8.6E-05
FATTY ACID METABOLISM	-1.94	9.8E-05
MTORC1 SIGNALING	-1.99	2.6E-06
UNFOLDED PROTEIN RESPONSE	-2.22	1.0E-05
MYC TARGETS V1	-2.26	1.4E-09
ANDROGEN RESPONSE	-2.45	2.0E-07
OXIDATIVE PHOSPHORYLATION	-2.68	4.4E-17

Supplementary Table 14: Gene set enrichment analyses (GSEA) using the 'hallmark molecular signatures' in samples with high BCL2/high AR (n=6) vs. low BCL2/high AR (n=99) from the SU2C/WCDT CRPC RNA sequencing cohort. BCL2 cut off: ≥ 90th percentile expression, AR cut off: median expression. NES = normalized enrichment score; FDR = false discovery rate.

Supplementary methods

Patients and tissue samples

The Institute of Cancer Research (ICR)/Royal Marsden Hospital (RMH) castration-resistant prostate cancer (CRPC) IHC cohort: All patients had metastatic CRPC (mCRPC), were treated at the RMH, provided written informed consent, and were enrolled in protocols approved by the RMH ethics review committee. For the matched same-patient cohort, castration-sensitive prostate cancer (CSPC) samples were retrieved from referring hospitals and all patients had adenocarcinoma at diagnosis. CRPC tissue was collected from metastatic biopsies. In this cohort, 247 biopsies from 187 patients were analysed, including 60 matched same-patient CSPC tumours.

University of Washington (UW)/Fred Hutchinson Cancer Center (FHCC) CRPC IHC cohort: Samples were obtained from patients who died of metastatic CRPC and had signed written consent for a rapid autopsy as previously described (1). Samples were analysed on a tissue microarray. In this cohort, 485 spatially separated samples in 177 CRPC biopsies from 58 patients were analysed.

Immunohistochemistry antibody optimisation and validation

Immunohistochemistry (IHC) for neuroendocrine markers was performed using the following antibodies: mouse anti-CD56 monoclonal antibody (Agilent; #M7304), mouse anti-Chromogranin monoclonal antibody (Agilent; #M0869) and mouse anti-Synaptophysin monoclonal antibody (Leica Biosystems, #NCL-L-SYNAP-299). Briefly, antigen retrieval was performed with tris-EDTA (pH8.1) for CD56 and Chromogranin, and citrate buffer (pH6) for Synaptophysin in the microwave for 18 minutes at 800W. The primary antibodies were diluted at 1:250 (CD56) and 1:200 (Chromogranin and Synaptophysin), incubated for 1 hour and staining was visualised using the REAL EnVision Detection System (Agilent; #K4061 – product discontinued). Sections were counterstained with haematoxylin. Normal appendix tissue was used as a positive control for CD56 and normal pancreas for Chromogranin and Synaptophysin. Mouse IgGs were used as negative controls.

IHC for MCL1 was performed using the rabbit anti-MCL1 polyclonal antibody (Proteintech; #16225-1-AP). Briefly, antigen retrieval was performed with citrate buffer (pH6) in a pressure cooker (Menapath Antigen Access Unit, Menarini diagnostics, Berkshire, UK). The primary antibody was diluted at 1:500, incubated for 1 hour and staining was visualised using the EnVision Detection System (Agilent; #K4061 – product discontinued). Sections were counterstained with haematoxylin. Prostate tissue was used as a positive control. Cell pellets from DU145 cells treated with MCL1 siRNA were used to confirm specificity of the antibody. Rabbit IgGs were used as negative controls (**Supplementary Fig. 1A-C**).

BCLXL IHC was performed using the rabbit anti-BCLXL monoclonal antibody (Cell Signaling Technology; #2764). Briefly, antigen retrieval was performed for 30 minutes with Bond ER1 solution (Leica Biosystems, #AR9961) and anti-BCLXL antibody (1:1000 dilution) incubated with tissue for 15 minutes and the reaction visualized using Bond Polymer Refine (#DS9800, Leica Biosystems) system. Kidney tissue was used as a positive control. Cell pellets from DU145 cells treated with control and BCL2L1 siRNA were used to confirm specificity of the antibody for BCLXL (Supplementary Fig. 1B). Rabbit IgGs were used as negative controls (**Supplementary Fig. 1D-F**).

Immunohistochemistry protein quantification

Protein quantification was determined by a histopathologist (B.G. or D.N.R.) blinded to clinical data using the H-score method; [(% of absent staining x 0] + (% of weak staining x 1) + (% of moderate staining x 2) + (% of strong staining x 3), resulting in a score between 0 and 300 (2). H-scores were used for the ICR/RMH CRPC cohort and PDX models. In addition, selected stained slides were scanned on a VS200 Digital Slide Scanner (Olympus, Japan) and a pathologist-supervised machine learning algorithm (HALO AI, Indica Labs) was trained to differentiate prostate cancer cells from benign stroma. Colour deconvolution for DAB and haematoxylin staining was performed, and cell recognition and nuclear segmentation was optimised. The analysis algorithm was adjusted to provide continuous data for both nuclear and cytoplasmic optical density (OD) for DAB, providing a value between 0 (no staining) and 1 (black). Optical density (OD) scores were used for the UW/FHCC cohort and LuCaP PDX series, allowing automated batch analysis of tissue microarrays. For validation purposes, a subset of the samples (n=77) scored by OD were also visually scored by H-score, revealing a

strong positive correlation between the different methods of quantification for both nuclear AR-NTD (r=0.963, 95% confidence interval [CI] 0.942 - 0.977, p<0.0001) and cytoplasmic BCL2 (r=0.699, 95% CI 0.559 - 0.800, p<0.0001) (**Supplementary Fig. 2A and B**). AR-negativity was defined as a nuclear AR-NTD H-score ≤ 20. The equivalent cut-off for AR-NTD OD (≤ 0.013) was calculated using a simple linear regression equation from the correlation between H-score and OD (H-score = (442.25 x OD) + 14.457) (**Supplementary Fig. 2A**). BCL2 positivity was defined as a cytoplasmic BCL2 H-score >20. The equivalent cut-off for BCL2 OD (>0.033) was calculated using simple linear regression equation from the correlation between H-score and OD (H-score = (872.7 x OD) + 8.614 (**Supplementary Fig**. **2B**).

Western Blotting

To determine the basal expression of protein, cells were plated in 6 well plates and allowed to settle for 48 hours prior to collection. For siRNA transfection, NCI-H660 was plated in 6 well plate, allowed to settle for 48 hours and then transfected for 72 hours with siRNA prior to collection. For adherent cells, media was aspirated from each well and cells were washed with PBS. Cell lysis was undertaken with Pierce RIPA buffer supplemented with a Pierce Protease and Phosphatase inhibitor tablet (Thermo Fisher Scientific). Lysate was collected using a cell scraper and transferred to 1.5 ml Eppendorf tubes on ice. For the NCI-H660 (suspension cells), media was aspirated and then transferred to a 15 ml falcon tube, followed by centrifugation. The cell pellet was resuspended in PBS, then centrifuged, prior to the addition of RIPA buffer. After brief sonication (10 seconds), the samples were centrifuged for 15 minutes at 4 °C. Protein concentrations were determined using Pierce BCA Protein Assay Kit (Thermo Fisher Scientific). 25µg of protein was separated on 4-12% NuPAGE Bis-Tris gel plates (Invitrogen) using electrophoresis and then transferred to Immobilon-P PVDF membranes (pore size 0.45 µm). Membranes were incubated with primary and then secondary antibodies (**Supplementary Table 5**) in 5% milk in TBS and Tween20 (Sigma-Aldrich). Chemiluminescence was undertaken using the ChemiDoc Touch Imaging System (Bio-Rad).

Cell line studies

Benign prostate and prostate cancer cell lines were cultured in the recommended media at 37 °C in 5% CO^2 (**Supplementary Table 6**), STR profiled using the cell line authentication service (Eurofins Medigenomix) and tested for mycoplasma with the VenorGem One Step PCR

Kit (Cambio). For adherent lines, cells were detached from culture flasks by trypsinization (TrypLE Express, Thermo Fisher Scientific). For drug experiments, cells were plated in 96 well plates and allowed to settle for 48 hours prior to treatment. The impact on caspase 3/7 activity and cell viability was determined by the Caspase 3/7-Glo and CellTiter-Glo (Promega) respectively. Drug experiments were performed in technical triplicate with three biological replicates, apart from the caspase 3/7 analysis with navitoclax/AZD5991 treatment which was undertaken as a single biological replicate (three technical replicates). The LNCaP-iGFP and LNCaP-iSnail cells were generated as previously described (3). LNCaP-iGFP and LNCaP-iSnail cells were plated in 6 well plates and allowed to settle for 48 hours prior to addition of doxycycline (1µg/mL). Doxycycline was refreshed every 48 hours.

Patient-derived xenograft in vitro studies

ICR/RMH patient-derived xenograft (PDX) CP model: The CP336 (and CP336c) PDX was derived from a human CRPC lymph node biopsy using the same methods as for CP50 and CP89 (4-6). The patient treatment history for CP336 is detailed in **Figure 8A**. The CP336c patient derived xenograft-organoid (PDX-O) was generated from the CP336c PDX using methods previously described (6). Organoids were formed in Matrigel® matrix (356231, Corning) domes in 24-well plate and then re-seeded into 96-well plates for drug experiments. After at least 24 hours, organoids were treated with vehicle (DMSO 0.01%), venetoclax (1 uM), A-1331852 (100 nM), navitoclax (1 uM), AZD5991 (1 uM), and a combination of AZD5991 (1 uM) and navitoclax (1 uM). The impact on caspase 3/7 activity (6 hours) and organoid viability (24 and 96 hours) was determined by the Caspase-Glo 3/7 3D and CellTiter-Glo 3D assays (Promega) respectively. Drug experiments were performed in biological triplicate with 5 technical replicates.

LuCaP models: Prostate cancer 3D organoids and primary cultures were derived from fresh PDX tumours grown in castrated mice. Minced tumour fragments were digested in ACCUMAX (Thermo Fisher Scientific, Accumax-Cell Aggregate Dissociation Medium, Cat.No: 00-4666-56), resuspended in DMEM/F12 plus 10% FBS and passed through a 250-µm cell strainer (Thermo Scientific Pierce Tissue Strainers, Cat. No: 87791) to remove tissue debris and obtain smaller cell clusters. A) For organoids, cells were plated on Matrigel covered tissue cultured plates (Corning Matrigel, Growth Factor Reduced Basement Membrane Matrix, LDEV-free,

Cat: 354230) and prostate-organoid specific media. After 5-7 days, developed organoids were transferred to 96 well plates and treated with venetoclax and DMSO for 4 days. B) For primary cultures, cell clusters were transferred to 96 well plates and treated with venetoclax and DMSO for 4 days. Cell viability was assessed with the CellTiter-Glo 3D Cell Viability Assay (Promega). Three biological replicates were assessed per treatment group.

RNA sequencing data

ICR/RMH and SU2C/PCF RNA sequencing dataset analysis (TopHat pipeline): CRPC transcriptomes generated by the International Stand Up To Cancer/Prostate Cancer Foundation (SU2C/PCF) Dream Team, were downloaded and reanalysed (7). Paired-end transcriptome sequencing reads for each of the SU2C/PCF (n=159) and ICR/RMH (n=95) cohorts were aligned to the human reference genome (GRCh37/hg19) using Tophat2 (v2.0.7) (8). Gene expression, Fragments Per Kilobase of transcript per Million mapped reads (FPKM), was calculated using Cufflinks. The top expressed genes (n=15000) were analysed for each cohort respectively.

LuCaP PDXs: RNA sequencing data analysis was performed as previously described (9).

Methylation analysis

LuCaP PDXs: Genome scale methylation analyses of 36 LuCaP PDX DNAs were carried out using Infinium MethylationEPIC BeadChip arrays (Illumina) as described previously (10). Raw data were analyzed in the minfi package in R, and samples were normalized using the subset-quantile within array normalization method (11, 12). Probes with a detection p-value of >0.01 in 50% or more of samples and probes that contained a SNP at the CpG interrogation site or at the single nucleotide extension were removed. DNA methylation pattern at the *BCL2* locus were inspected in the Integrative Genomics Viewer (IGV) and methylation levels of the differentially methylated regions (DMRs) were extracted (13). EPIC array data are publicly available on Gene Expression Omnibus (GEO), accession number GSE227853. Of note, methylation, RNA sequencing and protein (IHC) analysis for each LuCaP model were not always carried out on the same PDX passage.

SU2C/WCDT cohort: Methylation data was visualized by first extracting coordinate and percent methylated information from the methylation call format into bedGraph format and

then converting to bigWigs using bedGraphToBigWig (14). BigWig files were then view in the integrative genomics viewer using default setting (13).

Chromatin immunoprecipitation (ChIP)

For publicly available ASCL1 ChIP-seq data Fastq reads were aligned to UCSC hg19 using bwa 0.7.17-r1188 and filtered for alignments with a MAPQ score of at least 30 with samtools 1.7 (15). Duplicates were marked with picard MarkDuplicates version 2.24.1. Tracks were viewed in the Integrative Genomics Viewer (IGV).

Statistical Analyses and Bioinformatics

A two-sided p value <0.05 was deemed to be statistically significant. The difference in AR-NTD protein expression between matched same-patient CSPC and CRPC biopsies was analysed using the Wilcoxon matched-pairs signed rank test (**Fig. 1C**). Overall survival (OS) and the time on therapy analyses were performed using Kaplan-Meier methodology (**Fig. 1E, 2G, 3B-F, Supplementary Fig. 3B, 4B, 8C, 9F, 9G**). An H-score of less than or equal to 20 was used to define AR-NTD negative (nuclear) and BCL2 negative (cytoplasmic) disease. This cut point was used to compare OS, as well as the time on ARSI, between patients using the log-rank test. OS from CRPC diagnosis was defined as the time from the documented date of prostate cancer progression (PSA, radiological or change of treatment due to progression) whilst receiving a luteinising hormone-releasing hormone (LHRH) agonist alone or in combination with an anti-androgen if anti-androgen was started before/or with the LHRH agonist until the date of death or last follow-up/contact. OS from CRPC biopsy, or from starting ARSI or docetaxel, was defined as the time from the date of CRPC biopsy, and the date of initiation of therapy respectively. The time on ARSI or docetaxel was defined as the duration in months from starting the therapy until the date of cessation.

The Mann-Whitney U test was used to compare the cytoplasmic protein expression of BCL2 between AR positive/high and AR negative/low tumours (**Fig. 2B, E, F, I and Supplementary Fig. 5C**). For the UW/FHCC cohort, an average AR and BCL2 expression (mean of optical density scores) was calculated for each tumour prior to analysis (**Fig. 2I**). The Fisher's exact test was used to evaluate the association between BCL2 (negative and positive) and AR (negative and positive) expression status (**Fig. 2C and Supplementary Fig. 5D**). The impact

of venetoclax on caspase 3/7 activity was compared against vehicle (DMSO) for each cell line using the unpaired t test (two-tailed) (**Fig. 9B**). The impact of each drug treatment on caspase 3/7 and organoid/primary cell culture viability was compared with vehicle using Dunnett's multiple comparison test (**Fig. 9C and E**). The impact of navitoclax and AZD5991 on caspase 3/7 activity and cell viability was compared with vehicle in the cell lines using the unpaired t test (two-tailed) (**Supplementary Fig. 14**). The difference in *BCL2*, *MCL1*, *BCLXL* and *AR* mRNA expression between tumours with high *BCL2* (mRNA expression ≥90th percentile) and low/normal *BCL2* (mRNA expression <90th percentile) was analysed using the Mann-Whitney U test (**Fig. 9D and Supplementary Fig. 13C and D**). The number of docetaxel cycles received was compared using the Mann Whitney-U test (**Supplementary Fig. 9C and 9E**). For the RNA sequencing cohorts, the Spearman's rank correlation coefficient (Spearman's rho) between each gene's expression (FPKM) and *BCL2* expression (FPKM) was calculated, and subsequently used for pathway analysis. Pathway analysis was performed using the Gene Set Enrichment Analysis (GSEA) Pre-Ranked algorithm from GSEA software (v4.1.0). GSEA Pre-Ranked results were obtained using the H collection of Hallmark gene sets (MSigDB v7.0), with default parameters (**Fig. 4A and B**).

For the SU2C/WCDT cohort, tumours were classified into AR/Neuroendocrine molecular subtypes based on hierarchical clustering of AR, NEUROI and NEUROII gene set expression (log_2 transcript per million (TPM) +1) using the pheatmap package in R (9) (**Fig. 6F and Supplementary Fig. 12 A and B**). To further investigate BCL2 function in the context of AR positive tumours, we obtained a larger cohort of mCRPC comprising 210 patients from WCDT, characterized by RNA sequencing (16). We selected the 90th percentile of *BCL2* expression as the cut-point to distinguish between Low BCL2 (n=189) and High BCL2 (n=21) groups. Median expression was used to divide AR high and AR low cases. This resulted in the inclusion of 6 High BCL2\High AR and 99 Low BCL2/High AR samples, which were compared using GSEA with the FGSEA package in R (**Supplementary Fig. 12B and Supplementary Table 14**) (17).

To evaluate the association between IHC quantification methods (visual H-score vs automated optical density), Spearman's rank correlation was utilised (**Supplementary Fig. 2A and 2B**). The difference in AR-NTD and BCL2 expression between the sites of biopsy (ICR/RMH cohort) was analysed using Tukey's multiple comparison test (**Supplementary Fig. 3A and 4A**).

The difference between methylation indices for BCL2 positive/high and BCL2 negative/low tumours was analysed using the Mann-Whitney U test (**Fig. 6B and E**).

To account for the hierarchical structure of the UW/FHCC cohort (samples nested in biopsies, biopsies nested in patients), Bayesian generalised linear multilevel models (gaussian family, identity link function) were fitted to model log-transformed AR-NTD expression and log-transformed BCL2 expression, respectively. Group-level effects for biopsies and patients were included to address the nesting. A weakly informative Student-t prior with 3 degrees of freedom and a scale parameter of 2.5 was assumed for the population-level intercept, where the t distribution was centred around the median of log-transformed AR-NTD expression and log-transformed BCL2 expression, respectively; for the residual standard deviation and the group-level standard deviations, which were restricted to be non-negative, weakly informative half Student-t priors with 3 degrees of freedom and a scale parameter of 2.5 were used. Posterior draws were obtained from Markov Chain Monte Carlo (MCMC) methods using the No-U-Turn Sampler (18). Three Markov chains were generated with 30,000 iterations per chain and a warmup of 3,000 iterations. Every third value was retained to avoid autocorrelation. Convergence was considered to have been reached if Vehtari's rank-based $\hat{R}$ was less than 1.01 (19). Intra-cluster correlation (for the patient and biopsy level) was calculated as the ratio of the between-cluster variance to the total variance (**Supplementary Table 7 and 8**). Binary AR-NTD expression (>0.013) was added as a population-level effect to the BCL2 intercept-only model to assess the association between AR-NTD and BCL2 expression. An improper flat prior over the reals was assumed for AR-NTD expression, whereas priors for intercept and standard deviations as well as number of chains, warmup and thinning remained the same as for the intercept-only model. Posterior distributions of BCL2 expression by AR-NTD expression and of the pairwise difference in BCL2 expression between AR-NTD low and high expressing groups were drawn (**Supplementary Fig. 6B**). In a separate analysis, the models for AR-NTD and BCL2 expression were extended by a population-level effect for tissue type. An improper flat prior over the reals was assumed for tissue type, whereas priors for intercept and standard deviations as well as number of chains, warmup and thinning remained the same as for the intercept-only models. Posterior distributions of AR-NTD and BCL2 expression by tissue type (**Supplementary Fig. 7A and C**) and posterior distributions of the pairwise differences between tissue types (**Supplementary**

Fig. 7B and D; Supplementary Table 9 and 10) were drawn. All Bayesian models were fitted in R version 4.2.1 using the *brms* package (version 2.18.0) (20).

References

1. Bluemn EG, et al. Androgen Receptor Pathway-Independent Prostate Cancer Is Sustained through FGF Signaling. *Cancer Cell.* 2017;32(4):474-89 e6.
2. Detre S, et al. A "quickscore" method for immunohistochemical semiquantitation: validation for oestrogen receptor in breast carcinomas. *J Clin Pathol.* 1995;48(9):876-8.
3. Stylianou N, et al. A molecular portrait of epithelial-mesenchymal plasticity in prostate cancer associated with clinical outcome. *Oncogene.* 2019;38(7):913-34.
4. Welti J, et al. Targeting the p300/CBP Axis in Lethal Prostate Cancer. *Cancer Discov.* 2021;11(5):1118-37.
5. Welti J, et al. Targeting Bromodomain and Extra-Terminal (BET) Family Proteins in Castration-Resistant Prostate Cancer (CRPC). *Clin Cancer Res.* 2018;24(13):3149-62.
6. Gil V, et al. HER3 Is an Actionable Target in Advanced Prostate Cancer. *Cancer Res.* 2021;81(24):6207-18.
7. Abida W, et al. Genomic correlates of clinical outcome in advanced prostate cancer. *Proc Natl Acad Sci U S A.* 2019;116(23):11428-36.
8. Fenor de la Maza MD, et al. Immune Biomarkers in Metastatic Castration-resistant Prostate Cancer. *Eur Urol Oncol.* 2022.
9. Labrecque MP, et al. Molecular profiling stratifies diverse phenotypes of treatment-refractory metastatic castration-resistant prostate cancer. *J Clin Invest.* 2019;129(10):4492-505.
10. Pidsley R, et al. Critical evaluation of the Illumina MethylationEPIC BeadChip microarray for whole-genome DNA methylation profiling. *Genome Biol.* 2016;17(1):208.
11. Aryee MJ, et al. Minfi: a flexible and comprehensive Bioconductor package for the analysis of Infinium DNA methylation microarrays. *Bioinformatics.* 2014;30(10):1363-9.
12. Maksimovic J, et al. SWAN: Subset-quantile within array normalization for illumina infinium HumanMethylation450 BeadChips. *Genome Biol.* 2012;13(6):R44.
13. Robinson JT, et al. Integrative genomics viewer. *Nat Biotechnol.* 2011;29(1):24-6.
14. Kent WJ, et al. BigWig and BigBed: enabling browsing of large distributed datasets. *Bioinformatics.* 2010;26(17):2204-7.
15. Cejas P, et al. Subtype heterogeneity and epigenetic convergence in neuroendocrine prostate cancer. *Nat Commun.* 2021;12(1):5775.
16. Lundberg A, et al. The Genomic and Epigenomic Landscape of Double-Negative Metastatic Prostate Cancer. *Cancer Res.* 2023;83(16):2763-74.
17. Korotkevich G, et al. Fast gene set enrichment analysis. *bioRxiv.* 2021:060012.
18. Hoffman MD, and Gelman A. The No-U-Turn Sampler: Adaptively Setting Path Lengths in Hamiltonian Monte Carlo. *J Mach Learn Res.* 2014;15:1593-623.
19. Vehtari A, et al. Rank-Normalization, Folding, and Localization: An Improved R for Assessing Convergence of MCMC (with Discussion). *Bayesian Analysis.* 2021;16(2):667-718, 52.
20. Bürkner P-C. brms: An R Package for Bayesian Multilevel Models Using Stan. *Journal of Statistical Software.* 2017;80(1):1 - 28.
