# Supplementary material for: BCL2 expression is enriched in advanced prostate cancer with features of lineage plasticity
Source: J Clin Invest. 2024 Sep 17;134(18):e179998. doi: 10.1172/JCI179998 (PMC11405043; doi:10.1172/JCI179998)
Supplement: Unedited blot and gel images [file jci-134-179998-s172.pdf]

4C

Snail

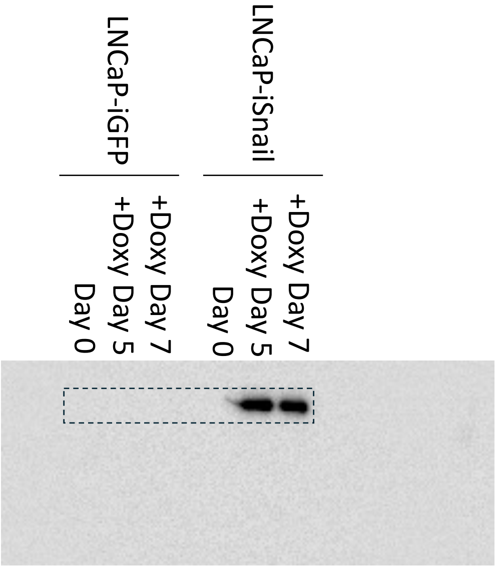

Snail	Cell Signaling	3879	Rabbit monoclonal

4C

E-Cadherin

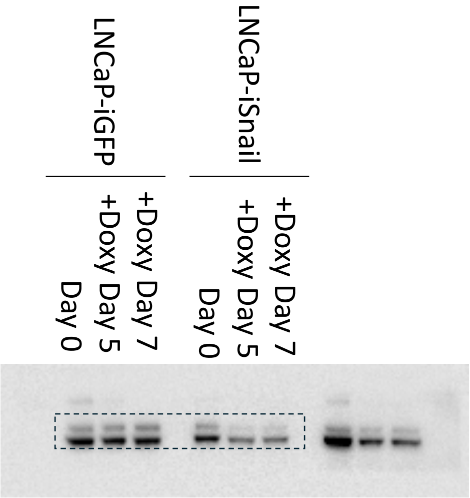

E-cadherin	Cell Signaling	14472	Mouse monoclonal

4C

Vimentin

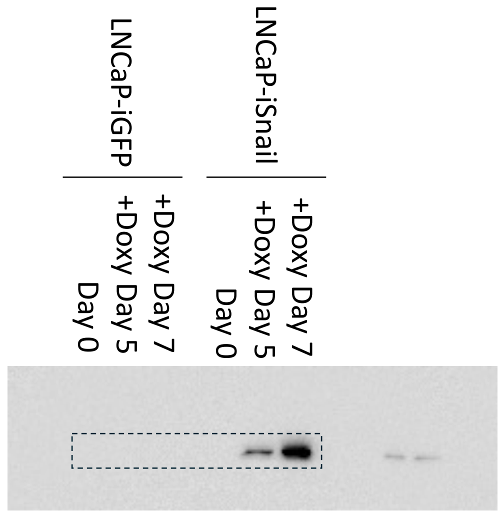

Vimentin	Cell Signaling	5741	Rabbit monoclonal

4C

BCL2

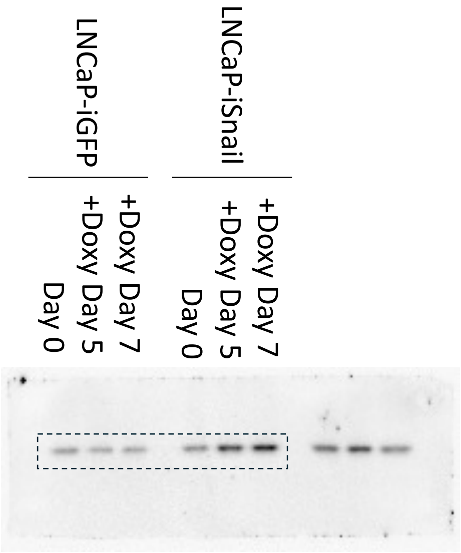

BCL2	Dako	M0887	Mouse monoclonal

4C

GAPDH

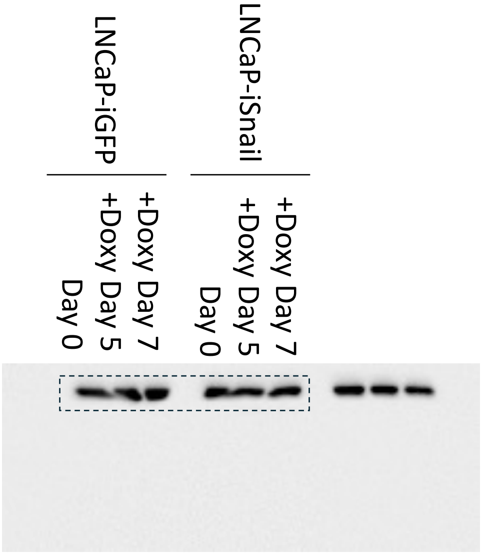

GAPDH	Santa Cruz Biotechnology	sc-32233	Mouse monoclonal

5H

AR-FL

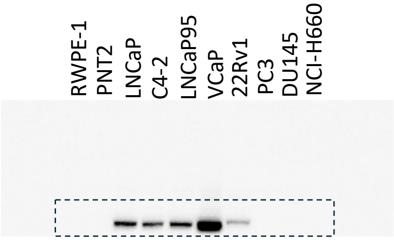

AR-NTD	Dako	M3562	Mouse monoclonal

5H

ASCL1

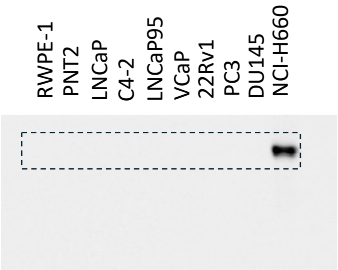

ASCL1	Abcam	Ab211327	Rabbit monoclonal

5H

BCL2

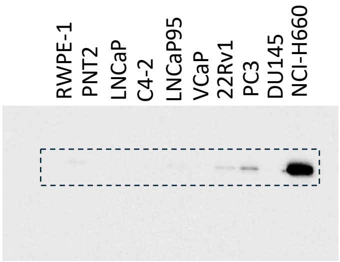

BCL2	Dako	M0887	Mouse monoclonal

5H

Vinculin

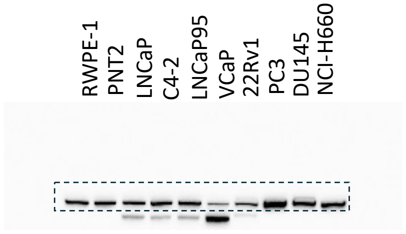

Vinculin	Santa Cruz Biotechnology	sc-73614	Mouse monoclonal

5H

GAPDH

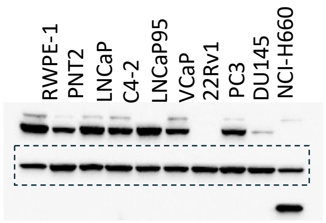

GAPDH	Santa Cruz Biotechnology	sc-32233	Mouse monoclonal

5I (top)

ASCL1

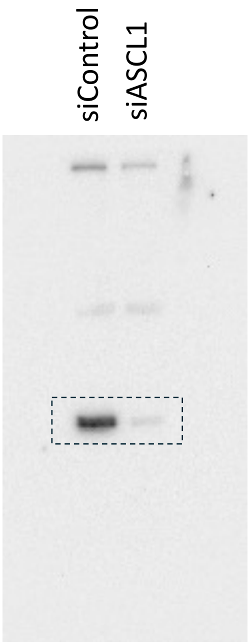

ASCL1	Abcam	Ab211327	Rabbit monoclonal

5I (top)

BCL2

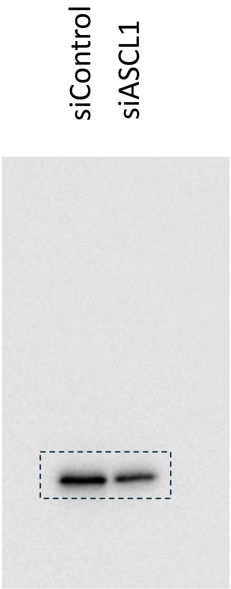

BCL2	Dako	M0887	Mouse monoclonal

5I (top)

Vinculin

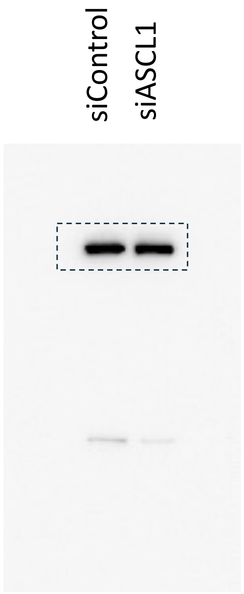

Vinculin	Santa Cruz Biotechnology	sc-73614	Mouse monoclonal

5I (bottom)

ASCL1

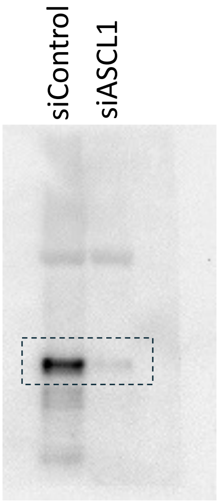

ASCL1	Abcam	Ab211327	Rabbit monoclonal

5I

BCL2

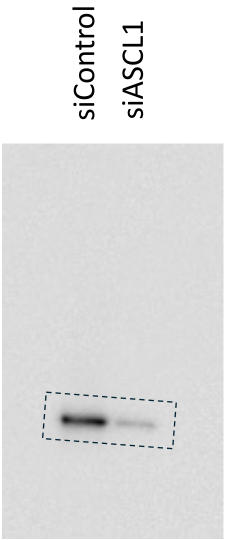

BCL2	Dako	M0887	Mouse monoclonal

5I

Vinculin

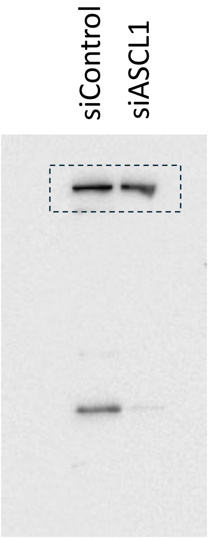

Vinculin	Santa Cruz Biotechnology	sc-73614	Mouse monoclonal

S1A

MCL1

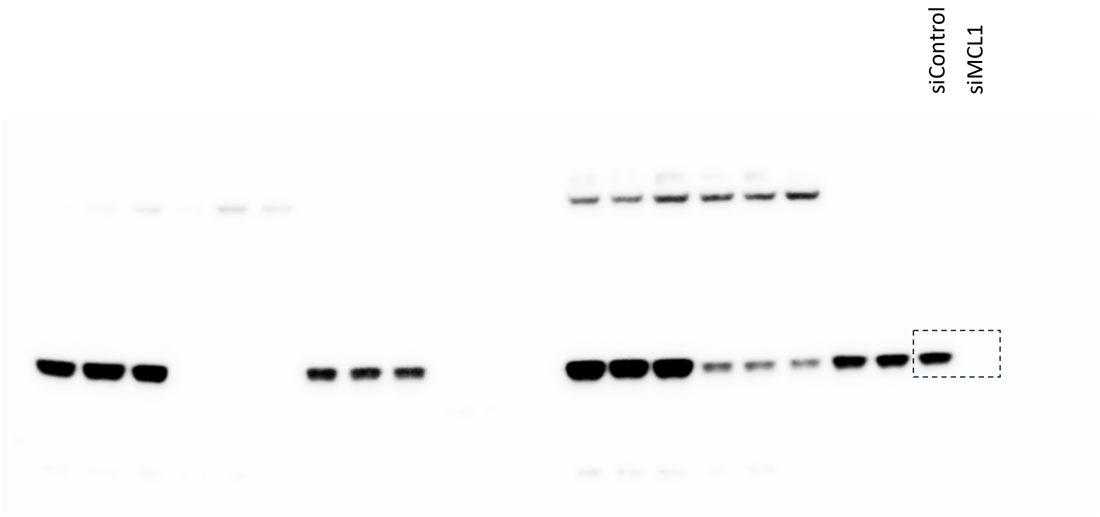

MCL1	Proteintech	16225-1-AP	Rabbit polyclonal

S1A

Vinculin

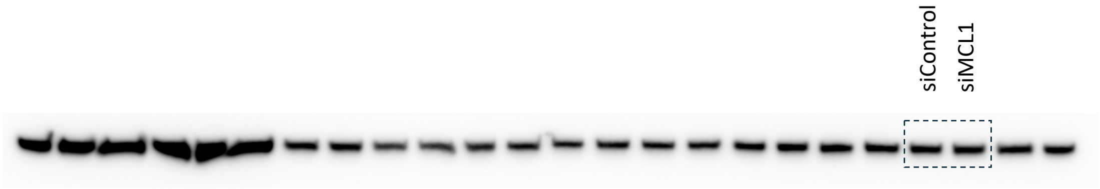

Vinculin	Santa Cruz Biotechnology	sc-73614	Mouse monoclonal

S1D

BCLXL

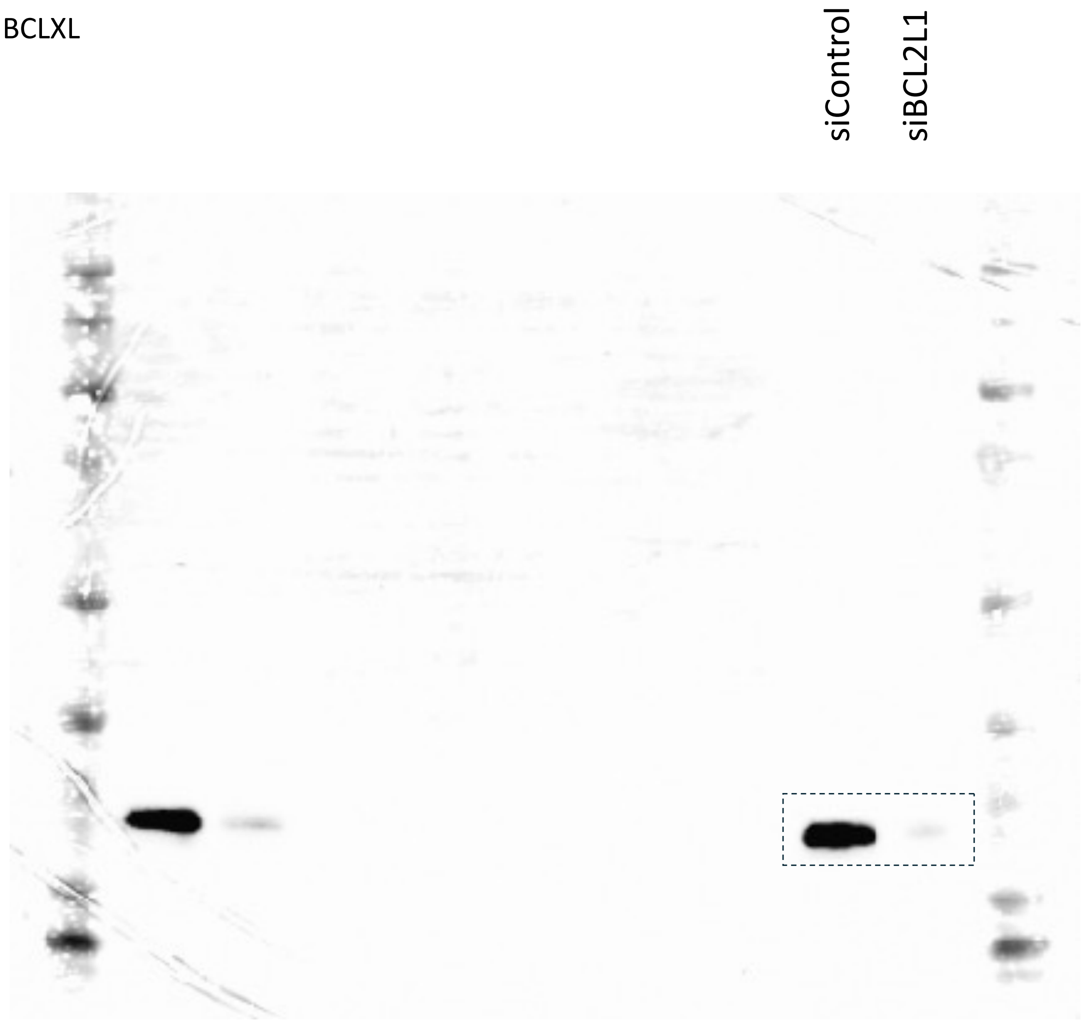

BCLXL	Cell Signaling	2764	Rabbit monoclonal

S1D

Vinculin

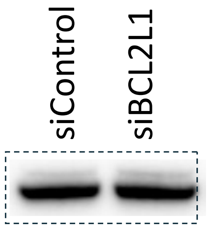

*uncropped blot not available

Vinculin	Santa Cruz Biotechnology	sc-73614	Mouse monoclonal

S10D

Snail

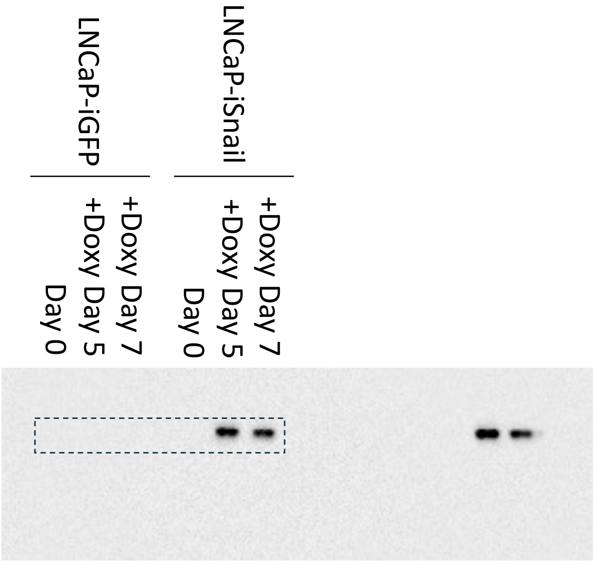

Snail	Cell Signaling	3879	Rabbit monoclonal

S10D

E-Cadherin

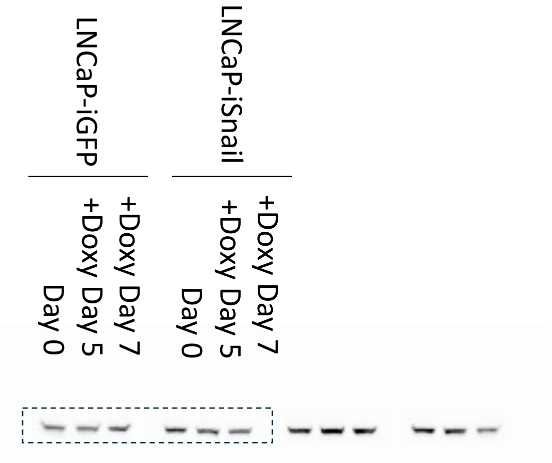

E-cadherin	Cell Signaling	14472	Mouse monoclonal

S10D

Vimentin

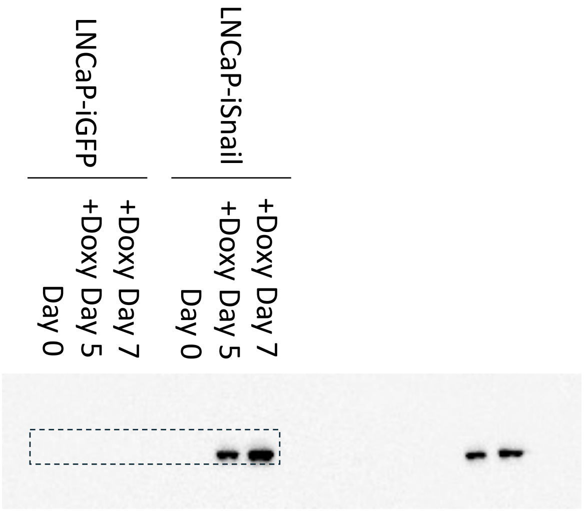

Vimentin	Cell Signaling	5741	Rabbit monoclonal

S10D

BCL2

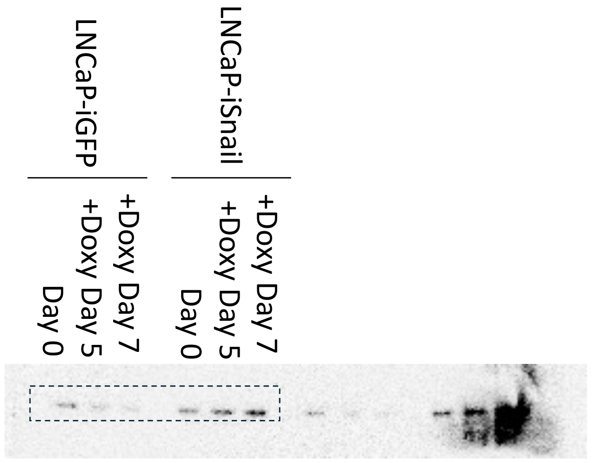

BCL2	Dako	M0887	Mouse monoclonal

S10D

GAPDH

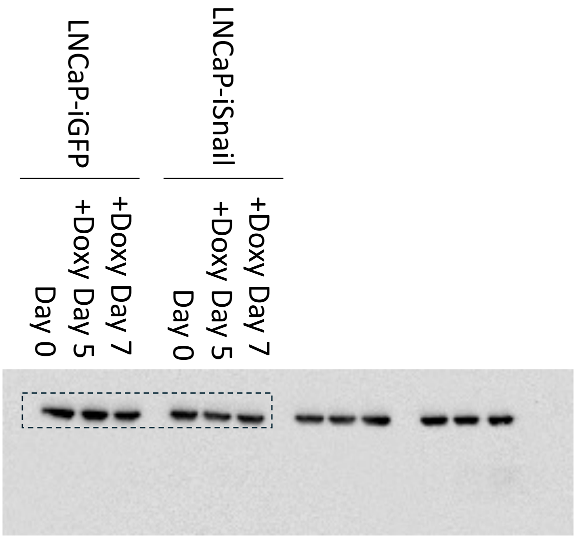

GAPDH	Santa Cruz Biotechnology	sc-32233	Mouse monoclonal
